# The first molecular detection of equine piroplasmosis in Vietnam and genetic characterization of three co-circulating genotypes of *Theileria equi*

**DOI:** 10.1007/s00436-026-08630-4

**Published:** 2026-02-05

**Authors:** Thanh Thi Ha Dao, Tamás Szűts, Ngoc Nhu Duong, Duong Thi Quy Troung, Norbert Solymosi, Nóra Takács, Sándor Hornok, Róbert Farkas

**Affiliations:** 1 Department of Parasitology, National Institute of Veterinary Research, Hanoi, Vietnam; 2 Department of Zoology, University of Veterinary Medicine Budapest, Budapest, Hungary; 3 Centre for Bioinformatics, University of Veterinary Medicine Budapest, Budapest, Hungary; 4 Department of Parasitology and Zoology, University of Veterinary Medicine Budapest, Budapest, Hungary; 5 HUN-REN-UVMB Climate Change: New Blood-sucking Parasites and Vector-borne Pathogens Research Group, Budapest, Hungary

**Keywords:** Theileria equi, Horses, Molecular identification, 18S rRNA gene, Vietnam

## Abstract

Equine piroplasmosis, caused by *Theileria equi*, *T. haneyi* and *Babesia caballi*, significantly impacts on the veterinary and economic aspects of the global horse industry. However, many countries, including Vietnam, have not yet conducted epidemiological surveys to determine the prevalence of these haemoparasites. This study aimed to detect *T. equi* and/or *B. caballi* infections in horses and to identify their genotypes. Blood samples were collected from 154 apparently healthy horses in eight districts of Hanoi, Thai Nguyen, and Son La provinces located in the northern part of the country. Twenty-four horses (15.58%, 95%CI: 10.70–22.14%) were found to be infected with *T. equi* across six districts. *Babesia caballi* was not detected. No significant association was found between gender, host age, and the number of infected horses. Phylogenetic analysis of the 18S rRNA sequences from the positive DNA samples revealed genotypes A, C, and E. These results confirm the presence of equine theileriosis in northern Vietnam, highlighting the need for nationwide studies of equine piroplasmosis involving larger sample sizes.

**Supplementary Information:**

The online version contains supplementary material available at 10.1007/s00436-026-08630-4.

## Introduction

Equine piroplasmosis is an infectious tick-borne disease caused by two main causative agents, *Theileria equi* (formerly *Babesia equi*) and *Babesia caballi* (Apicomplexa: Piroplasmida) (Rothschild 2013; Wise et al. 2013). Recently, *Theileria haneyi* has been described (Knowles et al. 2018). Preliminary studies of the small subunit ribosomal RNA gene (18S) have revealed distinct genotypes of *T. equi* and *B. caballi* (Bhoora et al. 2009; Qablan et al. 2012; Onyiche et al. 2019). The disease caused by *T. equi* and/or *B. caballi* has also been detected in donkeys, mules and zebras (Tirosh-Levy et al. 2020), as well as in non-equid species, including camels (Qablan et al. 2012), waterbucks (Githaka et al. 2014), dogs and cattle (Salim et al. 2019). While horses may clear *B. caballi* within a few years, *T. equi* persists for life unless treated (Wise et al. 2013; Scoles and Ueti 2015). Under natural and experimental conditions, more than 30 tick species belonging to various genera, including *Hyalomma*, *Rhipicephalu*s and *Dermacentor*, can transmit *T. equi* and *B. caballi* trans-stadially (Tirosh-Levy et al. 2020). Other genera, such as *Ixodes*, *Haemaphysalis*, and *Amblyomma* are suspected of being capable of transmitting these hemoparasites, but this has not been confirmed (Scoles and Ueti 2015). Transovarian transmission has been documented for *B. caballi*, meaning that infected ticks can transmit this species over several generations without re-infection (Scoles and Ueti 2015). Transplacental transmission has been reported for *T. equi*, which can result in abortion or the birth of an apparently healthy foal that is a carrier (Allsopp et al. 2007; Chhabra et al. 2012). There are numerous reports of the iatrogenic transmission of both *T. equi* and *B. caballi* via surgical needles, equipment, and blood transfusions (Rothschild 2013; Wise et al. 2013). The incubation period after infection ranges between 12 and 19 days for *T. equi* and from 10 to 30 days for *B. caballi* (Knowles et al. 2018). Equine piroplasmosis is traditionally classified as peracute, acute or chronic forms. However, many infected horses remain asymptomatic or exhibit no specific clinical signs, such as high fever, tachycardia, anorexia, anaemia and jaundice. The peracute form can be life-threatening (Rothschild 2013; Wise et al. 2013; Tirosh-Levy et al. 2020). The disease caused by *T. equi* and/or *B. caballi* is endemic in several African, Asian, American and European countries (Onyiche et al. 2019; Tirosh-Levy et al. 2020), as seemingly healthy infected horses are carriers of these pathogens between endemic and non-endemic regions (Chauvin et al. 2009). Equine piroplasmosis has a significant veterinary and economic impact on the horse industry worldwide (Tamzali 2013; Tirosh-Levy et al. 2020). Accordingly, equine piroplasmosis is a reportable disease worldwide, as defined by the World Organisation for Animal Health (WOAH) (https://www.woah.org). Currently, only a few countries, including Japan, New Zealand, Iceland and Ireland, are officially considered to be free from this parasitosis (Mendoza et al. 2024).

Equine piroplasmosis is known to be distributed worldwide (Tirosh-Levy et al. 2020), including many Asian countries (Kamyingkird et al. 2014; Nugraha et al. 2018; Wang et al. 2019; Kumar et al. 2020; Khaing et al. 2025). However, this important parasitic disease has not previously been reported in Vietnam. This study aimed to investigate the occurrence of piroplasmosis in Vietnamese horses using a PCR-based method, which is much more sensitive and specific than traditional smear examinations or serology (Mendoza et al. 2024).

## Materials and methods

### Study areas and sample collection

A total of 154 horses were examined in eight districts of Hanoi, Thai Nguyen, and Son La provinces located in the northern part of Vietnam. The animals were born in Vietnam and used by their owners to carry wood or work in rice fields. They were kept in communal pastures together with cattle, water buffalo, and small ruminants almost year-round where hard tick species were present, exposing them frequently to tick bites.

Five ml blood samples were collected from the jugular vein of each horse in 2022 and 2023 into EDTA-coated vacutainer tubes, then transferred to the laboratory in iceboxes. This study`s protocol followed the QCVN 01–83:2011/BNN&PTNT (https://cucthuy.gov.vn/en/tieu-chuan-quy-chuan/-/standards/detail/77582) and was approved by the Proposal Committee of the Ministry of Science and Technology of Vietnam (No. 02/2022/HD-NDT). All samples were taken from apparently healthy horses. Furthermore, a questionnaire was used to gather information about the main characteristics of each farm and sampled horse (see Supporting Information).

### DNA extraction, polymerase chain reaction (PCR), and sequence analysis

Genomic DNA was extracted from 200 µl of each blood sample according to the manufacturer’s instructions (QIAGEN DNA Blood Mini-Kit, Germany) in the Department of Parasitology, National Institute of Veterinary Research, Hanoi. The DNA samples were stored at − 20^◦^C until use.

Conventional PCR was performed using the forward primer BJ1 5’- GTC TTG TAA TTG GAA TGA TGG– 3’ and the reward primer BN2 5’ –TAG TTT ATG GTT AGG ACT ACG − 3’ to amplify a fragment of the 18S rRNA gene approximately 500 bp in length (Casati et al. 2006). Five µl of extracted DNA were added to 20 µl of reaction mixture containing 1.0 U HotStart Taq Plus DNA Polymerase (5 U/µl) (QIAGEN, Hilden, Germany), 0.5 µl dNTP Mix (10mM), 0.5 µl of each primer (50 µM), 2.5 µl of 10x Coral Load PCR buffer (15mM MgCl_2_ included), and 15.8 µl double-distilled water.

After an initial denaturation step at 95 °C for 10 min, 40 cycles of denaturation at 95 °C for 30 s, annealing at 54 °C for 30 s and extension at 72 °C for 40 s were performed. A final extension step was then performed at 72 °C for five min., after which the sample was kept at 4 °C. DNA of *Babesia* sp. was used as a positive control. The PCR products were then electrophoresed in 1.5% agarose gel (100 V, 50 min), stained with ethidium-bromide and visualised under ultraviolet light.

The purification and sequencing of the positive PCR products were performed by Eurofins Biomi Ltd. (Gödöllő, Hungary). All sequences were aligned with references using NCBI BLAST, at the National Institutes of Health, USA (http://www.ncbi.nlm.nih.gov). The *T. equi* 18S rRNA sequences obtained in this study have been deposited in GenBank under the accession numbers PV688145–PV688154 and PX369340–PX369353.

### Phylogenetic analysis

To test the placement of the Vietnamese 18S rRNA samples, we used the sequence list and clade assortment from Tirosh-Levy et al. (2020) (see Supporting Information). To provide a robust phylogenetic background to our diagnostic sequences, which are ~ 500 bp long, we selected sequences from GenBank that are over 1500 bp long.

Our aim was to select 10–10 sequences from the five genotypes reported in *T. equi* (Tirosh-Levy et al. 2020). However, only two 1500-bp-long sequences (AB515310 and EU642507) were available for genotype B from horses. Thus, we supplemented our selection with four sequences (KF597073, KF597077, KF597078 and KF597081) from waterbuck samples, raising the total number of sequences for genotype B to six. In addition to the *T. equi* samples, we used five *Theileria parva* sequences (HQ895973–HQ895975; HQ895984–HQ895985) as outgroups. Five *T. haneyi* sequences (KU647704–KU647708) were used to confirm the presence of *T. haneyi* in our sequences. A total of 80 terminals were analysed in the analysis. The full list of GenBank accession numbers for the sequences, together with their distribution data and assigned clade (by Tirosh-Levy et al. 2020), can be found in the Supporting Information. The sequences were aligned using MAFFT (Katoh and Toh 2008) and curated in BGME (Criscuolo and Gribaldo 2010). The substitution model was selected using SMS (Lefort et al. 2017) and the tree was analysed using maximum likelihood in PhyML (Guindon et al. 2010) on the NGPhylogeny server (Lemoine et al. 2019). Bootstrap values were calculated according to the method of Lemoine et al. (2018), and the tree was visualised and edited in iTol (Letunic and Bork 2024).

### Statistical analysis

The Fisher exact test (Agresti 2002) was applied as independence test on frequency tables within R-environment (v4.5.2, R Core Team 2025).

## Results

### The occurrence of horse piroplasmosis

**Fig. 1:**
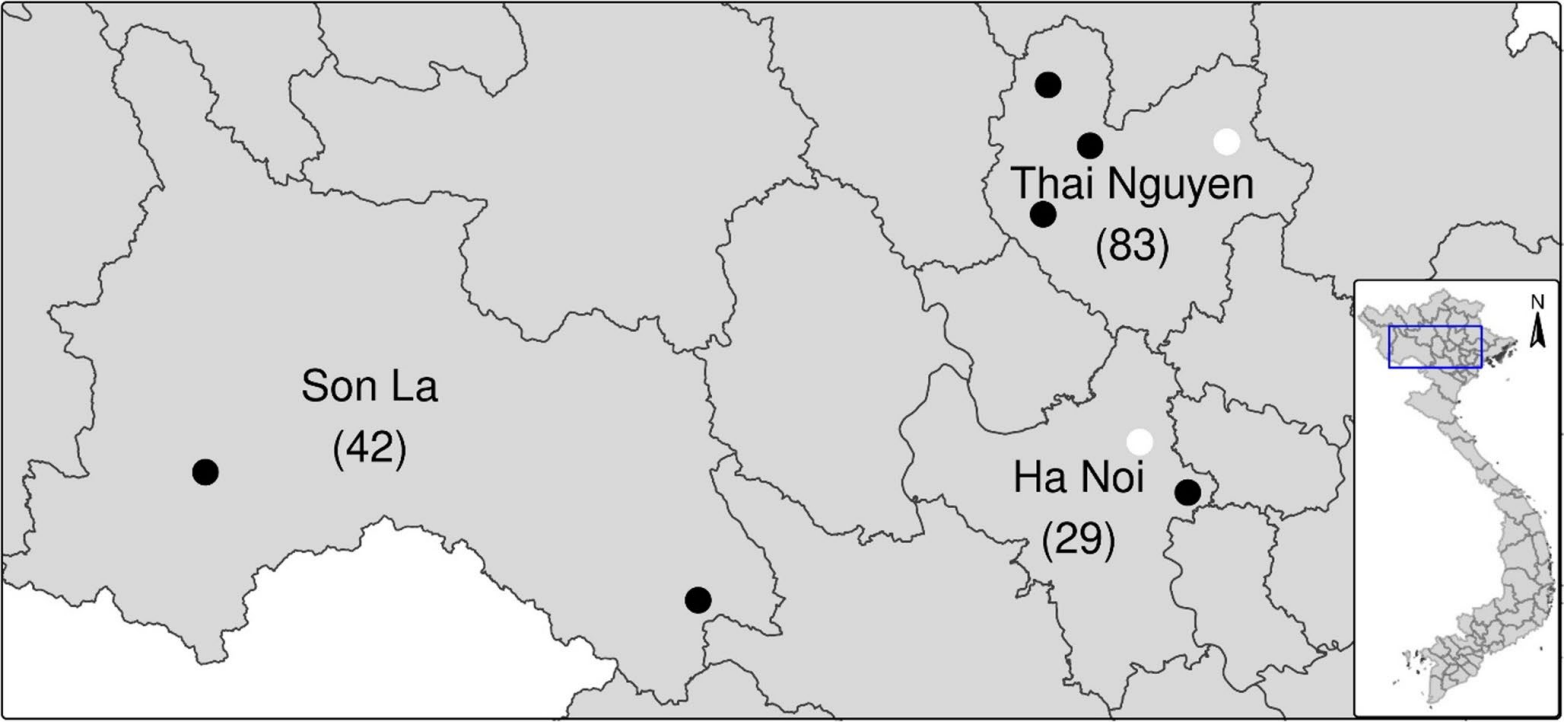
The map of Vietnam illustrates the three provinces with the number of samples. Black dots indicate districts where *Theileria* infected horses were detected, while white dots indicate districts where they were not

Targeting the 18S rRNA gene with specific PCR on 154 examined horses, 24 were found to be positive in six districts of the three provinces (Fig. 1).

The overall prevalence was 15.58% (95%CI: 10.70–22.14.70.14) (Table 1). Based on the sequence analysis, 21 samples were identified as *T. equi* and three as *T. haneyi*. *Theileria equi* was detected in six districts across three provinces (Fig. 1). The prevalence of infection was very similar in Hanoi and Son La provinces. *Babesia caballi* did not occur. There were no significant differences in the frequency of PCR-positive animals among the provinces (*p* = 0.4684).

**Table 1:** Prevalence of *Theileria equi* infection in horses, as detected by molecular methods in three provinces of Vietnam

Province	Number examined	Number infected	Prevalence (%)	95%CI
Ha Noi	29	6	20.69	9.85–38.39
Son La	42	9	21.43	11.71–35.94
Thai Nguyen	83	9	10.84	5.81–19.34
Overall	154	24	15.58	10.70–22.14

Males had slightly higher odds of *T. equi* infection than females, but this association was not significant (OR: 1.02, 95%CI: 0.38–2.67, *p* = 1). There was no significant association between age group (1–3 years old: positive = 7, negative = 48; 3–6 years old: positive = 14, negative = 57; over six years old: positive = 3, negative = 25) and number of horses infected with *T. equi* (*p* = 0.4684).

### Sequences analysis of T. equi

**Fig. 2:**
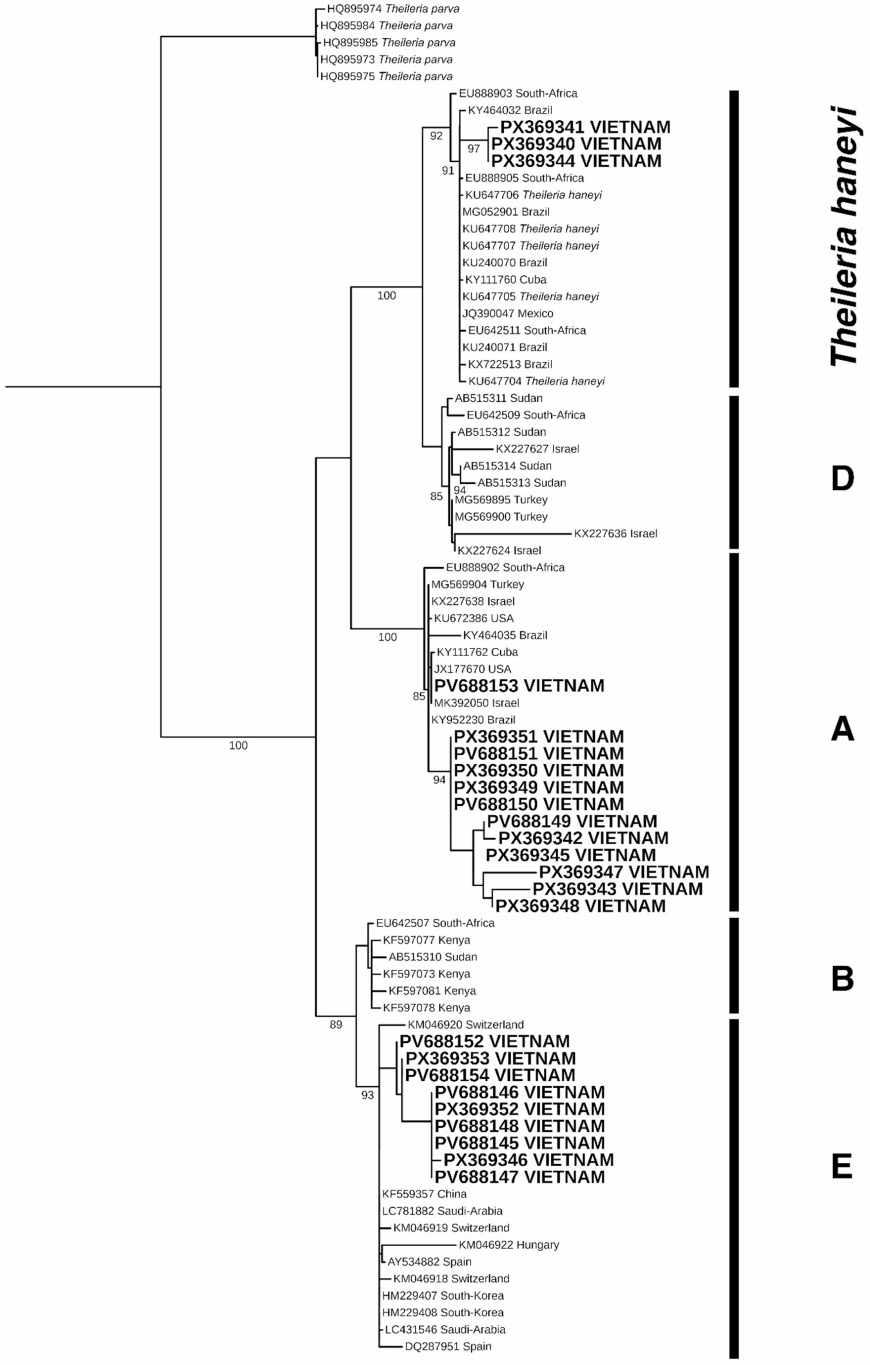
The inferred Maximum likelihood tree of *T. equi *18S rRNA sequences. The tree included 80 sequences and 1548 positions. TN93 + G + I substitution models were used, bootstrap values below 85 are not shown. Five *Theileria parva* sequences were used as outgroups, five *Theileria haneyi* sequences were used to identify the species’ clade

Based on the NCBI BLAST results, the newly obtained *T. equi* 18S rRNA sequences (PV688145–PV688154 and PX369340–PX369353) shared 99.09–100% identity to each other and to *T. equi* sequences in GenBank. The results of the maximum likelihood analysis are shown in Fig. 2. Our results were consistent with those published by Tirosh-Levy et al. (2020), although the placement of genotype A with B + E was not supported. Twelve samples (PV688149–PV688151, PV688153, PX369342–PX369343, PX369345, PX369347–PX369351) formed two distinct groups within genotype A; three samples (PX369340, PX369341, PX369344) formed a distinct group within genotype C; and the remaining nine samples (PV688145–PV688148, PV688152, PV688154, PX369353, PX369352, PX369346) formed a distinct group within genotype E (Fig. 2). Genotypes A and E were detected in 12 and nine horses living in three provinces, respectively. Genotype C was found in two horses in Hanoi province and one horse in Thai Nguyen province. Genotype C was found in two horses in the province Hanoi and one horse in the province Thai Nguyen.

## Discussion

Since the beginning of the 20th century when Laveran (1901) gave the name *Piroplasma equi* to the intraerythrocytic parasite found in the blood of horses, equine piroplasmosis has been reported from many European, American and African countries (Onyiche et al. 2019; Tirosh-Levy et al. 2020). While it was hypothesised over a hundred years ago that piroplasmosis had occurred in a herd of mules in French Indochina (Schein 1917), there was no information available for many decades regarding the occurrence of this economically significant parasitic disease in Asia. However, since the 2010 s serological and/or molecular studies have revealed the presence of equine piroplasmosis in various Asian countries, with one or both haemoparasite species being detected (Munkhjargal et al. 2013; Kamyingkird et al. 2014; Nugraha et al. 2018; Ybañez et al. 2018; Wang et al. 2019; Zhao et al. 2020; Kumar et al. 2020; Khaing et al. 2025). To the best of our knowledge, no serological or molecular examinations of this tick-borne disease had been carried out in Vietnam prior to this survey.

The results revealed that 24 out of 154 blood samples collected in Vietnam contained the DNA of *T. equi*, which is the predominant species in some Asian countries (Tirosh-Levy et al. 2020; Kumar et al. 2020). Conversely, a higher prevalence of *B. caballi* than *T. equi* was reported in Mongolia (Munkhjargal et al. 2013). Neither *B. caballi* nor dual infections with the two protozoan species were identified in Vietnam. There are two possible explanations for the absence of *B. caballi*. Either this species does not occur among horses in the provinces studied, or it was present but efficiently eliminated by the host immune system. Another possibility is that using a single-round PCR rather than a nested PCR did not allow *B. caballi* to be detected, given that its parasitemia is generally extremely low. This is contrasts with the lifelong persistence of *T. equi* in untreated horses (Brüning 1996).

When we compared our results (15.58%) with those of other studies in Asia, we found that a higher prevalence was reported in China (Wang et al. 2019; Zhao et al. 2020), Iran (Kalantari et al. 2022) and the Philippines (Ybañez et al. 2018), while a lower prevalence was found in India (Kumar et al. 2020), and Myanmar (Khaing et al. 2025). This variation in equine theileriosis prevalence across countries may be attributed to differences in competent vector occurrence and abundance, tick environmental factors, the application of various methods, and the efficacy of tick control measures (Onyiche et al. 2019). No significant correlation was found between the PCR results and the age of the horses. However, Ruegg et al. (2007) reported that a positive correlation was found between host age and *T. equi* infection in the surveyed provinces. As in a previous study (Steinman et al. 2012; gender was not found to be a risk factor for *T. equi* infection in this study. However, other authors (Ruegg et al. 2007; Moretti et al. 2010) did find a correlation between gender and infection. No horses were found to be infested with ticks during the sampling period. Therefore, we could not obtain any relevant data on tick species that could carry *T. equi*. However, most of the suspected tick genera (*Hyalomma*, *Dermacentor*, and *Haemaphysalis*), that may act as vectors (De Waal 1992; Scoles and Ueti 2015), are present in Vietnam (Hornok et al. 2024; Ngoc et al. 2025) as are stable flies, one of the most important mechanical vectors of *Theileria* spp. including *T. equi* (Hornok et al. 2020) which are commonly found on pastures. Furthermore, iatrogenic transmission cannot be ruled out, because contaminated needles and other equipment have been reported by several authors (Rothschild 2013; Wise et al. 2013) as a source of such infections. 

Preliminary studies have revealed five 18S rRNA genotypes of *T. equi* (A, B, C, D and E) (Bhoora et al. 2009; Qablan et al. 2012; Onyiche et al. 2019). The genetic diversity of *T. equi* may be important for the transmission of this pathogen (Manna et al. 2018; Tirosh-Levy et al. 2020). However, more molecular epidemiological data are needed to confirm this. This study provides the first data on the genetic diversity of *T. equi* in Vietnam. Three genotypes (A, C and E) were identified through sequence analysis of *T. equi* samples taken from 24 horses. The most prevalent genotype was A, which has been reported in many countries and is more commonly associated with clinical theileriosis than other genotypes (Tirosh-Levy et al. 2021). Genotype E was found in nine animals in the present study and has primarily been reported in China (Chen et al. 2022) and Mongolia (Otgonsuren et al. 2024). Genotype C was found in three horses. A comparison of the phylogenetic tree from the present study with that from Knowles et al. (2018) suggests that a genetically homogeneous group within genotype C most likely corresponds to *T. haneyi*. This is corroborated by sequences EU642511 and EU888905 from South Africa, as well as the *T. haneyi* reference sequences KU647704–KU647708 included in our study (Fig. 2). We also included EU888903, which Knowles et al. (2018) identified as *T. equi*, thus marking the species limits. As *T. equi* has been assigned to genotype C rather than *T. haneyi* (Tirosh-Levy et al. 2021), the taxonomy of *T. haneyi* remains uncertain. If it is accepted as valid, then our records will be the first from Vietnam.

The time and place of the arrival of *T. equi*, the agent of theileriosis in horses, in Vietnam is unknown. However, based on reports by Laveran (1901) and Schein (1917), it is evident that this haemoparasite species was present in northern Vietnam at the beginning of the 20th century, resulting in clinical symptoms and death in mules in the Haiphong area.

## Conclusion

In conclusion, the results of the first molecular survey suggest that *T. equi*, the agent that causes equine piroplasmosis, is present in northern Vietnam, albeit in a subclinical form. Further studies are needed to assess the risk posed by *T. equi* and *B. caballi* in other parts of the country. These studies should be accompanied by tick vector surveillance and educational programmes for veterinarians about this notifiable disease.

## Supplementary Information

Below is the link to the electronic supplementary material.

Supplementary File 1 (JPG 105 KB)

Supplementary File 2 (PDF 306 KB)

## Data Availability

The sequences obtained during this study have been deposited in GenBank under the following accession numbers: 18 S rRNA gene: PV688145-688153 and PX369342-369353. All other relevant data are included in the manuscript, and the Supporting Information is available.

## References

[CR1] Agresti A (2002) Categorical data analysis. Second edition. New York: Wiley. Pages 91–101

[CR2] Allsopp MT, Lewis BD, Penzhorn BL (2007) Molecular evidence for transplacental transmission of *Theileria equi* from carrier mares to their apparently healthy foals. Vet Parasitol 148:130–136. 10.1016/j.vetpar.2007.05.017 17601669 10.1016/j.vetpar.2007.05.017

[CR3] Bhoora R, Franssen L et al (2009) Sequence heterogeneity in the 18S rRNA gene within *Theileria equi* and *Babesia caballi* from horses in South Africa. Vet Parasitol 159:112–120. 10.1016/j.vetpar.2008.10.004 19019541 10.1016/j.vetpar.2008.10.004

[CR4] Brüning A (1996) Equine piroplasmosis an update on diagnosis, treatment and prevention. Br Vet J 152:139–151. 10.1111/j.2042-3292.1996.tb01850.x 8680838 10.1016/s0007-1935(96)80070-4

[CR5] Casati S, Sager H et al (2006) Presence of potentially pathogenic *Babesia* sp. for human in *Ixodes ricinus* in Switzerland. Ann Agric Environ Med 13:65–70 16841874

[CR6] Chauvin A, Moreau E et al (2009) Babesia and its hosts: adaptation to long-lasting interactions as a way to achieve efficient transmission. Vet Res 40(2):37. 10.1051/vetres/2009020 19379662 10.1051/vetres/2009020 PMC2695028

[CR7] Chen K, Hu Z et al (2022) Development of a duplex real-time PCR assay for simultaneous detection and differentiation of *Theileria equi* and *Babesia Caballi*. Transbound Emerg Dis 69:e1338–1349. 10.3389/fvets.2022.873190 35089645 10.1111/tbed.14464

[CR8] Chhabra S, Ranjan R et al (2012) Transplacental transmission of *Babesia equi* (*Theileria equi*) from carrier mares to foals. J Parasit Dis 36:31–33. 10.1007/s12639-011-0072-1 23543072 10.1007/s12639-011-0072-1 PMC3284609

[CR34] R Core Team (2025) R: A Language and Environment for Statistical Computing. R Foundation for Statistical Computing, Vienna, Austria. https://www.R-project.org

[CR9] Criscuolo A, Gribaldo S (2010) Bmge (block mapping and gathering with entropy): a new software for selection of phylogenetic informative regions from multiple sequence alignments. BMC Evol Biol 10(1):210. 10.1186/1471-2148-10-210 20626897 10.1186/1471-2148-10-210 PMC3017758

[CR10] De Waal DT (1992) Equine piroplasmosis: a review. Br Vet J 148(1):6–14. 10.1016/0007-1935(92)90061-5 1551016 10.1016/0007-1935(92)90061-5

[CR11] Githaka N, Konnai S et al (2014) Identification and sequence characterization of novel *Theileria* genotypes from the waterbuck (*Kobus defassa*) in a *Theileria parva*-endemic area in Kenya. Vet Parasitol 202(3–4):180–193. 10.1016/j.vetpar.2014.02.056 24690249 10.1016/j.vetpar.2014.02.056

[CR12] Guindon S, Dufayard JF et al (2010) New algorithms and methods to estimate maximum-likelihood phylogenies: assessing the performance of PhyML 3.0. Syst Biol 59(3):307–321. 10.1093/sysbio/syq010 20525638 10.1093/sysbio/syq010

[CR14] Hornok S, Takács N et al (2020) DNA of *Theileria orientalis*, *T. equi* and *T. capreoli* in stable flies (*Stomoxys calcitrans*). Parasit Vectors 13:186. 10.1186/s13071-020-04041-1 32272968 10.1186/s13071-020-04041-1 PMC7144340

[CR13] Hornok S, Farkas R et al (2024) A morpho-phylogenetic update on ixodid ticks infesting cattle and buffalos in Vietnam, with three new species to the fauna and a checklist of all species indigenous to the country. Parasit Vectors 17:319. 10.1186/s13071-024-06384-5 39061114 10.1186/s13071-024-06384-5 PMC11282669

[CR15] Kalantari M, Sharifiyazdi H et al (2022) *Theileria equi* in the horses of Iran: molecular detection, genetic diversity, and hematological findings. Vet Parasitol Reg Stud Rep 36:100792. 10.1016/j.vprsr.2022.100792 10.1016/j.vprsr.2022.100792 36436901

[CR16] Kamyingkird K, Yangtara S et al (2014) Seroprevalence of *Babesia Caballi* and *Theileria equi* in horses and mules from Northern Thailand. J Protozool Res 17:11–17. 10.1016/j.tvjl.2014.09.025

[CR17] Katoh K, Toh H (2008) Recent developments in the MAFFT multiple sequence alignment program. Brief Bioinform 9(4):286–298. 10.1093/molbev/mst010 18372315 10.1093/bib/bbn013

[CR18] Khaing Y, Htun LL et al (2025) Microscopic examination of haemoparasites and the first molecular detection of *Theileria equi* in horses in Myanmar. Parasitol Res 124:42. 10.1007/s00436-025-08488-y 40257584 10.1007/s00436-025-08488-y PMC12011657

[CR19] Knowles DP, Kappmeyer LS et al (2018) Discovery of a novel species, *Theileria haneyi* n. sp., infective to equids, highlights exceptional genomic diversity within the genus *Theileria*: implications for apicomplexan parasite surveillance. Int J Parasitol 48:679–690. 10.1016/j.ijpara.2018.03.010 29885436 10.1016/j.ijpara.2018.03.010

[CR20] Kumar S, Sudan V et al (2020) *Babesia* (*Theileria*) *equi* genotype A among Indian equine population. Vet Parasitol Reg Stud Rep 19:100367. 10.1016/j.vprsr.2019.100367 10.1016/j.vprsr.2019.100367 32057394

[CR21] Laveran M (1901) Contribution a l’etude de *Piroplasma equi*. CR Soc Biol 53:385–388

[CR22] Lefort V, Longueville JE, Gascuel O (2017) SMS: smart model selection in PhyML. Mol Biol Evol 34(9):2422–2424. 10.1093/molbev/msx149 28472384 10.1093/molbev/msx149 PMC5850602

[CR23] Lemoine F, Correia D et al (2019) NGPhylogeny. Fr: new generation phylogenetic services for non-specialists. Nucleic Acids Res 47(W1):W260–265. 10.1093/nar/gkz303 31028399 10.1093/nar/gkz303 PMC6602494

[CR24] Letunic I, Bork P (2024) Interactive tree of life (iTOL) v6: recent updates to the phylogenetic tree display and annotation tool. Nucleic Acids Res 52(W1):78–82. 10.1093/nar/gkae268 10.1093/nar/gkae268 PMC11223838 38613393

[CR25] Manna G, Cersini A, Nardini R (2018) Genetic diversity of *Theileria equi* and *Babesia caballi* infecting horses of Central-Southern Italy and preliminary results of its correlation with clinical and serological status. Ticks Tick-borne Dis 9:1212–1220. 10.1016/j.ttbdis.2018.05.005 29752142 10.1016/j.ttbdis.2018.05.005

[CR26] Mendoza FJ, Pérez-Écija A et al (2024) New insights in the diagnosis and treatment of equine piroplasmosis: pitfalls, idiosyncrasies, and myths. Front Vet Sci 11:1459989. 10.3389/fvets.2024.1459989 39205808 10.3389/fvets.2024.1459989 PMC11349644

[CR27] Moretti A, Mangili V, Salvatori R (2010) Prevalence and diagnosis of Babesia and Theileria infections in horses in Italy: a preliminary study. Vet J 184:346–350. 10.1016/j.tvjl.2009.03.021 19394253 10.1016/j.tvjl.2009.03.021

[CR28] Munkhjargal T, Sivakumar T et al (2013) Prevalence and genetic diversity of equine piroplasms in Tov province, Mongolia. Infect Genet Evol 16:178–185. 10.1016/j.meegid.2013.02.005 23416256 10.1016/j.meegid.2013.02.005

[CR29] Ngoc DP, Ha TDT et al (2025) The current status and predicted climate-driven range expansion of *Rhipicephalus microplus* in northern Vietnam. Acta Trop 268:107732. 10.1016/j.actatropica.2025.107732 40617273 10.1016/j.actatropica.2025.107732

[CR30] Nugraha AB, Cahyaningsih U et al (2018) Serological and molecular prevalence of equine piroplasmosis in Western Java, Indonesia. Vet Parasitol Reg Stud Rep 14:1–6. 10.1016/j.vprsr.2018.07.009 10.1016/j.vprsr.2018.07.009 31014711

[CR31] Onyiche TGE, Suganuma K et al (2019) A review on equine piroplasmosis: epidemiology, vector ecology, risk factors, host immunity, diagnosis and control. Int J Environ Res Public Health 16:1736. 10.3390/ijerph16101736 31100920 10.3390/ijerph16101736 PMC6572709

[CR32] Otgonsuren D, Amgalanbaatar T, Narantsatsral S (2024) Epidemiology and genetic diversity of *Theileria equi* and *Babesia caballi* in Mongolian horses. Infect Genet Evol 119:105571. 10.1016/j.meegid.2024.105571 38365128 10.1016/j.meegid.2024.105571

[CR33] Qablan MA, Sloboda M et al (2012) Quest for the piroplasms in camels: identification of *Theileria equi* and *Babesia caballi* in Jordanian dromedaries by PCR. Vet Parasitol 186:456–460. 10.1016/j.vetpar.2011.11.070 22186193 10.1016/j.vetpar.2011.11.070

[CR35] Rothschild CM (2013) Equine piroplasmosis. J Equine Vet Sci 33:497–508. 10.1016/j.jevs.2013.03.189

[CR36] Ruegg SR, Torgerson P et al (2007) Age-dependent dynamics of *Theileria equi* and *Babesia caballi* infections in southwest Mongolia based on IFAT and/or PCR prevalence data from domestic horses and ticks. Parasitology 134:939–947. 10.1017/S0031182007002405 17306055 10.1017/S0031182007002405

[CR37] Salim B, Alanazi AD et al (2019) Potential role of dogs as sentinels and reservoirs for piroplasms infecting equine and cattle in Riyadh City, Saudi Arabia. Acta Trop 193:7883. 10.1016/j.actatropica.2019.02.029 10.1016/j.actatropica.2019.02.029 30831114

[CR38] Schein H (1917) Equine piroplasmosis in South Annam, French Indo-China. Bull Soc Exot Pathol 10:871–873

[CR39] Scoles GA, Ueti MW (2015) Vector ecology of equine piroplasmosis. Annu Rev Entomol 60:561–580. 10.1146/annurev-ento-010814-021110 25564746 10.1146/annurev-ento-010814-021110

[CR40] Steinman A, Zimmerman T et al (2012) Demographic and environmental risk factors for infection by *Theileria equi* in 590 horses in Israel. Vet Parasitol 187:558–562. 10.1016/j.vetpar.2012.01.018 22293151 10.1016/j.vetpar.2012.01.018

[CR41] Tamzali Y (2013) Equine piroplasmosis: an updated review. Equine Vet Educ 25:590–598. 10.1111/eve.12070

[CR42] Tirosh-Levy S, Gottlieb Y et al (2020) Twenty years of equine piroplasmosis research: global distribution, molecular diagnosis, and phylogeny. Pathogens 9(11):926. 10.3390/pathogens9110926 33171698 10.3390/pathogens9110926 PMC7695325

[CR43] Tirosh-Levy S, Mazuz ML, Savitsky I (2021) A serological and molecular prevalence of *Babesia caballi* in apparently healthy horses in Israel. Pathogens 10:445. 10.3390/pathogens10040445 33917822 10.3390/pathogens10040445 PMC8068206

[CR44] Wang J, Liua J et al (2019) The first molecular detection and genetic diversity of *Babesia Caballi* and *Theileria equi* in horses of Gansu province, China. Ticks Tick Born Dis 10:528–532. 10.1016/j.ttbdis.2019.01.003 10.1016/j.ttbdis.2019.01.003 30670354

[CR45] Wise LN, Kappmeyer LS et al (2013) Review of equine piroplasmosis. J Vet Intern Med 27:1334–1346. 10.1111/jvim.12168 24033559 10.1111/jvim.12168

[CR46] Ybañez AP, Ybañez RHD et al (2018) Serological and molecular detection of *Theileria equi* and *Babesia caballi* in Philippine horses. Ticks Tick-borne Dis 9:1125–1128. 10.1016/j.ttbdis.2018.04.010 29693550 10.1016/j.ttbdis.2018.04.010

[CR47] Zhao S, Wang H et al (2020) First report of genetic diversity and risk factor analysis of equine piroplasm infection in equids in Jilin, China. Parasit Vectors 13:459. 10.1186/s13071-020-04338-1 32907616 10.1186/s13071-020-04338-1 PMC7479743

