# Supplementary figures and images for: The first molecular detection of equine piroplasmosis in Vietnam and genetic characterization of three co-circulating genotypes of Theileria equi
Source: Parasitol Res. 2026 Feb 5;125(1):14. doi: 10.1007/s00436-026-08630-4 (PMC12876454; doi:10.1007/s00436-026-08630-4)

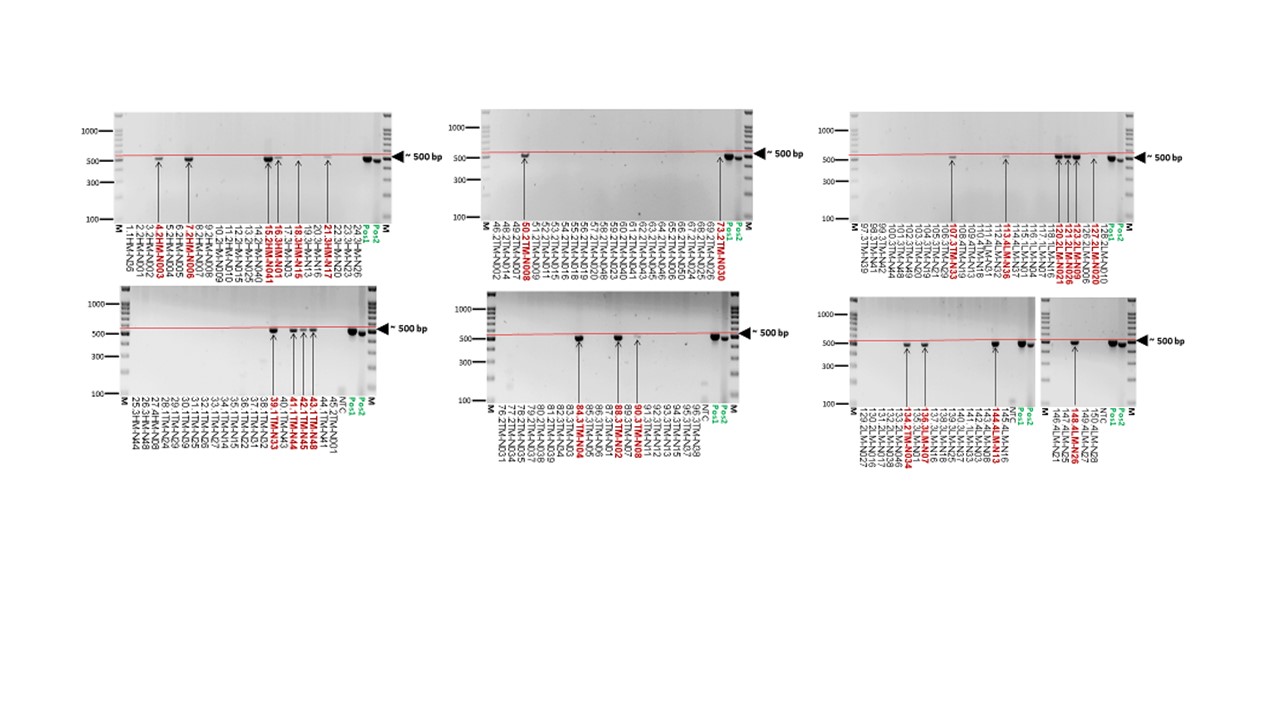

Supplement: Supplementary file 1 — (JPG 105 KB) [file 436_2026_8630_MOESM1_ESM.jpg]
