# Supplementary material for: The first molecular detection of equine piroplasmosis in Vietnam and genetic characterization of three co-circulating genotypes of Theileria equi
Source: Parasitol Res. 2026 Feb 5;125(1):14. doi: 10.1007/s00436-026-08630-4 (PMC12876454; doi:10.1007/s00436-026-08630-4)
Supplement: Supplementary file 2 — (PDF 306 KB) [file 436_2026_8630_MOESM2_ESM.pdf]

SUPPORTING INFORMATION

USED SEQUENCES CLADE ASSORTMENTS 2
FINAL ALIGNMENT 4
MODEL SELECTION 45
ML ANALYSIS 47
ML TREE 50
QUESTIONNAIRE FOR HORSES 53

USED SEQUENCES CLADE ASSORTMENTS

GenBank accession	Ditribution	Clade	GenBank accession	Ditribution	Clade
EU888902	South Africa	A	MG569900	Turkey	D
JX177670	USA	A	AY534882	Spain	E
KU672386	USA	A	DQ287951	Spain	E
KX227638	Israel	A	HM229407	South Korea	E
KY111762	Cuba	A	HM229408	South Korea	E
KY464035	Brazil	A	KF559357	China	E
KY952230	Brazil	A	KM046918	Switzerland	E
AY150062	Spain	A	KM046919	Switzerland	E
MG569904	Turkey	A	KM046920	Switzerland	E
MK392050	Israel	A	KM046922	Hungary	E
AB515310	Sudan	B	LC431546	Saudi Arabia	E
EU642507	South Africa	B	**PV688145**	**VIETNAM**	**E**
KF597073	Kenya	B	**PV688146**	**VIETNAM**	**E**
KF597077	Kenya	B	**PV688147**	**VIETNAM**	**E**
KF597078	Kenya	B	**PV688148**	**VIETNAM**	**E**
KF597081	Kenya	B	**PV688152**	**VIETNAM**	**E**
EU642511	South Africa	C	**PV688154**	**VIETNAM**	**E**
EU888903	South Africa	C	**PV688149**	**VIETNAM**	**A**
JQ390047	Mexico	C	**PV688150**	**VIETNAM**	**A**
EU888905	South Africa	C	**PV688151**	**VIETNAM**	**A**
KU240070	Brazil	C	**PV688153**	**VIETNAM**	**A**
KU240071	Brazil	C	**PX369340**	**VIETNAM**	**C**
KX722513	Brazil	C	**PX369341**	**VIETNAM**	**C**
KY111760	Cuba	C	**PX369342**	**VIETNAM**	**A**
KY464032	Brazil	C	**PX369343**	**VIETNAM**	**A**
MG052901	Brazil	C	**PX369344**	**VIETNAM**	**C**
AB515311	Sudan	D	**PX369345**	**VIETNAM**	**A**
AB515312	Sudan	D	**PX369346**	**VIETNAM**	**E**
AB515313	Sudan	D	**PX369347**	**VIETNAM**	**A**
AB515314	Sudan	D	**PX369348**	**VIETNAM**	**A**
EU642509	South Africa	D	**PX369349**	**VIETNAM**	**A**
KX227624	Israel	D	**PX369350**	**VIETNAM**	**A**
KX227627	Israel	D	**PX369351**	**VIETNAM**	**A**
KX227636	Israel	D	**PX369352**	**VIETNAM**	**E**
MG569895	Turkey	D	**PX369353**	**VIETNAM**	**E**

SUPPORTING INFORMATION

USED SEQUENCES CLADE ASSORTMENTS 2
FINAL ALIGNMENT 4
MODEL SELECTION 45
ML ANALYSIS 47
ML TREE 50
QUESTIONNAIRE FOR HORSES 53

FINAL ALIGNMENT

>HQ895973_Theileria_parva

ATGGCTCATTACAACAGTTATAGTTTATTTGATGTTCGTTTTTACATGGATAACCGTGCTAATTGTAGGG
CTAATACATGTTCGAGGTTGGCGGCGTTTATTAGACCTAAAACCAAACCGCTTGCGGTG---
TCCGGTGATTCATAATAAATATGCGAATCGTA-----
CTTGGTGCGATGTATCATTCAAGTTTCTGACCTATCAGCTTTGGACGGTAGGGTATTGGCCTACCGG
GGCAACGACGGGTAACGGGGAATTAGGGTTCGATTCCGGAGAGGGAGCCTGAGAAACGGCTACC
ACATCTAAGGAAGGCAGCAGGCGCGCAAATTACCCAATCCTGACACAGGGAGGTAGTGACAAGAA
ATAACAATACGGGGCTTAAAGTCTTGTAATTGGAATGATGGGAATTTAAACCTCTTCCAGAGTATCAAT
TGGAGGGCAAGTCTGGTGCCAGCAGCCGCGGTAATTCCAGCTCCAATAGCGTATATTAAAATTGTT
GCAGTTAAAAAGCTCGTAGTTGAATTTCTGCTGCATCGCTGTGTCCCTT------
CGGGGTCTCTGCATGTGGCTTATTTCGGACGGAGTGCTTTGTCTGGATGTTTACTTTGAGAAAATTAG
AGTGCTCAAAGCAGGCTTTTGCCTTGAATAGTTTAGCATGGAATAATAAAGTAGGACTTTGGTTCTATT
TTGTTGGTTTTAGGTACCAAAGTAATGGTTAATAGGAACAGTTGGGGGCATTCGTATTTAACTGTCAGA
GGTGAAATTCTTAGATTTGTTAAAGACGAACTACTGCGAAAGCATTTGCCAAGGATGTTTTCATTAATC
AAGAACGAAAGTTAGGGGATCGAAGACGATCAGATACCGTCGTAGTCCTAACCATAAACAATGCCG
ACTAGAGATTGGAGGTCGTCAGTTTTTACGACTCCTTCAGCACCTTGAGAGAAATCAAAGTCTTTGGG
TTCTGGGGGGAGTATGGTCGCAAGGCTGAAACTTAAAGGAATTGACGGAAGGGCACCACCAGGCG
TGGAGCCTGCGGCTTAATTTGACTCAACACGGGGAAACTCACCAGGTCCAGACAAAGGAAGGATT
GACAGATTGATAGCTCTTTCTTGATTCTTTGGGTGGTGGTGCATGGCCGTTCTTAGTTGGTGGAGTGA
TTTGTCTGGTTAATTCCGTTAACGAACGAGACCTTAACCTGCTAAATAGGGTACGGGAATATGTCCCG
TCATCGCTTCTTAGAGGGACTTTGCGGTTATAAATCGCAAGGAAGTTTAAGGCAATAACAGGTCTGTG
ATGCCCTTAGATGTCCTGGGCTGCACGCGCGCTACACTGATGCGTTCATCGAGTTTATCCTTGGCC
GAGAGGCCCGGGTAATCTTTAGTACGCATCGTGATGGGGATCGATTATTGCAATTGTTAATCGTGAA
CGAGGAATGCCTAGTATGCGCAAGTCATCAGCTTGTGCAGATTACGTCCCTGCCCTTTGTACACAC
CGCCCGTCGCTCCTACCGATCGAGTGATCCG

>HQ895974_Theileria_parva

ATGGCTCATTACAACAGTTATAGTTTATTTGATGTTCGTTTTTACATGGATAACCGTGCTAATTGTAGGG
CTAATACATGTTCGAGGTTGGCGGCGTTTATTAGACCTAAAACCAAACCGCTTGCGGTG---
TCCGGTGATTCATAATAAATATGCGAATCGTA-----
CTTTGTACGATGTATCATTCAAGTTTCTGACCTATCAGCTTTGGACGGTAGGGTATTGGCCTACCGGG
GCAACGACGGGTAACGGGGAATTAGGGTTCGATTCCGGAGAGGGAGCCTGAGAAACGGCTACCA
CATCTAAGGAAGGCAGCAGGCGCGCAAATTACCCAATCCTGACACAGGGAGGTAGTGACAAGAAA
TAACAATACGGGGCTTAAAGTCTTGTAATTGGAATGATGGGAATTTAAACCTCTTCCAGAGTATCAATT
GGAGGGCAAGTCTGGTGCCAGCAGCCGCGGTAATTCCAGCTCCAATAGCGTATATTAAAATTGTTG
CAGTTAAAAAGCTCGTAGTTGAATTTCTGCTGCATCGCTGTGTCCCTT------
CGGGGTCTCTGCATGTGGCTTATTTCGGACGGAGTGCTTTGTCTGGATGTTTACTTTGAGAAAATTAG
AGTGCTCAAAGCAGGCTTTTGCCTTGAATAGTTTAGCATGGAATAATAAAGTAGGACTTTGGTTCTATT
TTGTTGGTTTTAGGTACCAAAGTAATGGTTAATAGGAACAGTTGGGGGCATTCGTATTTAACTGTCAGA
GGTGAAATTCTTAGATTTGTTAAAGACGAACTACTGCGAAAGCATTTGCCAAGGATGTTTTCATTAATC
AAGAACGAAAGTTAGGGGATCGAAGACGATCAGATACCGTCGTAGTCCTAACCATAAACAATGCCG
ACTAGAGATTGGAGGTCGTCAGTTTTTACGACTCCTTCAGCACCTTGAGAGAAATCAAAGTCTTTGGG
TTCTGGGGGGAGTATGGTCGCAAGGCTGAAACTTAAAGGAATTGACGGAAGGGCACCACCAGGCG

TGGAGCCTGCGGCTTAATTTGACTCAACACGGGGAAACTCACCAGGTCCAGACAAAGGAAGGATT
GACAGATTGATAGCTCTTTCTTGATTCTTTGGGTGGTGGTGCATGGCCGTTCTTAGTTGGTGGAGTGA
TTTGTCTGGTTAATTCCGTTAACGAACGAGACCTTAACCTGCTAAATAGGGTACGGGAATATGTCCCG
TCATCGCTTCTTAGAGGGACTTTGCGGTTATAAATCGCAAGGAAGTTTAAGGCAATAACAGGTCTGTG
ATGCCCTTAGATGTCCTGGGCTGCACGCGCGCTACACTGATGCGTTCATCGAGTTTGTCCTTGGCC
GAGAGGCCCGGGTAATCTTTAGTACGCATCGTGATGGGGATCGATTATTGCAATTGTTAATCGTGAA
CGAGGAATGCCTAGTATGCGCAAGTCATCAGCTTGTGCAGATTACGTCCCTGCCCTTTGTACACAC
CGCCCGTCGCTCCTACCGATCGAGTGATCCG

>HQ895975_Theileria_parva

ATGGCTCATTACAACAGTTATAGTTTATTTGATGTTCGTTTTTACATGGATAACCGTGCTAATTGTAGGG
CTAATACATGTTCGAGGTTGGCGGCGTTTATTAGACCTAAAACCAAACCGCTTGCGGTG---
TCCGGTGATTCATAATAAATATGCGAATCGTA-----
CTTGGTGCGATGTATCATTCAAGTTTCTGACCTATCAGCTTTGGACGGTAGGGTATTGGCCTACCGG
GGCAACGACGGGTAACGGGGAATTAGGGTTCGATTCCGGAGAGGGAGCCTGAGAAACGGCTACC
ACATCTAAGGAAGGCAGCAGGCGCGCAAATTACCCAATCCTGACACAGGGAGGTAGTGACAAGAA
ATAACAATACGGGGCTTAAAGTCTTGTAATTGGAATGATGGGAATTTAAACCTCTTCCAGAGTATCAAT
TGGAGGGCAAGTCTGGTGCCAGCAGCCGCGGTAATTCCAGCTCCAATAGCGTATATTAAAATTGTT
GCAGTTAAAAAGCTCGTAGTTGAATTTCTGCTGCATCGCTGTGTCCCTT------
CGGGGTCTCTGCATGTGGCTTATTTCGGACGGAGTGCTTTGTCTGGATGTTTACTTTGAGAAAATTAG
AGTGCTCAAAGCAGGCTTTTGCCTTGAATAGTTTAGCATGGAATAATAAAGTAGGACTTTGGTTCTATT
TTGTTGGTTTTAGGTACCAAAGTAATGGTTAATAGGAACAGTTGGGGGCATTCGTATTTAACTGTCAGA
GGTGAAATTCTTAGATTTGTTAAAGACGAACTACTGCGAAAGCATTTGCCAAGGATGTTTTCATTAATC
AAGAACGAAAGTTAGGGGATCGAAGACGATCAGATACCGTCGTAGTCCTAACCATAAACAATGCCG
ACTAGAGATTGGAGGTCGTCAGTTTTTACGACTCCTTCAGCACCTTGAGAGAAATCAAAGTCTTTGGG
TTCTGGGGGGAGTATGGTCGCAAGGCTGAAACTTAAAGGAATTGACGGAAGGGCACCACCAGGCG
TGGAGCCTGCGGCTTAATTTGACTCAACACGGGGAAACTCACCAGGTCCAGACAAAGGAAGGATT
GACAGATTGATAGCTCTTTCTTGATTCTTTGGGTGGTGGTGCATGGCCGTTCTTAGTTGGTGGAGTGA
TTTGTCTGGTTAATTCCGTTAACGAACGAGACCTTAACCTGCTAAATAGGGTACGGGAATATGTCCCG
TCATCGCTTCTTAGAGGGACTTTGCGGTTATAAATCGCAAGGAAGTTTAAGGCAATAACAGGTCTGTG
ATGCCCTTAGATGTCCTGGGCTGCACGCGCGCTACACTGATGCGTTCATCGAGTTTATCCTTGGCC
GAGAGGCCCGGGTAATCTTTAGTACGCATCGTGATGGGGATCGATTATTGCAATTGTTAATCGTGAA
CGAGGAATGCCTAGTATGCGCAAGTCATCAGCTTGTGCAGATTACGTCCCTGCCCTTTGTACACAC
CGCCCGTCGCTCCTACCGATCGAGTGATCCG

>HQ895984_Theileria_parva

ATGGCTCATTACAACAGTTATAGTTTATTTGATGTTCGTTTTTACATGGATAACCGTGCTAATTGTAGGG
CTAATACATGTTCGAGGTTGGCGGCGTTTATTAGACCTAAAACCAAACCGCTTGCGGTG---
TCCGGTGATTCATAATAAATATGCGAATCGTA-----
CTTAGTGCGATGTATCATTCAAGTTTCTGACCTATCAGCTTTGGACGGTAGGGTATTGGCCTACCGG
GGCAACGACGGGTAACGGGGAATTAGGGTTCGATTCCGGAGAGGGAGCCTGAGAAACGGCTACC
ACATCTAAGGAAGGCAGCAGGCGCGCAAATTACCCAATCCTGACACAGGGAGGTAGTGACAAGAA
ATAACAATACGGGGCTTAAAGTCTTGTAATTGGAATGATGGGAATTTAAACCTCTTCCAGAGTATCAAT
TGGAGGGCAAGTCTGGTGCCAGCAGCCGCGGTAATTCCAGCTCCAATAGCGTATATTAAAATTGTT
GCAGTTAAAAAGCTCGTAGTTGAATTTCTGCTGCATCGCTGTGTCCCTT------
CGGGGTCTCTGCATGTGGCTTATTTCGGACGGAGTGCTTTGTCTGGATGTTTACTTTGAGAAAATTAG
AGTGCTCAAAGCAGGCTTTTGCCTTGAATAGTTTAGCATGGAATAATAAAGTAGGACTTTGGTTCTATT
TTGTTGGTTTTAGGTACCAAAGTAATGGTTAATAGGAACAGTTGGGGGCATTCGTATTTAACTGTCAGA

GGTGAAATTCTTAGATTTGTTAAAGACGAACTACTGCGAAAGCATTTGCCAAGGATGTTTTCATTAATC
AAGAACGAAAGTTAGGGGATCGAAGACGATCAGATACCGTCGTAGTCCTAACCATAAACAATGCCG
ACTAGAGATTGGAGGTCGTCAGTTTTTACGACTCCTTCAGCACCTTGAGAGAAATCAAAGTCTTTGGG
TTCTGGGGGGAGTATGGTCGCAAGGCTGAAACTTAAAGGAATTGACGGAAGGGCACCACCAGGCG
TGGAGCCTGCGGCTTAATTTGACTCAACACGGGGAAACTCACCAGGTCCAGACAAAGGAAGGATT
GACAGATTGATAGCTCTTTCTTGATTCTTTGGGTGGTGGTGCATGGCCGTTCTTAGTTGGTGGAGTGA
TTTGTCTGGTTAATTCCGTTAACGAACGAGACCTTAACCTGCTAAATAGGGTACGGGAATATGTCCCG
TCATCGCTTCTTAGAGGGACTTTGCGGTTATAAATCGCAAGGAAGTTTAAGGCAATAACAGGTCTGTG
ATGCCCTTAGATGTCCTGGGCTGCACGCGCGCTACACTGATGCGTTCATCGAGTTTATCCTTGGCC
GAGAGGCCCGGGTAATCTTTAGTACGCATCGTGATGGGGATCGATTATTGCAATTGTTAATCGTGAA
CGAGGAATGCCTAGTATGCGCAAGTCATCAGCTTGTGCAGATTACGTCCCTGCCCTTTGTACACAC
CGCCCGTCGCTCCTACCGATCGAGTGATCCG

>HQ895985_Theileria_parva

ATGGCTCATTACAACAGTTATAGTTTATTTGATGTTCGTTTTTACATGGATAACCGTGCTAATTGTAGGG
CTAATACATGTTCGAGGTTGGCGGCGTTTATTAGACCTAAAACCAAACCGCTTGCGGTG---
TCCGGTGATTCATAATAAATATGCGAATCGTA-----
CTTGGTGCGATGTATCATTCAAGTTTCTGACCTATCAGCTTTGGACGGTAGGGTATTGGCCTACCGG
GGCAACGACGGGTAACGGGGAATTAGGGTTCGATTCCGGAGAGGGAGCCTGAGAAACGGCTACC
ACATCTAAGGAAGGCAGCAGGCGCGCAAATTACCCAATCCTGACACAGGGAGGTAGTGACAAGAA
ATAACAATACGGGGCTTAAAGTCTTGTAATTGGAATGATGGGAATTTAAACCTCTTCCAGAGTATCAAT
TGGAGGGCAAGTCTGGTGCCAGCAGCCGCGGTAATTCCAGCTCCAATAGCGTATATTAAAATTGTT
GCAGTTAAAAAGCTCGTAGTTGAATTTCTGCTGCATCGCTGTGTCCCTT------
CGGGGTCTCTGCATGTGGCTTATTTCGGACGGAGTGCTTTGTCTGGATGTTTACTTTGAGAAAATTAG
AGTGCTCAAAGCAGGCTTTTGCCTTGAATAGTTTAGCATGGAATAATAAAGTAGGACTTTGGTTCTATT
TTGTTGGTTTTAGGTACCAAAGTAATGGTTAATAGGAACAGTTGGGGGCATTCGTATTTAACTGTCAGA
GGTGAAATTCTTAGATTTGTTAAAGACGAACTACTGCGAAAGCATTTGCCAAGGATGTTTTCATTAATC
AAGAACGAAAGTTAGGGGATCGAAGACGATCAGATACCGTCGTAGTCCTAACCATAAACAATGCCG
ACTAGAGATTGGAGGTCGTCAGTTTTTACGACTCCTTCAGCACCTTGAGAGAAATCAAAGTCTTTGGG
TTCTGGGGGGAGTATGGTCGCAAGGCTGAAACTTAAAGGAATTGACGGAAGGGCACCACCAGGCG
TGGAGCCTGCGGCTTAATTTGACTCAACACGGGGAAACTCACCAGGTCCAGACAAAGGAAGGATT
GACAGATTGATAGCTCTTTCTTGATTCTTTGGGTGGTGGTGCATGGCCGTTCTTAGTTGGTGGAGTGA
TTTGTCTGGTTAATTCCGTTAACGAACGAGACCTTAACCTGCTAAATAGGGTACGGGAATATGTCCCG
TGATCGCTTCTTAGAGGGACTTTGCGGTTATAAATCGCAAGGAAGTTTAAGGCAATAACAGGTCTGTG
ATGCCCTTAGATGTCCTGGGCTGCACGCGCGCTACACTGATGCGTTCATCGAGTTTATCCTTGGCC
GAGAGGCCCGGGTAATCTTTAGTACGCATCGTGAT-
GGGATCGATTATTGCAATTGTTAATCGTGAACGAGGAATGCCTAGTATGCGCAAGTCATCAGCTTGT
GCAGATTACGTCCCTGCCCTTTGTACACACCGCCCGTCGCTCCTACCGATCGAGTGATCCG

>KU647708_Theileria_haneyi

CAGTTATAGTTTATTTGATGTTAGTTTCTACATGGATAACCGTGCTAATTGTAGGGCTAATACATGTTTG
CTGTCAGTTGCGTTTATTAGACCTAAAACCTCCCCGCTTTTGCGGTGTATCGGTGATTCATAATAAATT
AGCGAATCGCATGGCTTTGCTGGCGATGTATCATTCAAGTTTCTGACCTATCAGCTTTGGACGGTAG
GGTATTGGCCTACCGGGGCAACGACGGGTAACGGGGAATTAGGGTTCGATTCCGGAGAGGGAGC
CTGAGAAACGGCTACCACATCTAAGGAAGGCAGCAGGCGCGCAAATTACCCAATCCTGACACAG
GGAGGTAGTGACAAGAAATAACAATACGGGGCTTTAAGTCTTGTAATTGGAATGATGGGAATTTAAAC
CTCTTCCAGAGTATCAATTGGAGGGCAAGTCTGGTGCCAGCAGCCGCGGTAATTCCAGCTCCAATA

GCGTATATTAAACTTGTTGCAGTTAAAAAGCTCGTAGTTGAATTTCTGCTGTATCGTTATCTTCTGC-
TTGACAGTTTGGTATCGTTATGGCTT-GTTGGGTCAC---
TTTGTGTCCCGGCGTTTACTTTGAGAAAATTAGAGTGCTTCAAGCAGGCTTTTGCCTTGAATACTTTAG
CATGGAATAATGGAGTAGGACTTTGGTTCTATTTTGTTGGTTTTAGGAGCCAGAGTAATGGTTAATAGG
AACAGTTGGGGGCATTCGTATTTGACTGTCAGAGGTGAAATTCTTAGATTTGTCAAAGACGAACTACT
GCGAAAGCATTTGCCAAGGATGTTTTCATTAATCAAGAACGAAAGTTAGGGGATCGAAGACGATCAG
ATACCGTCGTAGTCCTAACTATAAACGATGCCGACTAGAGATTGGAGGTCGTCAGTTTGAACGACTC
CTTCAGCACCTTGAGAGAAATCAAAGTCTTTGGGTTCTGGGGGGAGTATGGTCGCAAGGCTGAAAC
TTAAAGGAATTGACGGAAGGGCACCACCAGGCGTGGAGCCTGCGGCTTAATTTGACTCAACACGG
GGAAACTCACCAGGTCCAGACAGAGGAAGGATTGACAGATTGATGGCTCTTTCTTGATTCTTTGGGT
GGTGGTGCATGGCCGTTCTTAGTTGGTGGAGTGATTTGTCTGGTTAATTCCGTTAACGAACGAGACC
TTAACCTGCTAAATAGGGTGTTGGAGTTATGTTCTACACTGCTTCTTAGAGGGACTTTGCGGTCATAAA
TCGCAAGGAAGTTTAAGGCAATAACAGGTCTGTGATGCCCTTAGATGTCCTGGGCTGCACGCGCG
CTACACTGATGCATTCACTGAGTGTATCCTTGGCTGAGAGGCTTGGGTAATCTTGAGTATGCATCGT
GATGGGGATTGATTATTGTAATTCTTAATCATGAACGAGGAATGCCTAGTATGCGCGAGTCATCAGCT
CGTGCAGACTACGTCCCTGCCCT--

>KU647706_Theileria_haneyi

CAGTTATAGTTTATTTGATGTTAGTTTCTACATGGATAACCGTGCTAATTGTAGGGCTAATACATGTTTG
CTGTCAGTTGCGTTTATTAGACCTAAAACCTCCCCGCTTTTGCGGTGTATCGGTGATTCATAATAAATT
AGCGAATCGCATGGCTTTGCTGGCGATGTATCATTCAAGTTTCTGACCTATCAGCTTTGGACGGTAG
GGTATTGGCCTACCGGGGCAACGACGGGTAACGGGGAATTAGGGTTCGATTCCGGAGAGGGAGC
CTGAGAAACGGCTACCACATCTAAGGAAGGCAGCAGGCGCGCAAATTACCCAATCCTGACACAG
GGAGGTAGTGACAAGAAATAACAATACGGGGCTTTAAGTCTTGTAATTGGAATGATGGGAATTTAAAC
CTCTTCCAGAGTATCAATTGGAGGGCAAGTCTGGTGCCAGCAGCCGCGGTAATTCCAGCTCCAATA
GCGTATATTAAACTTGTTGCAGTTAAAAAGCTCGTAGTTGAATTTCTGCTGTATCGTTATCTTCTGC-
TTGACAGTTTGGTATCGTTATGGCTT-GTTGGGTCAC---
TTTGTGTCCCGGCGTTTACTTTGAGAAAATTAGAGTGCTTCAAGCAGGCTTTTGCCTTGAATACTTTAG
CATGGAATAATGGAGTAGGACTTTGGTTCTATTTTGTTGGTTTTAGGAGCCAGAGTAATGGTTAATAGG
AACAGTTGGGGGCATTCGTATTTGACTGTCAGAGGTGAAATTCTTAGATTTGTCAAAGACGAACTACT
GCGAAAGCATTTGCCAAGGATGTTTTCATTAATCAAGAACGAAAGTTAGGGGATCGAAGACGATCAG
ATACCGTCGTAGTCCTAACTATAAACGATGCCGTCTAGAGATTGGAGGTCGTCAGTTTGAACGACTC
CTTCAGCACCTTGAGAGAAATCAAAGTCTTTGGGTTCTGGGGGGAGTATGGTCGCAAGGCTGAAAC
TTAAAGGAATTGACGGAAGGGCACCACCAGGCGTGGAGCCTGCGGCTTAATTTGACTCAACACGG
GGAAACTCACCAGGTCCAGACAGAGGAAGGATTGACAGATTGATGGCTCTTTCTTGATTCTTTGGGT
GGTGGTGCATGGCCGTTCTTAGTTGGTGGAGTGATTTGTCTGGTTAATTCCGTTAACGAACGAGACC
TTAACCTGCTAAATAGGGTGTTGGAGTTATGTTCTACACTGCTTCTTAGAGGGACTTTGCGGTCATAAA
TCGCAAGGAAGTTTAAGGCAATAACAGGTCTGTGATGCCCTTAGATGTCCTGGGCTGCACGCGCG
CTACACTGATGCATTCACTGAGTGTATCCTTGGCTGAGAGGCTTGGGTAATCTTGAGTATGCATCGT
GATGGGGATTGATTATTGTAATTCTTAATCATGAACGAGGAATGCCTAGTATGCGCGAGTCATCAGCT
CGTGCAGACTACGTCCCTGCCCT--

>KU647707_Theileria_haneyi

CAGTTATAGTTTATTTGATGTTAGTTTCTACATGGATAACCGTGCTAATTGTAGGGCTAATACATGTTTG
CTGTCAGTTGCGTTTATTAGACCTAAAACCTCCCCGCTTTTGCGGTGTATCGGTGATTCATAATAAATT
AGCGAATCGCATGGCTTTGCTGGCGATGTATCATTCAAGTTTCTGACCTATCAGCTTTGGACGGTAG

GGTATTGGCCTACCGGGGCAACGACGGGTAACGGGGAATTAGGGTTCGATTCCGGAGAGGGAGC
CTGAGAAACGGCTACCACATCTAAGGAAGGCAGCAGGCGCGCAAATTACCCAATCCTGACACAG
GGAGGTAGTGACAAGAAATAACAATACGGGGCTTTAAGTCTTGTAATTGGAATGATGGGAATTTAAAC
CTCTTCCAGAGTATCAATTGGAGGGCAAGTCTGGTGCCAGCAGCCGCGGTAATTCCAGCTCCAATA
GCGTATATTAAACTTGTTGCAGTTAAAAAGCTCGTAGTTGAATTTCTGCTGTATCGTTATCTTCTGC-
TTGACAGTTTGGTATCGTTATGGCTT-GTTGGGTCAC---
TTTGTGTCCCGGCGTTTACTTTGAGAAAATTAGAGTGCTTCAAGCAGGCTTTTGCCTTGAATACTTTAG
CATGGAATAATGGAGTAGGACTTTGGTTCTATTTTGTTGGTTTTAGGAGCCAGAGTAATGGTTAATAGG
AACAGTTGGGGGCATTCGTATTTGACTGTCAGAGGTGAAATTCTTAGATTTGTCAAAGACGAACTACT
GCGAAAGCATTTGCCAAGGATGTTTTCATTAATCAAGAACGAAAGTTAGGGGATCGAAGACGATCAG
ATACCGTCGTAGTCCTAACTATAAACGATGCCGACTAGAGATTGGAGGTCGTCAGTTTGAACGACTC
CTTCAGCACCTTGAGAGAAATCAAAGTCTTTGGGTTCTGGGGGGAGTATGGTCGCAAGGCTGAAAC
TTAAAGGAATTGACGGAAGGGCACCACCAGGCGTGGAGCCTGCGGCTTAATTTGACTCAACACGG
GGAAACTCACCAGGTCCAGACAGAGGAAGGATTGACAGATTGATGGCTCTTTCTTGATTCTTTGGGT
GGTGGTGCATGGCCGTTCTTAGTTGGTGGAGTGATTTGTCTGGTTAATTCCGTTAACGAACGAGACC
TTAACCTGCTAAATAGGGTGTTGGAGTTATGTTCTACACTGCTTCTTAGAGGGACTTTGCGGTCATAAA
TCGCAAGGAAGTTTAAGGCAATAACAGGTCTGTGATGCCCTTAGATGTCCTGGGCTGCACGCGCG
CTACACTGATGCATTCACTGAGTGTATCCTTGGCTGAGAGGCTTGGGTAATCTTGAGTATGCATCGT
GATGGGGATTGATTATTGTAATTCTTAATCATGAACGAGGAATGCCTAGTATGCGCGAGTCATCAGCT
CGTGCAGACTACGTCCCTGCCCT--

>KU647704_Theileria_haneyi

CAGTTATAGTTTATTTGATGTTAGTTTCTACATGGATAACCGTGCTAATTGTAGGGCTAATACATGTTTG
CTGTCAGTTGCGTTTATTAGACCTAAAACCTCCCCGCTTTTGCGGTGTATCGGTGATTCATAATAAATT
AGCGAATCGCATGGCTTTGCTGGCGATGTATCATTCAAGTTTCTGACCTATCAGCTTTGGACGGTAG
GGTATTGGCCTACCGGGGCAACGACGGGTAACGGGGAATTAGGGTTCGATTCCGGAGAGGGAGC
CTGAGAAACGGCTACCACATCTAAGGAAGGCAGCAGGCGCGCAAATTACCCAATCCTGACACAG
GGAGGTAGTGACAAGAAATAACAATACGGGGCTTTAAGTCTTGTAATTGGAATGATGGGAATTTAAAC
CTCTTCCAGAGTATCAATTGGAGGGCAAGTCTGGTGCCAGCAGCCGCGGTAATTCCAGCTCCAATA
GCGTGTATTAAACTCGTTGCAGTTAAAAAGCTCGTAGTTGAATTTCTGCTGTATCGTTATCTTCTGC-
TTGACAGTTTGGTATCGTTATGGCTT-GTTGGGTCAC---
TTTGTGTCCCGGCGTTTACTTTGAGAAAATTAGAGTGCTTCAAGCAGGCTTTTGCCTTGAATACTTTAG
CATGGAATAATGGAGTAGGACTTTGGTTCTATTTTGTTGGTTTTAGGAGCCAGAGTAATGGTTAATAGG
AACAGTTGGGGGCATTCGTATTTGACTGTCAGAGGTGAAATTCTTAGATTTGTCAAAGACGAACTACT
GCGAAAGCATTTGCCAAGGATGTTTTCATTAATCAAGAACGAAAGTTAGGGGATCGAAGACGATCAG
ATACCGTCGTAGTCCTAACTATAAACGATGCCGACTAGAGATTGGAGGTCGTCAGTTTGAACGACTC
CTTCAGCACCTTGAGAGAAATCAAAGTCTTTGGGTTCTGGGGGGAGTATGGTCGCAAGGCTGAAAC
TTAAAGGAATTGACGGAAGGGCACCACCAGGCGTGGAGCCTGCGGCTTAATTTGACTCAACACGG
GGAAACTCACCAGGTCCAGACAGAGGAAGGATTGACAGATTGATGGCTCTTTCTTGATTCTTTGGGT
GGTGGTGCATGGCCGTTCTTAGTTGGTGGAGTGATTTGTCTGGTTAATTCCGTTAACGAACGAGACC
TTAACCTGCTAAATAGGGTGTTGGAGTTATGTTCTACACTGCTTCTTAGAGGGACTTTGCGGTCATAAA
TCGCAAGGAAGTTTAAGGCAATAACAGGTCTGTGATGCCCTTAGATGTCCTGGGCTGCACGCGCG
CTACACTGATGCATTCACTGAGTGTATCCTTGGCTGAGAGGCTTGGGTAATCTTGAGTATGCATCGT
GATGGGGATTGATTATTGTAATTCTTAATCATGAACGAGGAATGCCTAGTATGCGCGAGTCATCAGCT
CGTGCAGACTACGTCCCTGCCCT--

>KU647705_Theileria_haneyi

CAGTTATAGTTTATTTGATGTTAGTTTCTACATGGATAACCGTGCTAATTGTAGGGCTAATACATGTTTG
CTGTCAGTTGCGTTTATTAGACCTAAAACCTCCCCGCTTTTGCGGTGTATCGGTGATTCATAATAAATT
AGCGAATCGCATGGCTTTGCTGGCGATGTATCATTCAAGTTTCTGACCTATCAGCTTTGGACGGTAG
GGTATTGGCCTACCGGGGCAACGACGGGTAACGGGGAATTAGGGTTCGATTCCGGAGAGGGAGC
CTGAGAAACGGCTACCACATCTAAGGAAGGCAGCAGGCGCGCAAATTACCCAATCCTGACACAG
GGAGGTAGTGACAAGAAATAACAATACGGGGCTTTAAGTCTTGTAATTGGAATGATGGGAATTTAAAC
CTCTTCCAGAGTATCAATTGGAGGGCAAGTCTGGTGCCAGCAGCCGCGGTAATTCCAGCTCCAATA
GCGTATATTAAACTTGTTGCAGTTAAAAAGCTCGTAGTTGAATTTCTGCTGTATCGTTATCTTCTGC-
TTGACAGTTTGGTATCGTTATGGCTT-GTTGGGTCAC---
TTTGTGTCCCGGCGTTTACTTTGAGAAAATTAGAGTGCTTCAAGCAGGCTTTTGCCTTGAATACTTTAG
CATGGAATAATGGAGTAGGACTTTGGTTCTATTTTGTTGGTTTTAGGAGCCAGAGTAATGGTTAATAGG
AACAGTTGGGGGCATTCGTATTTGACTGTCAGAGGTGAAATTCTTAGATTTGTCAAAGACGAACTACT
GCGAAAGCATTTGCCAAGGATGTTTTCATTAATCAAGAACGAAAGTTAGGGGATCGAAGACGATCAG
ATACCGTCGTAGTCCTAACTATAAACGATGCCGACTAGAGATTGGAGGTCGTCAGTTTGAACGACTC
CTTCAGCACCTTGAGAGAAATCAAAGTCTTTGGGTTCTGGGGGGAGTATGGTCGCAAGGCTGAAAC
TTAAAGGAATTGACGGAAGGGCACCACCAGGCGTGGAGCCTGCGGCTTAATTTGACTCAACACGG
GGAAACTCACCAGGTCCAGACAGAGGAAGGATTGACAGATTGATGGCTCTTTCTTGATTCTTTGGGT
GGTGGTGCATGGCCGTTCTTAGTTGGTGGAGTGATTTGTCTGGTTAATTCCGTTAACGAACGAGACC
TTAACCTGCTAAATAGGGTGTTGGAGTTATGTTCTACACTGCTTCTTAGAGGGACTTTGCGGTCATAAA
TCGCAAGGAAGTTTAAGGCAATAACAGGTCTGTGATGCCCTTAGATGTCCTGGGCTGCACGCGCG
CTACACTGATGCATTCACTGAGTGTATCCTTGGCTGAGAGGCTTGGGTAATCTTGAGTATGCATCGT
GATGGGGATTGATTATTGTAATTCTTAATCATGAACGAGGAATGCCTAGTATGCGCGAGTCATCAGCT
CGTGCAGACTACGTCCCTGCCCT--

>AB515310_Sudan

-----TCATTAC-
ACAGTTATAGTTTATTTGATGTTGGTTTTTACATGGATAACCGTGTTAAAAGAAGGGCTAATACATGTTT
GGGCACAGTTGCATTTATTAAACCTAAAACCTCCCCGCTTTTGCGGTGTTTCGGTGATTCATAATAAAT
TAGCGAATCGCATGGCTTTGCTGGCGATGTATCATTCAAGTTTCTGACCTATCAGCTTTGGACGGTAG
GGTATTGGCCTACCGGGGCAACGACGGGTAACGGGGAATTAGGGTTCGATTCCGGAGAGGGAGC
CTGAGAAACGGCTACCACATCTAAGGAAGGCAGCAGGCGCGCAAATTACCCAATCCTGACACAG
GGAGGTAGTGACAAGAAATAACAATACGGGGCTTGAAGTCTTGTAATTGGAATGATGGGAATTTAAAC
CCCTTCCAGAGTATCAATTGGAGGGCAAGTCTGGTGCCAGCAGCCGCGGTAATTCCAGCTCCAAT
AGCGTATATTAAACTTGTTGCAGTTAAAAAGCTCGTAGTTGAATTTCTGCTGCATCGTGGTTCTTCGCT
ATGTCGAGTGGGCCTCGTTGTGGCTTAGTTGGGGCATGTTTTCATGACTCGACGTTTACTTTGAGAAA
ATTAGAGTGCTTCAAGCAGGCTTTTGCCTTGAATACTTTAGCATGGAATAATGGAGTAGGACTTTGGTT
CTATTTTGTTGGTTTTAGGAGCCGGAGTAATGGTTAATAGGAACAGTTGGGGGCATTCGTATTTGACT
GTCAGAGGTGAAATTCTTAGATTTGTCAAAGACGAACTACTGCGAAAGCATTTGCCAAGGATGTTTTC
ATTAATCAAGAACGAAAGTTAGGGGATCGAAGACGATCAGATACCGTCGTAGTCCTAACCATAAACG
ATGCCGACTAGAGATTGGAGGTCGTCAGTTTGAACGACTCCTTCAGCACCTTGAGAGAAATCAAAGT
CTTTGGGTTCTGGGGGGAGTATGGTCGCAAGGCTGAAACTTAAAGGAATTGACGGAAGGGCACCA
CCAGGCGTGGAGCCTGCGGCTTAATTTGACTCAACACGGGGAAACTCACCAGGTCCAGACAGAG
GAAGGATTGACAGATTGATAGCTCTTTCTTGATTCTTTGGGTGGTGGTGCATGGCCGTTCTTAGTTGGT
GGAGTGATTTGTCTGGTTAATTCCGTTAACGAACGAGACCTTAACCTGCTAAATAGGATGTGAGATTC
GGTCTCACCATCGCTTCTTAGAGGGACTTTGCGGTCATAAATCGCAAGGAAGTTTAAGGCAATAACA
GGTCTGTGATGCCCTTAGATGTCCTGGGCTGCACGCGCGCTACACTGATGCATTCACTGAGTGTAT
CCTTGGCTGAGAGGCCTGGGTAACCTTGAGTATGCATCGTGATGGGGATTGATTATTGTAATTCTTAA

TCATGAACGAGGAATGCCTAGTATGCGCAAGTCATCAGCTTGTGCAGACTACGTCCCTGCCCTTTG
TACACACCGCCCGTCGCTCCTACCGATCGAGTGATCCG

>AB515311_Sudan

TTTCTACATGGATAACCGTGCTAATTGTAGGGCTAATACATGCTTACTGTCAGTTGCGTTTATTAGACC
TAAAACCTCCCCGCTTTTGCGGTGTTTCGGTGATTCATAATAAATTAGCGAATCGCATGGCTTTGCTG
GCGATGTATCATTCAAGTTTCTGACCTATCAGCTTTGGACGGTAGGGTATTGGCCTACCGGGGCAAC
GACGGGTAACGGGGAATTAGGGTTCGATTCCGGAGAGGGAGCCTGAGAAACGGCTACCACATCTA
AGGAAGGCAGCAGGCGCGCAAATTACCCAATCCTGACACAGGGAGGTAGTGACAAGAAATAACAA
TACGGGGCTTTAAGTCTTGTAATTGGAATGATGGGAATTTAAACCTCTTCCAGAGTATCAATTGGAGG
GCAAGTCTGGTGCCAGCAGCCGCGGTAATTCCAGCTCCAATAGCGTATATTAAACTTGTTGCAGTTA
AAAAGCTCGTAGTTGAATTTCTGCTGTATCGTTTTCCTCTGC-
TTGACAGTTGGATTTCGTTATGGCTTAGTTGGGTTACAGTTCTGTTACCTGACGTTTACTTTGAGAAAAT
TAGAGTGCTTCAAGCAGGCTTTTGCCTTGAATACTTTAGCATGGAATAATGGAGTAGGACTTTGGTTCT
ATTTTGTTGGTTTTAGGAGCCAGAGTAATGGTTAATAGGAACAGTTGGGGGCATTCGTATTTGACTGTC
AGAGGTGAAATTCTTAGATTTGTCAAAGACGAACTACTGCGAAAGCATTTGCCAAGGATGTTTTCATTA
ATCAAGAACGAAAGTTAGGGGATCGAAGACGATCAGATACCGTCGTAGTCCTAACTATAAACGATG
CCGACTAGAGATTGGAGGTCGTCAGTTTGAACGACTCCTTCAGCACCTTGAGAGAAATCAAAGTCTT
TGGGTTCTGGGGGGAGTATGGTCGCAAGGCTGAAACTTAAAGGAATTGACGGAAGGGCACCACCA
GGCGTGGAGCCTGCGGCTTAATTTGACTCAACACGGGGAAACTCACCAGGTCCAGACAGAGGAA
GGATTGACAGATTGATAGCTCTTTCTTGATTCTTTGGGTGGTGGTGCATGGCCGTTCTTAGTTGGTGG
AGTGATTTGTCTGGTTAATTCCGTTAACGAACGAGACCTTAACCTGCTAAATAGGGTGTTGGATTCTA
GTTCTACACTGCTTCTTAGAGGGACTTTGCGGTCATAAATCGCAAGGAAGTTTAAGGCAATAACAGGT
CTGTGATGCCCTTAGATGTCCTGGGCTGCACGCGCGCTACACTGATGCATTCACTGAGTGTATCCT
TGGCTGAGAGGCTTGGGTAATCTTGAGTATGCATCGTGATGGGGATTGATTATTGTAATTCTTAATCAT
GAACGAGGAATGCCTAGTATGCGCAAGTCATCAGCTTGTGCAGACTACGTCCCTGCCCTTTGTACA
CACCGCCCGTCGCTCCTACCGATCGAGTGATCCG

>AB515312_Sudan

--
GGCTCATTACAACAGTTATAGTTTATTTGATGTTAGTTTCTACATGGATAACCGTGCTAATTGTAGGGCT
AATACATGCTTGCTGTCAGTTGCGTTTATTAAACCTAAAACCTCCCCGCTTTTGCGGTGTTTCGGTGAT
TCATAATAAATTAGCGAATCGCATGGCTTTGCTGGCGATGTATCATTCAAGTTTCTGACCTATCAGCTT
TGGACGGTAGGGTATTGGCCTACCGGGGCAACGACGGGTAACGGGGAATTAGGGTTCGATTCCG
GAGAGGGAGCCTGAGAAACGGCTACCACATCTAAGGAAGGCAGCAGGCGCGCAAATTACCCAAT
CCTGACACAGGGAGGTAGTGACAAGAAATAACAATACGGGGCTTTAAGTCTTGTAATTGGAATGATG
GGAATTTAAACCTCTTCCAGAGTATCAATTGGAGGGCAAGTCTGGTGCCAGCAGCCGCGGTAATTC
CAGCTCCAATAGCGTATATTAAACTTGTTGCAGTTAAAAAGCTCGTAGTTGAATTTCTGCTGTATCGTTT
TCCTCTGC-
TTGACAGTTGGATTTCGTTATGGCTTAGTTGGGTTACAGACTTGTTACCCAACGTTTACTTTGAGAAAAT
TAGAGTGCTTCAAGCAGGCTTTTGCCTTGAATACTTTAGCATGGAATAATGGAGTAGGACTTTGGTTCT
ATTTTGTTGGTTTTAGGAGCCAGAGTAATGGTTAATAGGAACAGTTGGGGGCATTCGTATTTGACTGTC
AGAGGTGAAATTCTTAGATTTGTCAAAGACGAACTACTGCGAAAGCATTTGCCAAGGATGTTTTCATTA
ATCAAGAACGAAAGTTAGGGGATCGAAGACGATCAGATACCGTCGTAGTCCTAACTATAAACGATG
CCGACTAGAGATTGGAGGTCGTCAGTTTGAACGACTCCTTCAGCACCTTGAGAGAAATCAAAGTCTT
TGGGTTCTGGGGGGAGTATGGTCGCAAGGCTGAAACTTAAAGGAATTGACGGAAGGGCACCACCA
GGCGTGGAGCCTGCGGCTTAATTTGACTCAACACGGGGAAACTCACCAGGTCCAGACAGAGGAA

GGATTGACAGATTGATAGCTCTTTCTTGATTCTTTGGGTGGTGGTGCATGGCCGTTCTTAGTTGGTGG
AGTGATTTGTCTGGTTAATTCCGTTAACGAACGAGACCTTAACCTGCTAAATAGGGTGTTGGATTCTA
GTTCTACACTGCTTCTTAGAGGGACTTTGCGGTCATAAATCGCAAGGAAGTTTAAGGCAATAACAGGT
CTGTGATGCCCTTAGATGTCCTGGGCTGCACGCGCGCTACACTGATGCATTCACTGAGTGTATCCT
TGGCTGAGAGGCTTGGGTAATCTTGAGTATGCATCGTGATGGGGATTGATTATTGTAATTCTTAATCAT
GAACGAGGAATGCCTAGTATGCGCAAGTCATCAGCTTGTGCAGACTACGTCCCTGCCCTTTGTACA
CACCGCCCGTCGCTCCTACCGATCGAGTGATCCG

>AB515313_Sudan

ATGGCTCATTA-
AACAGTTATAGTTTATTTGATGTTAGTTTCTACATGGATAACCGTGCTAAAAGAAGGGCTAATACATGCT
TGCTGTCAGTTGCGTTTATTATACCTAAAACCTCCCCGCTTTTGCGGTGTTTCGGTGATTCATAATAAA
TTAGCGAATCGCATGGCTTTGCTGGCGATGTATCATTCAAGTTTCTGACCTATCAGCTTTGGACGGTA
GGGTATTGGCCTACCGGGGCAACGACGGGTAACGGGGAATTAGGGTTCGATTCCGGAGAGGGAG
CCTGAGAAACGGCTACCACATCTAAGGAAGGCAGCAGGCGCGCAAATTACCCAATCCTGACACA
GGGAGGTAGTGACAAGAAATAACAATACGGGGCTTTAAGTCTTGTAATTGGAATGATGGGAATTTAAA
CCTCTTCCAGAGTATCAATTGGAGGGCAAGTCTGGTGCCAGCAGCCGCGGTAATTCCAGCTCCAAT
AGCGTATATTAAACTTGTTGCAGTTAAAAAGCTCGTAGTTGAATTTCTGCTGTATCGTTTTCCTCTGC-
TTGACAGTTGGATTTCGTTATGGCTTAGTTGGGTTACAGCCTTGTTACCCAACGTTTACTTTGAGAAAA
TTAGAGTGCTTCAAGCAGGCTTTTGCCTTGAATACTTTAGCATGGAATAATGGAGTAGGACTTTGGTTC
TATTTTGTTGGTTTTAGGAGCCAGAGTAATGGTTAATAGGAACAGTTGGGGGCATTCGTATTTGACTGT
CAGAGGTGAAATTCTTAGATTTGTCAAAGACGAACTACTGCGAAAGCATTTGCCAAGGATGTTTTCAT
TAATCAAGAACGAAAGTTAGGGGATCGAAGACGATCAGATACCGTCGTAGTCCTAACTATAAACGAT
GCCGACTAGAGATTGGAGGTCGTCAGTTTGAACGACTCCTTCAGCACCTTGAGAGAAATCAAAGTC
TTTGGGTTCTGGGGGGAGTATGGTCGCAAGGCTGAAACTTAAAGGAATTGACGGAAGGGCACCAC
CAGGCGTGGAGCCTGCGGCTTAATTTGACTCAACACGGGGAAACTCACCAGGTCCAGACAGAGG
AAGGATTGACAGATTGATAGCTCTTTCTTGATTCTTTGGGTGGTGGTGCATGGCCGTTCTTAGTTGGTG
GAGTGATTTGTCTGGTTAATTCCGTTAACGAACGAGACCTTAACCTGCTAAATAGGGTGTTGGATTCT
AGTTCTACACTGCTTCTTAGAGGGACTTTGCGGTCATAAATCGCAAGGAAGTTTAAGGCAATAACAG
GTCTGTGATGCCCTTAGATGTCCTGGGCTGCACGCGCGCTACACTGATGCATTCACTGAGTGTATC
CTTGGCTGAGAGGCTTGGGTAATCTTGAGTATGCATCGTGATGGGGATTGATTATTGTAATTCTTAATC
ATGAACGAGGAATGCCTAGTATGCGCAAGTCATCAGCTTGTGCAGACTACGTCCCTGCCCTTTGTA
CACACCGCCCGTCGCTCCTACCGATCGAGTGTCGGG

>AB515314_Sudan

--
GGCTCATTACAACAGTTATAGTTTATTTGATGTTAGTTTCTACATGGATAACCGTGCTAATAGTAGGGCT
AATACATGCTTGCTGTCAGTTGCGTTTATTAAACCTAAAACCTCCCCGCTTTTGCGGTGTTTCGGTGAT
TCATAATAAATTAGCGAATCGCATGGCTTTGCTGGCGATGTATCATTCAAGTTTCTGACCTATCAGCTT
TGGACGGTAGGGTATTGGCCTACCGGGGCAACGACGGGTAACGGGGAATTAGGGTTCGATTCCG
GAGAGGGAGCCTGAGAAACGGCTACCACATCTAAGGAAGGCAGCAGGCGCGCAAATTACCCAAT
CCTGACACAGGGAGGTAGTGACAAGAAATAACAATACGGGGCTTTAAGTCTTGTAATTGGAATGATG
GGAATTTAAACCTCTTCCAGAGTATCAATTGGAGGGCAAGTCTGGTGCCAGCAGCCGCGGTAATTC
CAGCTCCAATAGCGTATATTAAACTTGTTGCAGTTAAAAAGCTCGTAGTTGAATTTCTGCTGTATCGTTT
TCCTCTGC-
TTGACAGTTGGATTTCGTTATGGCTTAGTTGGGTTACAGCCTTGTTACCCAACGTTTACTTTGAGAAAA
TTAGAGTGCTTCAAGCAGGCTTTTGCCTTGAATACTTTAGCATGGAATAATGGAGTAGGACTTTGGTTC
TATTTTGTTGGTTTTAGGAGCCAGAGTAATGGTTAATAGGAACAGTTGGGGGCATTCGTATTTGACTGT

CAGAGGTGAAATTCTTAGATTTGTCAAAGACGAACTACTGCGAAAGCATTTGCCAAGGATGTTTTCAT
TAATCAAGAACGAAAGTTAGGGGATCGAAGACGATCAGATACCGTCGTAGTCCTAACTATAAACGAT
GCCGACTAGAGATTGGAGGTCGTCAGTTTGAACGACTCCTTCAGCACCTTGAGAGAAATCAAAGTC
TTTGGGTTCTGGGGGGAGTATGGTCGCAAGGCTGAAACTTAAAGGAATTGACGGAAGGGCACCAC
CAGGCGTGGAGCCTGCGGCTTAATTTGACTCAACACGGGGAAACTCACCAGGTCCAGACAGAGG
AAGGATTGACAGATTGATAGCTCTTTCTTGATTCTTTGGGTGGTGGTGCATGGCCGTTCTTAGTTGGTG
GAGTGATTTGTCTGGTTAATTCCGTTAACGAACGAGACCTTAACCTGCTAAATAGGGTGTTGGATTCT
AGTTCTACACTGCTTCTTAGAGGGACTTTGCGGTCATAAATCGCAAGGAAGTTTAAGGCAATAACAG
GTCTGTGATGCCCTTAGATGTCCTGGGCTGCACGCGCGCTACACTGATGCATTCACTGAGTGTATC
CTTGGCTGAGAGGCTTGGGTAATCTTGAGTATGCATCGTGATGGGGATTGATTATTGTAATTCTTAATC
ATGAACGAGGAATGCCTAGTATGCGCAAGTCATCAGCTTGTGCAGACTACGTCCCTGCCCTTTGTA
CACACCGCCCGTCGCTCCTACCGATCGAGTGACCGG

>AY534882_Spain

ATGGCTCATTACAACAGTTATAGTTTATTTGATGTTGGTTTTTACATGGATAACCGTGCTAATTGTAGGG
CTAATACATGTTTGGGCACAGTTGCATTTATTAGACCTAAAACCTCCCCGCTTTTGCGGTGTTTCGGT
GATTCATAATAAATTAGCGAATCGCATGGCTTTGCTGGCGATGTATCATTCAAGTTTCTGACCTATCAG
CTTTGGACGGTAGGGTATTGGCCTACCGGGGCAACGACGGGTAACGGGGAATTAGGGTTCGATTC
CGGAGAGGGAGCCTGGGAAACGGCTACCACATCTAAGGAAGGCAGCAGGCGCGCAAATTACCC
AATCCTGACACAGGGAGGTAGTGACAAGAAATAACAATACAGGGCTTGAAGTCTTGTAATTGGAATG
ATGGGAATTTAAACCCCTTCCAGAGTATCAATTGGAGGGCAAGTCTGGTGCCAGCAGCCGCGGTAA
TTCCAGCTCCAATAGCGTATATTAAACTTGTTGCAGTTAAAAAGCTCGTAGTTGAATTTCTGCTGCATC
GTGGTTCTTCGCTATGTCGAGTGATCTTCGTTGTGGCTTAGTTGGGGCATGTTTTCATGACTCGACGT
TTACTTTGAGAAAATTAGAGTGCTTCAAGCAGGCTTTTGCCTTGAATACTTTAGCATGGAATAATGGAG
TAGGACTTTGGTTCTATTTTGTTGGTTTTAGGAGCCGGAGTAATGGTTAATAGGAACAGTTGGGGGCA
TTCGTATTTGACTGTCAGAGGTGAAATTCTTAGATTTGTCAAAGACGAACTACTGCGAAAGCATTTGC
CAAGGATGTTTCCATTAATCAAGAACGAAAGTTAGGGGATCGAAGACGATCAGATACCGTCGTAGTC
CTAACCATAAACGATGCCGACTAGAGATTGGAGGTCGTCAGTTTGAACGACTCCTTCAGCACCTTG
AGAGAAATCAAAGTCTTTGGGTTCTGGGGGGAGTATGGTCGCAAGGCTGAAACTTAAAGGAATTGAC
GGAAGGGCACCACCAGGCGTGGAGCCTGCGGCTTAATTTGACTCAACACGGGGAAACTCACCAG
GTCCAGACAGAGGAAGGATTGACAGATTGATAGCTCTTTCTTGATTCTTTGGGTGGTGGTGCATGGC
CGTTCTTAGTTGGTGGAGTGATTTGTCTGGTTAATTCCGTTAACGAACGAGACCTTAACCTGCTAAATA
GGATGCGAGATTTGGTCTCGTTATCGCTTCTTAGAGGGACTTTGCGGTCATAAATCGCAAGGAAGTTT
AAGGCAATAACAGGTCTGTGATGCCCTTAGATGTCCTGGGCTGCACGCGCGCTACACTGATGCATT
CACTGAGTGTATCCTTGGCTGAAAGGCTCGGGTAATCTTGAGTATGCATCGTGATGGGGATTGATTAT
TGTAATTCTTAATCATGAACGAGGAATGCCTAGTATGCGCAAGTCATCAGCTTGTGCAGACTACGTC
CCTGCCCTTTGTACACACCGCCCGTCGCTCCTACCGATCGAGTGATCCG

>DQ287951_Spain

ATGGCTCATTACAACAGTTATAGTTTATTTGATGTTGGTTTTTACATGGATAACCGTGCTAATTGTAGGG
CTAATACATGTTTGGGCACAGTTGCATTTATTAGACCTAAAACCTCCCCGCTTTTGCGGTGTTTCGGT
GATTCATAATAAATTAGCGAATCGCATGGCTTTGCTGGCGATGTATCATTCAAGTTTCTGACCTATCAG
CTTTGGACGGTAGGGTATTGGCCTACCGGGGCAACGACGGGTAACGGGGAATTAGGGTTCGATTC
CGGAGAGGGAGCCTGAGAAACGGCTACCACATCTAAGGAAGGCAGCAGGCGCGCAAATTACCCA
ATCCTGACACAGGGAGGTAGTGACAAGAAATAACAATACGGGGCTTGAAGTCTTGTAATTGGAATGA
TGGGAATTTAAACCCCTTCCAGAGTATCAATTGGAGGGCAAGTCTGGTGCCAGCAGCCGCGGTAAT
TCCAGCTCCAATAGCGTATATTAAACTTGTTGCAGTTAAAAAGCTCGTAGTTGAATTTCTGCTGCATCG
TGGTTCTTCGCTATGTCGAGTGATCTTCGTTGTGGCTTAGTTGGGGCATGTTTTCATGACTCGACGTTT

ACTTTGAGAAAATTAGAGTGCTTCAAGCAGGCTTTTGCCTTGAATACTTTAGCATGGAATAATGGAGTA
GGACTTTGGTTCTATTTTGTTGGTTTTAGGAGCCGGAGTAATGGTTAATAGGAACAGTTGGGGGCATT
CGTATTTGACTGTCAGAGGTGAAATTCTTAGATTTGTCAAAGACGAACTACTGCGAAAGCATTTGCCA
AGGATGTTTCCATTAATCAAGAACGAAAGTTAGGGGATCGAAGACGATCAGATACCGTCGTAGTCCT
AACCATAAACGATGCCGACTAGAGATTGGAGGTCGTCAGTTTGAACGACTCCTTCAGCACCTTGAG
AGAAATCAAAGTCTTTGGGTTCTGGGGGGAGTATGGTCGCAAGGCTGAAACTTAAAGGAATTGACG
GAAGGGCACCACCAGGCGTGGAGCCTGCGGCTTAATTTGACTCAACACGGGGAAACTCACCAGG
TCCAGACAGAGGAAGGATTGACAGATTGATAGCTCTTTCTTGATTCTTTGGGTGGTGGTGCATGGCC
GTTCTTATTTGGTGGAGTGATTTGCCTGGTTAATTCCGCTAACGAACGACACCTTAACCTGCTAAATA
GGATGCGAGATTTGGTCTCGTTATCGCTTCTTAGAGGGACTTTGCGGTCATAAATCGCAAGGAAGTTT
AAGGCAATAACAGGTCTGTGATGCCCTTAGATGTCCTGGGCTGCACGCGCGCTACACTGATGCATT
CACTGAGTGTATCCTTGGCTGAAAGGCTCGGGTAATCTTGAGTATGCATCGCGATGGGGATTGATTA
TTGTAATTCTTAATCATGAACGAGGAATGCTTGGTATGCGCAAGTCATCAGCTTGTGCAGAATACGTC
CCTGCCCTTTGTACACACCGCCCGTCGCTCCTACCGATCGAGTGATCCG

>EU642507_South Africa

ATGGCTCATTACAACAGTTATAGTTTATTTGATGTTGGTTTTTACATGGATAACCGTGCTAATTGTAGGG
CTAATACATGTTTGGGCACAGTTGCATTTATTAGACCTAAAACCTCCCCGCTTTTGCGGTGTTTCGGT
GATTCATAATAAATTAGCGAATCGCATGGCTTTGCTGGCGATGTATCATTCAAGTTTCTGACCTATCAG
CTTTGGACGGTAGGGTATTGGCCTACCGGGGCAACGACGGGTAACGGGGAATTAGGGTTCGATTC
CGGAGAGGGAGCCTGAGAAACGGCTACCACATCTAAGGAAGGCAGCAGGCGCGCAAATTACCCA
ATCCTGACACAGGGAGGTAGTGACAAGAAATAACAATACGGGGCTTGAAGTCTTGTAATTGGAATGA
TGGGAATTTAAACCCCTTCCAGAGTATCAATTGGAGGGCAAGTCTGGTGCCAGCAGCCGCGGTAAT
TCCAGCTCCAATAGCGTATATTAAACTTGTTGCAGTTAAAAAGCTCGTAGTTGAATTTCTGCTGCATCG
TGGTCCTTCGCTATGTCGAGTGGTCCTCGTTGTGGCTTAGTTGGGGCATGTTTTCATGACTCGACGTT
TACTTTGAGAAAATTAGAGTGCTTCAAGCAGGCTTTTGCCTTGAATACTTTAGCATGGAATAATGGAGT
AGGACTTTGGTTCTATTTTGTTGG-
TTTAGGAGCCGGAGTAATGGTTAATAGGAACAGTTGGGGGCATTCGTATTTGACTGTCAGAGGTGAA
ATTCTTAGATTTGTCAAAGACGAACTACTGCGAAAGCATTTGCCAAGGATGTTTTCATTAATCAAGAAC
GAAAGTTAGGGGATCGAAGACGATCAGATACCGTCGTAGTCCTAACCATAAACGATGCCGACTAGA
GATTGGAGGTCGTCAGTTTGAACGACTCCTTCAGCACCTTGAGAGAAATCAAAGTCTTTGGGTTCTG
GGGGGAGTATGGTCGCAAGGCTGAAACTTAAAGGAATTGACGGAAGGGCACCACCAGGCGTGGA
GCCTGCGGCTTAATTTGACTCAACACGGGGAAACTCACCAGGTCCAGACAGAGGAAGGATTGACA
GATTGATAGCTCTTTCTTGATTCTTTGGGTGGTGGTGCATGGCCGTTCTTAGTTGGTGGAGTGATTTGT
CTGGTTAATTCCGTTAACGAACGAGACCTTAACCTGCTAAATAGGATGTGAGATTCGGTCTCACCAT
CGCTTCTTAGAGGGACTTTGCGGTCATAAATCGCAAGGAAGTTTAAGGCAATAACAGGTCTGTGATG
CCCTTAGATGTCCTGGGCTGCACGCGCGCTACACTGATGCATTCACTGAGTGTATCCTTGGCTGAG
AGGCCTGGGTAACCTTGAGTATGCATCGTGATGGGGATTGATTATTGTAATTCTTAATCATGAACGAG
GAATGCCTAGTATGCGCAAGTCATCAGCTTGTGCAGACTACGTCCCTGCCCTTTGTACACACCGCC
CGTCGCTCCTACCGATCGAGTGACC--

>EU642509_South Africa

ACAGTTATAGTTTATTTGATGTTAGTTTCTACATGGATAACCGTGCTAATTGTAGGGCTAATACATGCTT
GCTGTCAGTTGCGTTTATTAGACCTAAAACCTCCCCGCTTTTGCGGTGTTTCGGTGATTCATAATAAAT
TAGCGAATCGCATGGCTTTGCTGGCGATGTATCATTCAAGTTTCTGACCTATCAGCTTTGGACGGTAG
GGTATTGGCCTACCGGGGCAACGACGGGTAACGGGGAATTAGGGTTCGATTCCGGAGAGGGAGC
CTGAGAAACGGCTACCACATCTAAGGAAGGCAGCAGGCGCGCAAATTACCCAATCCTGACACAG

GGAGGTAGTGACAAGAAATAACAATACGGGGCTTTAAGTCTTGTAATTGGAATGATGGGAATTTAAAC
CCCTTCCAGAGTATCAATTGGAGGGCAAGTCTGGTGCCAGCAGCCGCGGTAATTCCAGCTCCAAT
AGCGTATATTAAACTTGTTGCAGTTAAAAAGCTCGTAGTTGAATTTCTGCTGTATCGTTTTCCTCTGCTT
TGACAGTTGGATTTCGTTATGGCTTAGTTGGGTTACAGTGCTGTGACCTAACATTTACTTTGAGAAAATT
AGAGTGCTTCAAGCAGGCTTTTGCCTTGAATACTTTAGCATGGAATAATAGAGTAGGACTTTGGTTCTA
TTTTGTTGGTTTTAGGAGCCAGAGTAATGGTTAATAGGAACAGTTGGGGGCATTCGTATTTGACTGTCA
GAGGTGAAATTCTTAGATTTGTCAAAGACGAACTACTGCGAAAGCATTTGCCAAGGATGTTTTCATTAA
TCAAGAACGAAAGTTAGGGGATCGAAGACGATCAGATACCGTCGTAGTCCTAACTATAAACGATGC
CGACTAGAGATTGGAGGTCGTCAGTTTGAACGACTCCTTCAGCACCTTGAGAGAAATCAAAGTCTTT
GGGTTCTGGGGGGAGTATGGTCGCAAGGCTGAAACTTAAAGGAATTGACGGAAGGGCACCACCAG
GCGTGGAGCCTGCGGCTTAATTTGACTCAACACGGGGAAACTCACCAGGTCCAGACAGAGGAAG
GATTGACAGATTGATAGCTCTTTCTTGATTCTTTGGGTGGTGGTGCATGGCCGTTCTTAGTTGGTGGA
GTGATTTGTCTGGTTAATTCCGTTAACGAACGAGACCTTAACCTGCTAAATAGGGTGTTGGATTCTAGT
TCTACACTGCTTCTTAGAGGGACTTTGCGGTCATAAATCGCAAGGAAGTTTAAGGCAATAACAGGTCT
GTGATGCCCTTAGATGTCCTGGGCTGCACGCGCGCTACACTGATGCATTCACTGAGTGTATCCTTG
GCTGAGAGGCTTGGGTAATCTTGAGTATGCATCGTGATGGGGATTGATTATTGTAATTCTTAATCATGA
ACGAGGAATGCCTAGTATGCGCAAGTCATCAGCTTGTGCAGACTACGTCCCTGCCCT----------------
----

>EU642511_South Africa

ATGGCTCATTACAACAGTTATAGTTTATTTGATGTTAGTTTCTACATGGATAACCGTGCTAATTGTAGGG
CTAATACATGTTTGCTGTCAGTTGCGTTTATTAGACCTAAAACCTCCCCGCTTTTGCGGTGTATCGGT
GATTCATAATAAATTAGCGAATCGCATGGCTTTGCTGGCGATG--
TCGTTCAAGTTTCTGACCTATCAGCTTTGGACGGTAGGGTATTGGCCTACCGGGGCAACGACGGGT
AACGGGGAATTAGGGTTCGATTCCGGAGAGGGAGCCTGAGAAACGGCTACCACATCTAAGGAAGG
CAGCAGGCGCGCAAATTACCCAATCCTGACACAGGGAGGTAGTGACAAGAAATAACAATACGGGG
CTTTAAGTCTTGTAATTGGAATGATGGGAATTTAAACCTCTTCCAGAGTATCAATTGGAGGGCAAGTCT
GGTGCCAGCAGCCGCGGTAATTCCAGCTCCAATAGCGTATATTAAACTTGTTGCAGTTAAAAAGCTC
GTAGTTGAATTTCTGCTGTATCGTTATCTTCTGC-TTGACAGTTTGGTATCGTTATGGCTT-
GTTGGGTCAC---
TTTGTGTCCCGGCGTTTACTTTGAGAAAATTAGAGTGCTTCAAGCAGGCTTTTGCCTTGAATACTTTAG
CATGGAATAATGGAGTAGGACTTTGGTTCTATTTTGTTGGTTTTAGGAGCCAGAGTAATGGTTAATAGG
AACAGTTGGGGGCATTCGTATTTGACTGTCAGAGGTGAAATTCTTAGATTTGTCAAAGACGAACTACT
GCGAAAGCATTTGCCAAGGATGTTTTCATTAATCAAGAACGAAAGTTAGGGGATCGAAGACGATCAG
ATACCGTCGTAGTCCTAACTATAAACGATGCCGACTAGAGATTGGAGGTCGTCAGTTTGAACGACTC
CTTCAGCACCTTGAGAGAAATCAAAGTCTTTGGGTTCTGGGGGGAGTATGGTCGCAAGGCTGAAAC
TTAAAGGAATTGACGGAAGGGCACCACCAGGCGTGGAGCCTGCGGCTTAATTTGACTCAACACGG
GGAAACTCACCAGGTCCAGACAGAGGAAGGATTGACAGATTGATGGCTCTTTCTTGATTCTTTGGGT
GGTGGTGCATGGCCGTTCTTAGTTGGTGGAGTGATTTGTCTGGTTAATTCCGTTAACGAACGAGACC
TTAACCTGCTAAATAGGGTGTTGGAGTTATGTTCTACACTGCTTCTTAGAGGGACTTTGCGGTCATAAA
TCGCAAGGAAGTTTAAGGCAATAACAGGTCTGTGATGCCCTTAGATGTCCTGGGCTGCACGCGCG
CTACACTGATGCATTCACTGAGTGTATCCTTGGCTGAGAGGCTTGGGTAATCTTGAGTATGCATCGT
GATGGGGATTGATTATTGTAATTCTTAATCATGAACGAGGAATGCCTAGTATGCGCGAGTCATCAGCT
CGTGCAGACTACGTCCCTGCCCTTTGTACACACCGCCCGTCGCTCCTACCGATCGAGTGACC--

>EU888902_South Africa

ATGGCTCATTAAAACAGTTATAGTTTATTTGATGTTTGTTTCTACATGGATAACCGTGCTAATTGTAGGG
CTAATACATGCTCGGGCACAGTTGCGTTTATTAGACCTAAAACCTCCCCGCTTTTGCGGTGTTTCGG

TGATTCATAATAAATTAGCGAATCGCATGGCTTTGCCGGCGATGTATCATTCAAGTTTCTGACCTATCA
GCTTTGGACGGTAGGGTATTGGCCTACCGGGGCAACGACGGTAACGGGGAATTAGGGTTCGATT
CCGGAGAGGGAGCCTGAGAAACGGCTACCACATCTAAGGAAGGCAGCAGGCGCGCAAATTACC
CAATCCTGACACAGGGAGGTAGTGACAAGAAATAACAATACGGGGCTTGAAGTCTTGTAATTGGAAT
GATGGGAATTTAAACCCCTTCCAGAGTATCAATTGGAGGGCAAGTCTGGTGCCAGCAGCCGCGGT
AATTCCAGCTCCAATAGCGTATATTAAACTTGTTGCAGTTAAAAAGCTCGTAGTTGAATTTCTGCTGTTT
CGTTGGCA---
TCACTTGGTGTTTGTCATCGTTGCGGCTTGGTTGGGTTTCGATTTCGTTTCCCGGCGTTTACTTTGAGA
AAATTAGAGTGCTTGAAGCAGGCTTTTGCCTTGAATACTTTAGCATGGAATAACGGAGTAGGACTTTG
GTTCTATTTTGTTGGTTTTAGGAGCCAGAGTAATGGTTAATAGGAACAGTTGGGGGCATTCGTATTTGA
CTGTCAGAGGTGAAATTCTTAGATTTGTCAAAGACGAACTACTGCGAAAGCATTTGCCAAGGATGTTT
TCATTAATCAAGAACGAAAGTTAGGGGATCGAAGACGATCAGATACCGTCGTAGTCCTAACCATAAA
CGATGCCGACTAGAGATTGGAGGTCGTCAGTTTGAACGACTCCTTCAGCACCTTGAGAGAAATCAA
AGTCTTTGGGTTCTGGGGGGAGTATGGTCGCAAGGCTGAAACTTAAAGGAATTGACGGAAGGGCAC
CACCAGGCGTGGAGCCTGCGGCTTAATTTGACTCAACACGGGGAAACTCACCAGGTCCAGACAG
AGGAAGGATTGACAGATTGATAGCTCTTTCTTGATTCTTTGGGTGGTGGTGCATGGCCGTTCTTAGTT
GGTGGAGTGATTTGTCTGGTTAATTCCGTTAACGAACGAGACCTTAACCTGCTAAATAGGGTGTGAG
ACTTGGTTTCATTTCCGCTTCTTAGAGGGACTTTGCGGTCATAAATCGCAAGGAAGTTTAAGGCAATA
ACAGGTCTGTGATGCCCTTAGATGTCCTGGGCTGCACGCGCGCTACACTGATGCATTCACTGAGTG
TATCCTTGGCTGAGAGGCTTGGGTAATCTTTAGTATGCATCGTGATGGGGATTGATTATTGTAATTCTT
AATCATGAACGAGGAATGCCTAGTATGCGCAAGTCATCAGCTTGTGCAGACTACGTCCCTGCCCTT
TGTACACACCGCCCGTCGCTCCTACCGATCGAGTGATCCG

>EU888903_South Africa

ATGGCTCATTACAACAGTTATAGTTTATTTGATGTTAGTTTCTACATGGATAACCGTGCTAATTGTAGGG
CTAATACATGTTTGCTGTCAGTTGCGTTTATTAGACCTAAAACCTCCCCGCTTTTGCGGTGTATCGGT
GATTCATAATAAATTAGCGAATCGCATGGCTATGCTGGCGATGTATCATTCAAGTTTCTGACCTATCAG
CTTTGGACGGTAGGGTATTGGCCTACCGGGGCAACGACGGTAACGGGGAATTAGGGTTCGATTC
CGGAGAGGGAGCCTGAGAAACGGCTACCACATCTAAGGAAGGCAGCAGGCGCGCAAATTACCCA
ATCCTGACACAGGGAGGTAGTGACAAGAAATAACAATACGGGGCTTTAAGTCTTGTAATTGGAATGA
TGGGAATTTAAACCTCTTCCAGAGTATCAATTGGAGGGCAAGTCTGGTGCCAGCAGCCGCGGTAAT
TCCAGCTCCAATAGCGTATATTAAACTTGTTGCAGTTAAAAAGCTCGTAGTTGAATTTCTGCTGTATCG
TTATCTTCTGC-TTGACAGTTTGATTTCGTTATGGCTTAGTTGGGTCAC---
TTTGTGTCCCGGCGTTTACTTTGAGAAAATTAGAGTGCTTCAAGCAGGCTTTTGCCTTGAATACTTTAG
CATGGAATAATGGAGTAGGACTTTGGTTCTATTTTGTTGGTTTTAGGAGCCAGAGTAATGGTTAATAGG
AACAGTTGGGGGCATTCGTATTTGACTGTCAGAGGTGAAATTCTTAGATTTGTCAAAGACGAACTACT
GCGAAAGCATTTGCCAAGGATGTTTTCATTAATCAAGAACGAAAGTTAGGGGATCGAAGACGATCAG
ATACCGTCGTAGTCCTAACTATAAACGATGCCGACTAGAGATTGGAGGTCGTCAGTTTGAACGACTC
CTTCAGCACCTTGAGAGAAATCAAAGTCTTTGGGTTCTGGGGGGAGTATGGTCGCAAGGCTGAAAC
TTAAAGGAATTGACGGAAGGGCACCACCAGGCGTGGAGCCTGCGGCTTAATTTGACTCAACACGG
GGAAACTCACCAGGTCCAGACAGAGGAAGGATTGACAGATTGATAGCTCTTTCTTGATTCTTTGGGT
GGTGGTGCATGGCCGTTCTTAGTTGGTGGAGTGATTTGTCTGGTTAATTCCGTTAACGAACGAGACC
TTAACCTGCTAAATAGGGTGTTGGAGTAATGTTCTACACTGCTTCTTAGAGGGACTTTGCGGTCATAA
ATCGCAAGGAAGTTTAAGGCAATAACAGGTCTGTGATGCCCTTAGATGTCCTGGGCTGCACGCGCG
CTACACTGATGCATTCACTGAGTGTATCCTTGGCTGAGAGGCTTGGGTAATCTTGAGTATGCATCGT
GATGGGGATTGATTATTGTAATTCTTAATCATGAACGAGGAATGCCTAGTATGCGCGAGTCATCAGCT
CGTGCAGACTACGTCCCTGCCCTTTGTACACACCGCCCGTCGCTCCTACCGATCGAGTGATCCG

>EU888905_Theileria_equi

ATGGCTCATTACAACAGTTATAGTTTATTTGATGTTAGTTTCTACATGGATAACCGTGCTAATTGTAGGG
CTAATACATGTTTGCTGTCAGTTGCGTTTATTAGACCTAAAACCTCCCCGCTTTTGCGGTGTATCGGT
GATTCATAATAAATTAGCGAATCGCATGGCTTTGCTGGCGATGTATCATTCAAGTTTCTGACCTATCAG
CTTTGGACGGTAGGGTATTGGCCTACCGGGGCAACGACGGGTAACGGGGAATTAGGGTTCGATTC
CGGAGAGGGAGCCTGAGAAACGGCTACCACATCTAAGGAAGGCAGCAGGCGCGCAAATTACCCA
ATCCTGACACAGGGAGGTAGTGACAAGAAATAACAATACGGGGCTTTAAGTCTTGTAATTGGAATGA
TGGGAATTTAAACCTCTTCCAGAGTATCAATTGGAGGGCAAGTCTGGTGCCAGCAGCCGCGGTAAT
TCCAGCTCCAATAGCGTATATTAAACTTGTTGCAGTTAAAAAGCTCGTAGTTGAATTTCTGCTGTATCG
TTATCTTCTGC-TTGACAGTTTGGTATCGTTATGGCTT-GTTGGGTCAC---
ATTGTGTCCCGGCGTTTACTTTGAGAAAATTAGAGTGCTTCAAGCAGGCTTTTGCCTTGAATACTTTAG
CATGGAATAATGGAGTAGGACTTTGGTTCTATTTTGTTGGTTTTAGGAGCCAGAGTAATGGTTAATAGG
AACAGTTGGGGGCATTCGTATTTGACTGTCAGAGGTGAAATTCTTAGATTTGTCAAAGACGAACTACT
GCGAAAGCATTTGCCAAGGATGTTTTCATTAATCAAGAACGAAAGTTAGGGGATCGAAGACGATCAG
ATACCGTCGTAGTCCTAACTATAAACGATGCCGACTAGAGATTGGAGGTCGTCAGTTTGAACGACTC
CTTCAGCACCTTGAGAGAAATCAAAGTCTTTGGGTTCTGGGGGGAGTATGGTCGCAAGGCTGAAAC
TTAAAGGAATTGACGGAAGGGCACCACCAGGCGTGGAGCCTGCGGCTTAATTTGACTCAACACGG
GGAAACTCACCAGGTCCAGACAGAGGAAGGATTGACAGATTGATGGCTCTTTCTTGATTCTTTGGGT
GGTGGTGCATGGCCGTTCTTAGTTGGTGGAGTGATTTGTCTGGTTAATTCCGTTAACGAACGAGACC
TTAACCTGCTAAATAGGGTGTTGGAGTTATGTTCTACACTGCTTCTTAGAGGGACTTTGCGGTCATAAA
TCGCAAGGAAGTTTAAGGCAATAACAGGTCTGTGATGCCCTTAGATGTCCTGGGCTGCACGCGCG
CTACACTGATGCATTCACTGAGTGTATCCTTGGCTGAGAGGCTTGGGTAATCTTGAGTATGCATCGT
GATGGGGATTGATTATTGTAATTCTTAATCATGAACGAGGAATGCCTAGTATGCGCGAGTCATCAGCT
CGTGCAGACTACGTCCCTGCCCTTTGTACACACCGCCCGTCGCTCCTACCGATCGAGTG-----

>HM229407_South Korea

ATGGCTCATTACAACAGTTATAGTTTATTTGATGTTGGTTTTTACATGGATAACCGTGCTAATTGTAGGG
CTAATACATGTTTGGGCACAGTTGCATTTATTAGACCTAAAACCTCCCCGCTTTTGCGGTGTTTCGGT
GATTCATAATAAATTAGCGAATCGCATGGCTTTGCTGGCGATGTATCATTCAAGTTTCTGACCTATCAG
CTTTGGACGGTAGGGTATTGGCCTACCGGGGCAACGACGGGTAACGGGGAATTAGGGTTCGATTC
CGGAGAGGGAGCCTGAGAAACGGCTACCACATCTAAGGAAGGCAGCAGGCGCGCAAATTACCCA
ATCCTGACACAGGGAGGTAGTGACAAGAAATAACAATACGGGGCTTGAAGTCTTGTAATTGGAATGA
TGGGAATTTAAACCCCTTCCAGAGTATCAATTGGAGGGCAAGTCTGGTGCCAGCAGCCGCGGTAAT
TCCAGCTCCAATAGCGTATATTAAACTTGTTGCAGTTAAAAAGCTCGTAGTTGAATTTCTGCTGCATCG
TGGTTCTTCGCTATGTCGAGTGATCTTCGTTGTGGCTTAGTTGGGGCATGTTTTCATGACTCGACGTTT
ACTTTGAGAAAATTAGAGTGCTTCAAGCAGGCTTTTGCCTTGAATACTTTAGCATGGAATAATGGAGTA
GGACTTTGGTTCTATTTTGTTGGTTTTAGGAGCCGGAGTAATGGTTAATAGGAACAGTTGGGGGCATT
CGTATTTGACTGTCAGAGGTGAAATTCTTAGATTTGTCAAAGACGAACTACTGCGAAAGCATTTGCCA
AGGATGTTTCCATTAATCAAGAACGAAAGTTAGGGGATCGAAGACGATCAGATACCGTCGTAGTCCT
AACCATAAACGATGCCGACTAGAGATTGGAGGTCGTCAGTTTGAACGACTCCTTCAGCACCTTGAG
AGAAATCAAAGTCTTTGGGTTCTGGGGGGAGTATGGTCGCAAGGCTGAAACTTAAAGGAATTGACG
GAAGGGCACCACCAGGCGTGGAGCCTGCGGCTTAATTTGACTCAACACGGGGAAACTCACCAGG
TCCAGACAGAGGAAGGATTGACAGATTGATAGCTCTTTCTTGATTCTTTGGGTGGTGGTGCATGGCC
GTTCTTAGTTGGTGGAGTGATTTGTCTGGTTAATTCCGTTAACGAACGAGACCTTAACCTGCTAAATAG
GATGCGAGATTTGGTCTCGTTATCGCTTCTTAGAGGGACTTTGCGGTCATAAATCGCAAGGAAGTTTA
AGGCAATAACAGGTCTGTGATGCCCTTAGATGTCCTGGGCTGCACGCGCGCTACACTGATGCATTC
ACTGAGTGTATCCTTGGCTGAAAGGCTCGGGTAATCTTGAGTATGCATCGTGATGGGGATTGATTATT

GTAATTCTTAATCATGAACGAGGAATGCCTAGTATGCGCAAGTCATCAGCTTGTGCAGACTACGTCC
CTGCCCTTTGTACACACCGCCCGTCGCTCCTACCGATCGAGTGATCCG

>HM229408_South Korea

ATGGCTCATTACAACAGTTATAGTTTATTTGATGTTGGTTTTTACATGGATAACCGTGCTAATTGTAGGG
CTAATACATGTTTGGGCACAGTTGCATTTATTAGACCTAAAACCTCCCCGCTTTTGCGGTGTTTCGGT
GATTCATAATAAATTAGCGAATCGCATGGCTTTGCTGGCGATGTATCATTCAAGTTTCTGACCTATCAG
CTTTGGACGGTAGGGTATTGGCCTACCGGGGCAACGACGGGTAACGGGGAATTAGGGTTCGATTC
CGGAGAGGGAGCCTGAGAAACGGCTACCACATCTAAGGAAGGCAGCAGGCGCGCAAATTACCCA
ATCCTGACACAGGGAGGTAGTGACAAGAAATAACAATACGGGGCTTGAAGTCTTGTAATTGGAATGA
TGGGAATTTAAACCCCTTCCAGAGTATCAATTGGAGGGCAAGTCTGGTGCCAGCAGCCGCGGTAAT
TCCAGCTCCAATAGCGTATATTAAACTTGTTGCAGTTAAAAAGCTCGTAGTTGAATTTCTGCTGCATCG
TGGTTCTTCGCTATGTCGAGTGATCTTCGTTGTGGCTTAGTTGGGGCATGTTTTCATGACTCGACGTTT
ACTTTGAGAAAATTAGAGTGCTTCAAGCAGGCTTTTGCCTTGAATACTTTAGCATGGAATAATGGAGTA
GGACTTTGGTTCTATTTTGTTGGTTTTAGGAGCCGGAGTAATGGTTAATAGGAACAGTTGGGGGCATT
CGTATTTGACTGTCAGAGGTGAAATTCTTAGATTTGTCAAAGACGAACTACTGCGAAAGCATTTGCCA
AGGATGTTTCCATTAATCAAGAACGAAAGTTAGGGGATCGAAGACGATCAGATACCGTCGTAGTCCT
AACCATAAACGATGCCGACTAGAGATTGGAGGTCGTCAGTTTGAACGACTCCTTCAGCACCTTGAG
AGAAATCAAAGTCTTTGGGTTCTGGGGGGAGTATGGTCGCAAGGCTGAAACTTAAAGGAATTGACG
GAAGGGCACCACCAGGCGTGGAGCCTGCGGCTTAATTTGACTCAACACGGGGAAACTCACCAGG
TCCAGACAGAGGAAGGATTGACAGATTGATAGCTCTTTCTTGATTCTTTGGGTGGTGGTGCATGGCC
GTTCTTAGTTGGTGGAGTGATTTGTCTGGTTAATTCCGTTAACGAACGAGACCTTAACCTGCTAAATAG
GATGCGAGATTTGGTCTCGTTATCGCTTCTTAGAGGGACTTTGCGGTCATAAATCGCAAGGAAGTTTA
AGGCAATAACAGGTCTGTGATGCCCTTAGATGTCCTGGGCTGCACGCGCGCTACACTGATGCATTC
ACTGAGTGTATCCTTGGCTGAAAGGCTCGGGTAATCTTGAGTATGCATCGTGATGGGGATTGATTATT
GTAATTCTTAATCATGAACGAGGAATGCCTAGTATGCGCAAGTCATCAGCTTGTGCAGACTACGTCC
CTGCCCTTTGTACACACCGCCCGTCGCTCCTACCGATCGAGTGATCCG

>JQ390047_Mexico

ATGGCTCATTACAACAGTTATAGTTTATTTGATGTTAGTTTCTACATGGATAACCGTGCTAATTGTAGGG
CTAATACATGTTTGCTGTCAGTTGCGTTTATTAGACCTAAAACCTCCCCGCTTTTGCGGTGTATCGGT
GATTCATAATAAATTAGCGAATCGCATGGCTTTGCTGGCGATGTATCATTCAAGTTTCTGACCTATCAG
CTTTGGACGGTAGGGTATTGGCCTACCGGGGCAACGACGGGTAACGGGGAATTAGGGTTCGATTC
CGGAGAGGGAGCCTGAGAAACGGCTACCACATCTAAGGAAGGCAGCAGGCGCGCAAATTACCCA
ATCCTGACACAGGGAGGTAGTGACAAGAAATAACAATACGGGGCTTTAAGTCTTGTAATTGGAATGA
TGGGAATTTAAACCTCTTCCAGAGTATCAATTGGAGGGCAAGTCTGGTGCCAGCAGCCGCGGTAAT
TCCAGCTCCAATAGCGTATATTAAACTTGTTGCAGTTAAAAAGCTCGTAGTTGAATTTCTGCTGTATCG
TTATCTTCTGC-TTGACAGTTTGGTATCGTTATGGCTT-GTTGGGTCAC---
TTTGTGTCCCGGCGTTTACTTTGAGAAAATTAGAGTGCTTCAAGCAGGCTTTTGCCTTGAATACTTTAG
CATGGAATAATGGAGTAGGACTTTGGTTCTATTTTGTTGGTTTTAGGAGCCAGAGTAATGGTTAATAGG
AACAGTTGGGGGCATTCGTATTTGACTGTCAGAGGTGAAATTCTTAGATTTGTCAAAGACGAACTACT
GCGAAAGCATTTGCCAAGGATGTTTTCATTAATCAAGAACGAAAGTTAGGGGATCGAAGACGATCAG
ATACCGTCGTAGTCCTAACTATAAACGATGCCGACTAGAGATTGGAGGTCGTCAGTTTGAACGACTC
CTTCAGCACCTTGAGAGAAATCAAAGTCTTTGGGTTCTGGGGGGAGTATGGTCGCAAGGCTGAAAC
TTAAAGGAATTGACGGAAGGGCACCACCAGGCGTGGAGCCTGCGGCTTAATTTGACTCAACACGG
GGAAACTCACCAGGTCCAGACAGAGGAAGGATTGACAGATTGATGGCTCTTTCTTGATTCTTTGGGT
GGTGGTGCATGGCCGTTCTTAGTTGGTGGAGTGATTTGTCTGGTTAATTCCGTTAACGAACGAGACC
TTAACCTGCTAAATAGGGTGTTGGAGTTATGTTCTACACTGCTTCTTAGAGGGACTTTGCGGTCATAAA

TCGCAAGGAAGTTTAAGGCAATAACAGGTCTGTGATGCCCTTAGATGTCCTGGGCTGCACGCGCG
CTACACTGATGCATTCACTGAGTGTATCCTTGGCTGAGAGGCTTGGGTAATCTTGAGTATGCATCGT
GATGGGGATTGATTATTGTAATTCTTAATCATGAACGAGGAATGCCTAGTATGCGCGAGTCATCAGCT
CGTGCAGACTACGTCCCTGCCCTTTGTACACACCGCCCGTCGCTCCTACCGATCGAGTGATCCG

>JX177670_USA

ATGGCTCATTAAAACAGTTATAGTTTATTTGATGTTTGTTTCTACATGGATAACCGTGCTAATTGTAGGG
CTAATACATGCTCGGGCACAGTTGCGTTTATTAGACCTAAAACCTCCCCGCTTTTGCGGTGTTTCGG
TGATTCATAATAAATTAGCGAATCGCATGGCTTTGCCGGCGATGTATCATTCAAGTTTCTGACCTATCA
GCTTTGGACGGTAGGGTATTGGCCTACCGGGGCAACGACGGGTAACGGGGAATTAGGGTTCGATT
CCGGAGAGGGAGCCTGAGAAACGGCTACCACATCTAAGGAAGGCAGCAGGCGCGCAAATTACC
CAATCCTGACACAGGGAGGTAGTGACAAGAAATAACAATACGGGGCTTGAAGTCTTGTAATTGGAAT
GATGGGAATCTAAACCCCTTCCAGAGTATCAATTGGAGGGCAAGTCTGGTGCCAGCAGCCGCGGT
AATTCCAGCTCCAATAGCGTATATTAAACTTGTTGCAGTTAAAAAGCTCGTAGTTGAATTTCTGCTGTTT
CGTTGACT---
GCGTTTGGCGTTTGTCATCGTTGCGGCTTGGTTGGGTTTCGATTTCGTTTCCCGGCGTTTACTTTGAG
AAAATTAGAGTGCTTGAAGCAGGCTTTTGCCTTGAATACTTTAGCATGGAATAACGGAGTAGGACTTT
GGTTCTATTTTGTTGGTTTTAGGAGCCAGAGTAATGGTTAATAGGAACAGTTGGGGGCATTCGTATTTG
ACTGTCAGAGGTGAAATTCTTAGATTTGTCAAAGACGAACTACTGCGAAAGCATTTGCCAAGGATGTT
TTCATTAATCAAGAACGAAAGTTAGGGGATCGAAGACGATCAGATACCGTCGTAGTCCTAACCATAA
ACGATGCCGACTAGAGATTGGAGGTCGTCAGTTTGAACGACTCCTTCAGCACCTTGAGAGAAATCA
AAGTCTTTGGGTTCTGGGGGGAGTATGGTCGCAAGGCTGAAACTTAAAGGAATTGACGGAAGGGCA
CCACCAGGCGTGGAGCCTGCGGCTTAATTTGACTCAACACGGGGAAACTCACCAGGTCCAGACA
GAGGAAGGATTGACAGATTGATGGCTCTTTCTTGATTCTTTGGGTGGTGGTGCATGGCCGTTCTTAGT
TGGTGGAGTGATTTGTCTGGTTAATTCCGTTAACGAACGAGACCTTAACCTGCTAAATAGGGTGTGAG
ACTTGGTTTCATTTCCGCTTCTTAGAGGGACTTTGCGGTCATAAATCGCAAGGAAGTTTAAGGCAATA
ACAGGTCTGTGATGCCCTTAGATGTCCTGGGCTGCACGCGCGCTACACTGATGCATTCACTGAGTG
TATCCTTGGCTGAGAGGCTTGGGTAATCTTGAGTATGCATCGTGATGGGGATTGATTATTGTAATTCTT
AATCATGAACGAGGAATGCCTAGTATGCGCAAGTCATCAGCTTGTGCAGACTACGTCCCTGCCCTT
TGTACACACCGCCCGTCGCTCCTACCGATCGAGTGATCCG

>KF559357_China

ATGGCTCATTACAACAGTTATAGTTTATTTGATGTTGGTTTTTACATGGATAACCGTGCTAATTGTAGGG
CTAATACATGTTTGGGCACAGTTGCATTTATTAGACCTAAAACCTCCCCGCTTTTGCGGTGTTTCGGT
GATTCATAATAAATTAGCGAATCGCATGGCTTTGCTGGCGATGTATCATTCAAGTTTCTGACCTATCAG
CTTTGGACGGTAGGGTATTGGCCTACCGGGGCAACGACGGGTAACGGGGAATTAGGGTTCGATTC
CGGAGAGGGAGCCTGAGAAACGGCTACCACATCTAAGGAAGGCAGCAGGCGCGCAAATTACCCA
ATCCTGACACAGGGAGGTAGTGACAAGAAATAACAATACGGGGCTTGAAGTCTTGTAATTGGAATGA
TGGGAATTTAAACCCCTTCCAGAGTATCAATTGGAGGGCAAGTCTGGTGCCAGCAGCCGCGGTAAT
TCCAGCTCCAATAGCGTATATTAAACTTGTTGCAGTTAAAAAGCTCGTAGTTGAATTTCTGCTGCATCG
TGGTTCTTCGCTATGTCGAGTGATCTTCGTTGTGGCTTAGTTGGGGCATGTTTTCATGACTCGACGTTT
ACTTTGAGAAAATTAGAGTGCTTCAAGCAGGCTTTTGCCTTGAATACTTTAGCATGGAATAATGGAGTA
GGACTTTGGTTCTATTTTGTTGGTTTTAGGAGCCGGAGTAATGGTTAATAGGAACAGTTGGGGGCATT
CGTATTTGACTGTCAGAGGTGAAATTCTTAGATTTGTCAAAGACGAACTACTGCGAAAGCATTTGCCA
AGGATGTTTCCATTAATCAAGAACGAAAGTTAGGGGATCGAAGACGATCAGATACCGTCGTAGTCCT
AACCATAAACGATGCCGACTAGAGATTGGAGGTCGTCAGTTTGAACGACTCCTTCAGCACCTTGAG
AGAAATCAAAGTCTTTGGGTTCTGGGGGGAGTATGGTCGCAAGGCTGAAACTTAAAGGAATTGACG
GAAGGGCACCACCAGGCGTGGAGCCTGCGGCTTAATTTGACTCAACACGGGGAAACTCACCAGG

TCCAGACAGAGGAAGGATTGACAGATTGATAGCTCTTTCTTGATTCTTTGGGTGGTGGTGCATGGCC
GTTCTTAGTTGGTGGAGTGATTTGTCTGGTTAATTCCGTTAACGAACGAGACCTTAACCTGCTAAATAG
GATGCGAGATTTGGTCTCGTTATCGCTTCTTAGAGGGACTTTGCGGTCATAAATCGCAAGGAAGTTTA
AGGCAATAACAGGTCTGTGATGCCCTTAGATGTCCTGGGCTGCACGCGCGCTACACTGATGCATTC
ACTGAGTGTATCCTTGGCTGAAAGGCTCGGGTAATCTTGAGTATGCATCGTGATGGGGATTGATTATT
GTAATTCTTAATCATGAACGAGGAATGCCTAGTATGCGCAAGTCATCAGCTTGTGCAGACTACGTCC
CTGCCCTTTGTACACACCGCCCGTCGCTCCTACCGATCGAGTGATCCG

>KF597073_Kenya

ATGGCTCATTACAACAGTTATAGTTTATTTGATGTTGGTTTTTACATGGATAACCGTGCTAATTGTAGGG
CTAATACATGTTTGGGCACAGTTGCATTTATTAGACCTAAAACCTCCCCGCTTTTGCGGTGTTTCGGT
GATTCATAATAAATTAGCGAATCGCATGGCTTTGCTGGCGATGTATCATTCAAGTTTCTGACCTATCAG
CTTTGGACGGTAGGGTATTGGCCTACCGGGGCAACGACGGGTAACGGGGAATTAGGGTTCGATTC
CGGAGAGGGAGCCTGAGAAACGGCTACCACATCTAAGGAAGGCAGCAGGCGCGCAAATTACCCA
ATCCTGACACAGGGAGGTAGTGACAAGAAATAACAATACGGGGCTTGAAGTCTTGTAATTGGAATGA
TGGGAATTTAAACCCCTTCCAGAGTATCAATTGGAGGGCAAGCCTGGTGCCAGCAGCCGCGGTAA
TTCCAGCTCCAATAGCGTATATTAAACTTGTTGCAGTTAAAAAGCTCGTAGTTGAATTTCTGCTGCATC
GTGGTTCTTCGCTATGTCGAGTGGGCCTCGTTGTGGCTTAGTTGGGGCATGTTTTC-
TGACTCGACGTTTACTTTGAGAAAATTAGAGTGCTTCAAGCAGGCTTTTGCCTTGAATACTTTAGCATG
GAATAATGGAGTAGGACTTTGGTTCTATTTTGTTGGTTTTAGGAGCCGGAGTAATGGTTAATAGGAACA
GTTGGGGGCATTCGTATTTGACTGTCAGAGGTGAAATTCTTAGATTTGTCAAAGACGAACTACTGCGA
AAGCATTTGCCAAGGATGTTTTCATTAATCAAGAACGAAAGTTAGGGGATCGAAGACGATCAGATAC
CGTCGTAGTCCTAACCATAAACGATGCCGACTAGAGATTGGAGGTCGTCAGTTTGAACGACTCCTT
CAGCACCTTGAGAGAAATCAAAGTCTTTGGGTTCTGGGGGGAGTTTGGTCGCAAGGCTGAAACTTA
AAGGAATTGACGGAAGGGCACCACCAGGCGTGGAGCCTGCGGCTTAATTTGACTCAACACGGGG
AAACTCACCAGGTCCAGACAGAGGAAGGATTGACAGATTGATAGCTCTTTCTTGATTCTTTAGGTGGT
GGTGCATGGCCGTTCTTAGTTGGTGGAGTGATTTGTCTGGTTAATTCCGTTAACGAACGAGACCTTAA
CCTGCTAAATAGGATGTGAGATTCGGTCTCACCATCGCTTCTTAGAGGGACTTTGCGGTCATAAATC
GCAAGGAAGTTTAAGGCAATAACAGGTCTGTGATGCCCTTAGATGTCCTGGGCTGCACGCGCGCTA
CACTGATGCATTCACTGAGTGTATCCTTGGCTGAGAGGCCTGGGTAACCTTGAGTATGCATCGTGAT
GGGGATTGATTATTGTAATTCTTAATCATGAACGAGGAATGCCTAGTATGCGCAAGTCATCAGCTTGT
GCAGACTACGTCCCTGCCCTTTGTACACACCGCCCGTCGCTCCTACCGATCGAGTGATCCG

>KF597077_Kenya

ATGGCTCATTACAACAGTTATAGTTTATTTGATGTTGGTTTTTACATGGATAACCGTGCTAATTGTAGGG
CTAATACATGTTTGGGCACAGTTGCATTTATTAGACCTAAAACCTCCCCGCTTTTGCGGTGTTTCGGT
GATTCATAATAAATTAGCGAATCGCATGGCTTTGCTGGCGATGTATCATTCAAGTTTCTGACCTATCAG
CTTTGGACGGTAGGGTATTGGCCTACCGGGGCAACGACGGGTAACGGGGAATTAGGGTTCGATTC
CGGAGAGGGAGCCTGAGAAACGGCTACCACATCTAAGGAAGGCAGCAGGCGCGCAAATTACCCA
ATCCTGACACAGGGAGGTAGTGGCAAGAAATAACAATACGGGGCTTGAAGTCTTGTAATTGGAATGA
TGGGAATTTAAACCCCTTCCAGAGTATCAATTGGAGGGCAAGTCTGGTGCCAGCAGCCGCGGTAAT
TCCAGCTCCAATAGCGTATATTAAACTTGTTGCAGTTAAAAAGCTCGTAGTTGAATTTCTGCTGCATCG
TGGTTCTTCGCTATGTCGAGTGGGCCTCGTTGTGGCTTAGTTGGGGCATGTTTTCATGACTCGACGTT
TACTTTGAGAAAATTAGAGTGCTTCAAGCAGGCTTTTGCCTTGAATACTTTAGCATGGAATAATGGAGT
AGGACTTTGGTTCTATTTTGTTGGTTTTAGGAGCCGGAGTAATGGTTAATAGGAACAGTTGGGGGCAT
TCGTATTTGACTGTCAGAGGTGAAATTCTTAGATTTGTCAAAGACAAACTACTGCGAAAGCATTTGCC
AAGGATGTTTTCATTAATCAAGAACGAAAGTTAGGGGATCGAAGACGATCAGATACCGTCGTAGTCC
TAACCATAAACGATGCCGACTAGAGATTGGAGGTCGTCAGTTTGAACGACTCCTTCAGCACCTTGA

GAGAAATCAAAGTCTTTGGGTTCTGGGGGGAGTATGGTCGCAAGGCTGAAACTTAAAGGAATTGAC
GGAAGGGCACCACCAGGCGTGGAGCCTGCGGCTTAATTTGACTCAACACGGGGAAACTCACCAG
GTCCAGACAGAGGAAGGATTGACAGATTGATAGCTCTTTCTTGATTCTTTGGGTGGTGGTGCATGGC
CGTTCTTAGTTGGTGGAGTGATTTGTCTGGTTAATTCCGTTAACGAACGAGACCTTAACCTGCTAAATA
GGATGTGAGATTCGGTCTCACCATCGCTTCTTAGAGGGACTTTGCAGTCATAAATCGCAAGGAAGTT
TAAGGCAATAACAGGTCTGTGATGCCCTTAGATGTCCTGGGCTGCACGCGCGCTACACTGATGCAT
TCACTGAGTGTATCCTTGGCTGAGAGGCCTGGGTAACCTTGAGTATGCATCGTGATGGGGATTGATT
ATTGTAATTCTTAATCATGAACGAGGAATGCC-
AGTATGCGCAAGTCATCAGCTTGTGCAGACTACGTCCCTGCCCTTTGTACACACCGCCCGTCGCT
CCTACCGATCGAGTGATCCG

>KF597078_Kenya

ATGGCTCATTACAACAGTTATAGTTTATTTGATGTTGGTTTTTACATGGATAACCGTGCTAATTGTAGGG
CTAATACATGTTTGGGCACAGTTGCATTTATTAGACCTAAAACCTCCCCGCTTTTGCGGTGTTTCGGT
GATTCATAATAAATTAGCGAATCGCATGGCTTTGCTGGCGATGTATCATTCGAGTTTCTGACCCATCA
GCTTTGGACGGTAGGGTATTGGCCTACCGGGGCAACGACGGGTAACGGGGAATTAGGGTTCGATT
CCGGAGAGGGAGCCTGAGAAACGGCTACCACATCTAAGGAAGGCAGCAGGCGCGCAAATTACC
CAATCCTGACACAGGGAGGTAGTGACAAGAAATAACAATACGGGGCTTGAAGTCTTGTAATTGGAAT
GATGGGAATTTAAACCCCTTCCAGAGTATCAATTGGAGGGCAAGTCTGGTGCCAGCAGCCGCGGT
AATTCCAGCTCCAATAGCGTATATTAAACTTGTTGCAGTTAAAAAGCTCGTAGTTGAATTTCTGCTGCA
TCGTGGTTCTTCGCTATGTCGAGTGGGCCTCGTTGTGGCTTAGTTGGGGCATGTTTTCATGACTCGA
CGTTTACTTTGAGAAAATTAGAGTGCTTCAAGCAGGCTTTTGCCTTGAATACTTTAGCATGGAATAATG
GAGTAGGACTTTGGTTCTATTTTGTTGGTTTTAGGAGCCGGAGTAATGGTTAATAGGAACAGTTGGGG
GCATTCGTATTTGACTGTCAGAGGTGAAATTCTTAGATTTGTCAAAGACGAACTACTGCGAAAGCATTT
GCCAAGGATGTTTTCATTAATCAAGAACGAAAGTTAGGGGATCGAAGACGATCAGGTACCGTCGTAG
TCCTAACCATAAACGATGCCGACTAGAGATTGGAGGTCGTCAGTTTGAACGACTCCTTCAGCACCTT
GAGAGAAATCAAAGTCTTTGGGTTCTGGGGGGAGTATGGTCGCAAGGCTGAAACTTAAAGGAATTGA
CGGAAGGGCACCACCAGGCGTGGAGCCTGCGGCTTAATTTGACTCAACACGGGGAAACTCACCA
GGTCCAGACAGAGGAAGGATTGACAGATTGATAGCTCTTTCTTGATTCTTTGGGTGGTGGTGCATGG
CCGTTCTTAGTTGGTGGAGTGATTTGTCTGGTTAATTCCGTTAACGAACGAGACCTTAACCTGCTAAA
TAGGATGTGAGATTCGGTCTCACCATCGCTTCTTAGAGGGACTTTGCGGTCATAAATCGCAAGGAAG
TTTAAGGCAATAACAGGTCTGTGATGCCCTTAGATGTCCTGGGCTGCACGCGCGCTACACTGATGC
ATTCACTGAGTGTATCCTTGGCTGAGAGGCCTGGGTAACCTTGAGTATGCATCGTGATGGGGATTGA
TTATTGTAATTCTTAATCATGAACGAGGAATGCCTAGTATGCGCAAGTCATCAGCTTGTGCAGACTAC
GTCCCTGCCCTTTGTACACACCGCCCGTCGCTCCTACCGATCGAGTGATCCG

>KF597081_Kenya

ATGGCTCATTACAACAGTTATAGTTTATTTGATGTTGGTTTTTACATGGATAACCGTGCTAATTGTAGGG
CTAATACATG-
TTGGGCACAGTTGCATTTATTAGACCTAAAACCTCCCCGCTTTTGCGGTGTTTCGGTGATTCATAATAA
ATTAGCGAATCGCATGGCTTTGCTGGCGATGTATCATTCAAGTTTCTGACCTATCAGCTTTGGACGGT
AGGGTATTGGCCTACTGGGGCAACGACGGGTAACGGGGAATTAGGGTTCGATTCCGGAGAGGGA
GCCTGAGAAACGGCTACCACATCTAAGGAAGGCAGCAGGCGCGCAAATTACCCAATCCTGACAC
AGGGAGGTAGTGACAAGAAATAACAATACGGGGCTTGAAGTCTTGTAATTGGAATGATGGGAATTTAA
ACCCCTTCCAGAGTATCAATTGGAGGGCAAGTCTGGTGCCAGCAGCCGCGGTAATTCCAGCTCCA
ATAGCGTATATTAAACTTGTTGCAGTTAAAAAGCTCGTAGTTGAATTTCTGCTGCATCGTGGTTCTTCG
CTATGTCGAGTGGGCCTCGTTGTGGCTTAGTTGGGGCATGTTTTCATGACTCGACGTTTACTTTGAGA
AAATTAGAGTGCTTCAAGCAGGCTTTTGCCTTGAATACTTTAGCATGGAATAATGGAGTAGGACTTTG

GTTCTATTTTGTTGGTTTTAGGAGCCAGAGTAATGGTTAATAGGAACAGTTGGGGGCATTCGTATTTGA
CTGTCAGAGGTGAAATTCTTAGATTTGTCAAAGACGAACTACTGCGAAAGCATTTGCCAAGGATGTTT
TCATTAATCAAGAACGAAAGTTAGGGGATCGAAGACGATCAGATACCGTCGTAGTCCTAACTATAAA
CGATGCCGACTAGAGATTGGAGGTCGTCAGTTTGAACGACTCCTTCAGCACCTTGAGAGAAATCAA
AGTCTTTGGGTTCTGGGGGGAGTATGGTCGCAAGGCTGAAACTTAAAGGAATTGACGGAAGGGCAC
CACCAGGCGTGGAGCCTGCGGCTTAATTTGACTCAACCCGGGGAAACTCACCAGGTCCAGACAG
AGGAAGGATTGACAGATTGATAGCTCTTTCTTGATTCTTTGGGTGGTGGGGCATGGCCGTTCTTAGTT
GGTGGAGTGATTTGTCTGGTTAATTCCGTTAACGAACGAGACCTTAACCTGCTAAATAGGATGTGAGA
TTCGGTCTCACCATCGCTTCTTAGAGGGACTTTGCGGTCATAAATCGCAAGGAAGTTTAAGGCAATA
ACAGGTCTGTGATGCCCTTAGATGTCCTGGGCTGCACGCGCGCTACACTGATGCATTCACTGAGTG
TATCCTTGGCTGAGAGGCCTGGGTAACCTTGAGTATGCATCGTGATGGGGATTGATTATTGTAATTCT
TAATCATGAACGAGGAATGCCTAGTATGCGCAAGTCATCAGCTTGTGCAGACTACGTCCCTGCCCT
TTGTACACACCGCCCGTCGCTCCTACCGATCGAGTGATCCG

>KM046918_Switzerland

ATGGCTCATTACAACAGTTATAGTTTATTTGATGTTGGTTTTTACATGGATAACCGTGCTAATTGTAGGG
CTAATACATGTTTGGGCACAGTTGCATTTATTAGACCTAAAACCTCCCCGCTTTTGCGGTGTTTCGGT
GATTCATAATAAATTAGCGAATCGCATGGCTTTGCTGGCGATGTATCATTCAAGTTTCTGACCTATCAG
CTTTGGACGGTAGGGTATTGGCCTACCGGGGCAACGACGGGTAACGGGGAATTAGGGTTCGATTC
CGGAGAGGGAGCCTGAGAAACGGCTACCACATCTAAGGAAGGCAGCAGGCGCGCAAATTACCCA
ATCCTGACACAGGGAGGTAGTGACAAGAAATAACAATACGGGGCTTGAAGTCTTGTAATTGGAATGA
TGGGAATTTAAACCCCTTCCAGAGTATCAATTGGAGGGCAAGTCTGGTGCCAGCAGCCGCGGTAAT
TCCAGCTCCAATAGCGTATATTAAACTTGTTGCAGTTAAAAAGCTCGTAGTTGAATTTCTGCTGCATCG
TGGTTCTTCGCTATGTCGAGTGATCTTCGTTGTGGCTTAGTTGGGGCATGTTTTCATGACTCGACGTTT
ACTTTGAGAAAATTAGAGTGCTTCAAGCAGGCTTTTGCCTTGAATACTTTAGCATGGAGTAATGGAGTA
GGACTTTGGTTCTATTTTGTTGGTTTTAGGAGCCGGAGTAATGGTTAATAGGAACAGTTGGGGGCATT
CGTATTTGACTGTCAGAGGTGAAATTCTTAGATTTGTCAAAGACGAACTACTGCGAAAGCATTTGCCA
AGGATGTTTCCATTAATCAAGAACGAAAGTTAGGGGATCGAAGACGATCAGATACCGTCGTAGTCCT
AACCATGAACGATGCCGACTAGAGATTGGAGGTCGTCAGTTTGAACGACTCCTTCAGCACCTTGAG
AGAAATCAAAGTCTTTGGGTTCTGGGGGGAGTATGGTCGCAAGGCTGAAACTTAAAGGAATTGACG
GAAGGGCACCACCAGGCGTGGAGCCTGCGGCTTAATTTGACTCAACACGGGGAAACTCACCAGG
TCCAGACAGAGGAAGGATTGACAGATTGATAGCTCTTTCTTGATTCTTTGGGTGGTGGTGCATGGCC
GCTCTTAGTTGGTGGAGTGATTTGTCTGGTTAATTCCGTTAACGAACGAGACCTTAACCTGCTAAATA
GGATGCGAGATTTGGTCTCGTTATCGCTTCTTAGAGGGACTTTGCGGTCATAAATCGCAAGGAAGTTT
AAGGCAATAACAGGTCTGTGATGCCCTTAGATGTCCTGGGCTGCACGCGCGCTACACTGATGCATT
CACTGAGTGTATCCTTGGCTGAAAGGCTCGGGTAATCTTGAGTATGCATCGTGATGGGGATTGATTAT
TGTAATTCTTAATCATGAACGAGGAATGCCTAGTATGCGCAAGTCATCAGCTTGTGCAGACTACGTC
CCTGCCCTTTGTACACACCGCCCGTCGCTCCTACCGATCGGGTGATCCG

>KM046919_Switzerland

ATGGCTCATTACAACAGTTATAGTTTATTTGATGTTGGTTTTTACATGGATAACCGTGCTAATTGTAGGG
CTAATACATGTTTGGGCACAGTTGCATTTATTAGACCTAAAACCTCCCCGCTTTTGCGGTGTTTCGGT
GATTCATAATAAATTAGCGAACCGCATGGCTTTGCTGGCGATGTATCATTCAAGTTTCTGACCTATCA
GCTTTGGACGGTAGGGTATTGGCCTACCGGGGCAACGACGGGTAACGGGGAATTAGGGTCCGATT
CCGGAGAGGGAGCCTGAGAAACGGCTACCACATCTAAGGAAGGCAGCAGGCGCGCAAATTACC
CAATCCTGACACAGGGAGGTAGTGACAAGAAATAACAATACGGGGCTTGAAGTCTTGTAATTGGAAT
GATGGGAATTTAAACCCCTTCCAGAGTATCAATTGGAGGGCAAGTCTGGTGCCAGCAGCCGCGGT
AATTCCAGCTCCAATAGCGTATATTAAACTTGTTGCAGTTAAAAAGCTCGTAGTTGAATTTCTGCTGCA

TCGTGGTTCTTCGCTATGTCGAGTGATCTTCGTTGTGGCTTAGTTGGGGCATGTTTTCATGACTCGAC
GTTTACTTTGAGAAAATTAGAGTGCTTCAAGCAGGCTTTTGCCTTGAATACTTTAGCATGGAATAATGG
AGTAGGACTTTGGTTCTATTTTGTTGGTTTTAGGAGCCGGAGTAATGGTTAATAGGAACAGTTGGGGG
CATTCGTATTTGACTGTCAGAGGTGAAATTCTTAGATTTGTCAAAGACGAACTACTGCGAAAGCATTTG
CCAAGGATGTTTCCATTAATCAAGAACGAAAGTTAGGGGATCGAAGACGATCAGATACCGTCGTAGT
CCTAACCATAAACGATGCCGACTAGAGATTGGAGGTCGTCAGTTTGAACGGCTCCTTCAGCACCTT
GAGAGAAATCAAAGTCTTTGGGTTCTGGGGGGAGTATGGTCGCAAGGCTGAAACTTAAAGGAATTGA
CGGAAGGGCACCACCAGGCGTGGAGCCTGCGGCTTAATTTGACTCAACACGGGGAAACTCACCA
GGTCCAGACAGAGGAAGGATTGACAGATTGATAGCTCTTTCTTGATTCTTTGGGTGGTGGTGCATGG
CCGTTCTTAGTTGGTGGAGTGATTTGTCTGGTTAATTCCGTTAACGAACGAGACCTTAACCTGCTAAA
TAGGATGCGAGATTTGGTCTCGTTATCGCTTCTTAGAGGGACTTTGCGGTCATAAATCACAAGGAAGT
TTAAGGCAATAACAGGTCTGTGATGCCCTTAGATGTCCTGGGCTGCACGCGCGCTACACTGATGCA
TTCACTGAGTGTATCCTTGGCTGAAAGGCTCGGGTAATCTTGAGTATGCATCGTGATGGGGATTGATT
ATTGTAATTCTTAATCATGAACGAGGAATGCCTAGTATGCGCAAGTCATCAGCTTGTGCAGACTACGT
CCCTGCCCTTTGTACACACCGCCCGTCGCTCCTACCGATCGAGTGATCCG

>KM046920_Switzerland

ATGGCTCATTACAACAGTTATAGTTTATTTGATGTTGGTCTTTACATGGATAACCGTGCTAATTGTAGGG
CTAATACATGTTTGGGCACAGTTGCATTTATTAGACCTAAAACCTCCCCGCTTTTGCGGTGTTTCGGT
GATTCATAATAAATTAGCGAATCGCATGGCTTTGCTGACGATGTATCATTCAAGTTTCTGACCTATCAG
CTTTGGACGGTAGGGTATTGGCCTACCGGGGCAACGACGGGTAACGGGGAATTAGGGTTCGATTC
CGGAGAGGGAGCCTGAGAAACGGCTACCACATCTAAGGAAGGCAGCAGGCGCGCAAATTACCCA
ATCCTGACACAGGGAGGTAGTGACAAGAAATAACAATACGGGGCTTGAAGTCTTGTAATTGGAATGA
TGGGAATTTAAACCCCTTCCAGAGTATCAATTGGAGGGCAAGTCTGGTGCCAGCAGCCGCGGTAAT
TCCAGCTCCAATAGCGTATATTAAACTTGATGCAGTTAAAAAGCTCGTAGTTGAATTCCTGCTGCATC
GTGGTTCTTCGCTATGTCGAGTGATCTTCGTTGTGGCTTAGTTGGGGCATGTTTTCATGACTCGACGT
TTACTTTGAGAAAATTAGAGTGCTTCAAGCAGGCTTTTGCCTTGAATACTTTAGCACGGAATAATGGAG
TAGGACTTTGGTTCTATTTTGTTGGTTTTAGGAGCCGGAGTAATGGTTAATAGGAACAGTTGGGGGCA
TTCGTATTTGACTGTCAGAGGTGAAATTCTTAGATTTGTCAAAGACGAACTACTGCGAAAGCATTTGC
CAAGGATGTTTCCATTAATCAAGAACGAAAGTTAGGGGATCGAAGACGATCAGATGCCGTCGTAGTC
CTAACCATAAACGATGCCGACTAGAGATTGGAGGTCGTCAGTTTGAACGACTCCTTCAGCACCTTG
AGAGAAATCAAAGTCTTTGGGTTCTGGGGGGAGTATGGTCGCAAGGCTGAAACTTAAAGGAATTAAC
GGAAGGGCACCACCAGGCGTGGAGCCTGCGGCTTAATTTGACTCAACACGGGGAAACTCACCAG
GTCCAGACAGAGGAAGGATTGACAGATTGATAGCTCTTTCTTGATTCTTTGGGTGGTGGTGCATGGC
CGTTCTTAGTTGGTGGAGTGATTTGTCTGGTTAATTCCGTTAACGAACGAGACCTTAACCTGCTAAATA
GGATGCGAGATTTGGTCTCGCTATCGCTTCTTAGAGGGACTTTGCGGTCATAAATCGCAAGGAAGTT
TAAGGCAATAACAGGTCTGTGATGCCCTTAGATGTCCTGGGCTGCACGCGCGCTACACTGATGCAT
TCACTGAGTGTATCCTTGGCTGAAAGGCTCGGGTAATCTTGAGTATGCATCGTGATGGGGATTGATTA
TTGTAATTCTTAATCATGAACGAGGAATGCCTAGTATGCGCAAGTCATCAGCTTGTGCAGACTACGTC
CCTGCCCTCTGTACACACCGCCCGTCGCTCCTACCGATCGAGTGATCCG

>KM046922_Hungary

ATGGCTCATTACAACAGTTATAGTTTATTTGATGTTGGTTTTTACATGGATAACCGTGCTAATTGTAGGG
CTAATACATGTTTGGGCACAGTTGCATTTATTAGACCTAAAACCTCCCCGCTTTTGCGGTGTTTCGGT
GATTCATAATAAATTAGCGAATCGCATGGCTTTGCTGGCGATGTATCATTCAAGTTTCTGACCTATCAG
CTTTGGACGGTAGGGTATTGGCCTACCGGGGCAACGACGGGTAACGGGGAATTAGGGTTCGATTC
CGGAGAGGGAGCCTGAGAAACGGCTACCACATCTAAGGAAGGCAGCAGGCGTGCAAATTACCCA
ATCCTGACACGGGGAGGTAGTGACAATATGTAACAATATACGGCCTTACGTCGTATAATTGGAATGA

GTACAATTTAAATCCCTTAACGAGGAACAATTGGAGGGCAAGTCTGGTGCCAGCAGCCGCGGTAAT
TCCAGCTCCAATAGCGTATATTAAACTTGTTGCAGTTAAAAAGCTCGTAGTTGAATTTCTGCTGCATCG
TGGTTCTTCGCTATGTCGAGTGATCTTCGTTGTGGCTTAGTTGGGGCATGTTTTCATGACTCGACGTTT
ACTTTGAGAAAATTAGAGTGCTTCAAGCAGGCTTTTGCCTTGAATACTTTAGCATAGGATAATGGAGTA
GGACTTTGGTTCTATTTTGTTGGTTTTAGGAGCCGGAGTAATGGTTAATAGGAACAGTTGGGGGCATT
CGTATTTGACTGTCAGAGGTGAAATTCTTAGATTTGTCAAAGACGAACTACTGCGAAAGCATTTGCCA
AGGATGTTTCCATTAATCAAGAACGAAAGTTAGGGGATCGAAGACGATCAGATACCGTCGTAGTCCT
AACCATAAACGATGCCGACTAGAGATTGGAGGTCGTCAGTTTGAACGACTCCTTCAGCACCTTGAG
AGAAATCAAAGTCTTTGGGTTCTGGGGGGAGTATGGTCGCAAGGCTGAAACTTAAAGGAATCGACG
GAAGGGCACCACCAGGCGTGGAGCCTGCGGCTTAATTTGACTCAACACGGGGAAACTCACCAGG
TCCAGACAGAGGAAGGATTGACAGATTGATAGCTCTTTCTTGATTCTTTGGGTGGTGGTGCATGGCC
GTTCTTAGTTGGTGGAGTGATTTGTCTGGTTAATTCCGTTAACGAACGAGACCTTAACCTGCTAAATAG
GATGCGAGATTTGGTCTCGTTATCGCTTCTTAGAGGGACTTTGCGGTCATAAATCGCAAGGAAGTTTA
AGGCAATAACAGGTCTGTGATGCCCTTAGATGTCCTGGGCTGCACGCGCGCTACACTGATGCATTC
ACTGAGTGTATCCTTGGCTGAAAGGCTCGGGTAATCTTGAGTATGCATCGTGATGGGGATTGATTATT
GTAATTCTTAATCATGAACGAGGAATGCCTAGTATGCGCAAGTCATCAGCTTGTGCAGACTACGTCC
CTGCCCTTTGTACACACCGCCCGTCGCTCCTACCGATCGAGTGATCCG

>KU240070_Brazil

ATGGCTCATTACAACAGTTATAGTTTATTTGATGTTAGTTTCTACATGGATAACCGTGCTAATTGTAGGG
CTAATACATGTTTGCTGTCAGTTGCGTTTATTAGACCTAAAACCTCCCCGCTTTTGCGGTGTATCGGT
GATTCATAATAAATTAGCGAATCGCATGGCTTTGCTGGCGATGTATCATTCAAGTTTCTGACCTATCAG
CTTTGGACGGTAGGGTATTGGCCTACCGGGGCAACGACGGGTAACGGGGAATTAGGGTTCGATTC
CGGAGAGGGAGCCTGAGAAACGGCTACCACATCTAAGGAAGGCAGCAGGCGCGCAAATTACCCA
ATCCTGACACAGGGAGGTAGTGACAAGAAATAACAATACGGGGCTTTAAGTCTTGTAATTGGAATGA
TGGGAATTTAAACCTCTTCCAGAGTATCAATTGGAGGGCAAGTCTGGTGCCAGCAGCCGCGGTAAT
TCCAGCTCCAATAGCGTATATTAAACTTGTTGCAGTTAAAAAGCTCGTAGTTGAATTTCTGCTGTATCG
TTATCTTCTGC-TTGACAGTTTGGTATCGTTATGGCTT-GTTGGGTCAC---
TTTGTGTCCCGGCGTTTACTTTGAGAAAATTAGAGTGCTTCAAGCAGGCTTTTGCCTTGAATACTTTAG
CATGGAATAATGGAGTAGGACTTTGGTTCTATTTTGTTGGTTTTAGGAGCCAGAGTAATGGTTAATAGG
AACAGTTGGGGGCATTCGTATTTGACTGTCAGAGGTGAAATTCTTAGATTTGTCAAAGACGAACTACT
GCGAAAGCATTTGCCAAGGATGTTTTCATTAATCAAGAACGAAAGTTAGGGGATCGAAGACGATCAG
ATACCGTCGTAGTCCTAACTATAAACGATGCCGACTAGAGATTGGAGGTCGTCAGTTTGAACGACTC
CTTCAGCACCTTGAGAGAAATCAAAGTCTTTGGGTTCTGGGGGGAGTATGGTCGCAAGGCTGAAAC
TTAAAGGAATTGACGGAAGGGCACCACCAGGCGTGGAGCCTGCGGCTTAATTTGACTCAACACGG
GGAAACTCACCAGGTCCAGACAGAGGAAGGATTGACAGATTGATGGCTCTTTCTTGATTCTTTGGGT
GGTGGTGCATGGCCGTTCTTAGTTGGTGGAGTGATTTGTCTGGTTAATTCCGTTAACGAACGAGACC
TTAACCTGCTAAATAGGGTGTTGGAGTTATGTTCTACACTGCTTCTTAGAGGGACTTTGCGGTCATAAA
TCGCAAGGAAGTTTAAGGCAATAACAGGTCTGTGATGCCCTTAGATGTCCTGGGCTGCACGCGCG
CTACACTGATGCATTCACTGAGTGTATCCTTGGCTGAGAGGCTTGGGTAATCTTGAGTATGCATCGT
GATGGGGATTGATTATTGTAATTCTTAATCATGAACGAGGAATGCCTAGTATGCGCGAGTCATCAGCT
CGTGCAGACTACGTCCCTGCCCTTTGTACACACCGCCCGTCGCTCCTACCGATC----------

>KU240071_Brazil

ATGGCTCATTACAACAGTTATAGTTTATTTGATGTTAGTTTCTACATGGATAACCGTGCTAATTGTAGGG
CTAATACATGTTTGCTGTCAGTTGCGTTTATTAGACCTAAAACCTCCCCGCTTTTGCGGTGTATCGGT
GATTCATAATAAATTAGCGAATCGCATGGCTTTGCTGGCGATGTATCATTCAAGTTTCTGACCTATCAG
CTTTGGACGGTAGGGTATTGGCCTACCGGGGCAACGACGGGTAACGGGGAATTAGGGTTCGATTC

CGGAGAGGGAGCCTGAGAAACGGCTACCACATCTAAGGAAGGCAGCAGGCGCGCAAATTACCCA
ATCCTGACACAGGGAGGTAGTGACAAGAAATAACAATACGGGGCTTTAAGTCTTGTAATTGGAATGA
TGGGAATTTAAACCTCTTCCAGAGTATCAATTGGAGGGCAAGTCTGGTGCCAGCAGCCGCGGTAAT
TCCAGCTCCAATAGCGTATATTAAACTTGTTGCAGTTAAAAAGCTCGTAGTTGAATTTCTGCTGTATCG
TTATCTTCTGC-TTGACAGTTTGGTATCGTTATGGCTT-GTTGGGTCAC---
TTTGTGTCCCGGCGTTTACTTTGAGAAAATTAGAGTGCTTCAAGCAGGCTTTTGCCTTGAATACTTTAG
CATGGAATAATGGAGTAGGACTTTGGTTCTATTTTGTTGGTTTTAGGAGCCAGAGTAATGGTTAATAGG
AACAGTTGGGGGCATTCGTATTTGACTGTCAGAGGTGAAATTCTTAGATTTGTCAAAGACGAACTACT
GCGAAAGCATTTGCCAAGGATGTTTTCATTAATCAAGAACGAAAGTTAGGGGATCGAAGACGATCAG
ATACCGTCGTAGTCCTAACTATAAACGATGCCGACTAGAGATTGGAGGTCGTCAGTTTGAACGACTC
CTTCAGCACCTTGAGAGAAATCAAAGTCTTTGGGTTCTGGGGGGAGTATGGTCGCAAGGCTGAAAC
TTAAAGGAATTGACGGAAGGGCACCACCAGGCGTGGAGCCTGCGGCTTAATTTGACTCAACACGG
GGAAACTCACCAGGTCCAGACAGAGGAAGGATTGACAGATTGATGGCTCTTTCTTGATTCTTTGGGT
GGTGGTGCATGGCCGTTCTTAGTTGGTGGAGTGATTTGTCTGGTTAATTCCGTTAACGAACGAGACC
TTAACCTGCTAAATAGGGTGTTGGAGTTATGTTCTACACTGCTTCTTAGAGGGACTTTGCGGTCATAAA
TCGCAAGGAAGTTTAAGGCAATAACAGGTCTGTGATGCCCTTAGATGTCCTGGGCTGCACGCGCG
CTACACTGATGCATTCACTGAGTGTATCCTTGGCTGAGAGGCTTGGGTAATCTTGAGTATGCATCGT
GATGGGGATTGATTATTGTAATTCTTAATCATGAACGAGGAATGCCTAGTATGCGCGAGTCATCAGCT
CGTGCAGACTACGTCCCTGCCCTTTGTACACACCGCCCGTCGCTCCTACCGATCGAGTGAT---

>KU672386_USA

ATGGCTCATTAAAACAGTTATAGTTTATTTGATGTTTGTTTCTACATGGATAACCGTGCTAATTGTAGGG
CTAATACATGCTCGGGCACAGTTGCGTTTATTAGACCTAAAACCTCCCCGCTTTTGCGGTGTTTCGG
TGATTCATAATAAATTAGCGAATCGCATGGCTTTGCCGGCGATGTATCATTCAAGTTTCTGACCTATCA
GCTTTGGACGGTAGGGTATTGGCCTACCGGGGCAACGACGGGTAACGGGGAATTAGGGTTCGATT
CCGGAGAGGGAGCCTGAGAAACGGCTACCACATCTAAGGAAGGCAGCAGGCGCGCAAATTACC
CAATCCTGACACAGGGAGGTAGTGACAAGAAATAACAATACGGGGCTTGAAGTCTTGTAATTGGAAT
GATGGGAATTTAAACCCCTTCCAGAGTATCAATTGGAGGGCAAGTCTGGTGCCAGCAGCCGCGGT
AATTCCAGCTCCAATAGCGTATATTAAACTTGTTGCAGTTAAAAAGCTCGTAGTTGAATTTCTGCTGTTT
CGTTGACT---
GCGTTTGGCGTTTGTCATCGTTGCGGCTTGGTTGGGTTTCGATTTCGTTTCCCGGCGTTTACTTTGAG
AAAATTAGAGTGCTTGAAGCAGGCTTTTGCCTTGAATACTTTAGCATGGAATAACGGAGTAGGACTTT
GGTTCTATTTTGTTGGTTTTAGGAGCCAGAGTAATGGTTAATAGGAACAGTTGGGGGCATTCGTATTTG
ACTGTCAGAGGTGAAATTCTTAGATTTGTCAAAGACGAACTACTGCGAAAGCATTTGCCAAGGATGTT
TTCATTAATCAAGAACGAAAGTTAGGGGATCGAAGACGATCAGATACCGTCGTAGTCCTAACCATAA
ACGATGCCGACTAGAGATTGGAGGTCGTCAGTTTGAACGACTCCTTCGGCACCTTGAGAGAAATCA
AAGTCTTTGGGTTCTGGGGGGAGTATGGTCGCAAGGCTGAAACTTAAAGGAATTGACGGAAGGGCA
CCACCAGGCGTGGAGCCTGCGGCTTAATTTGACTCAACACGGGGAAACTCACCAGGTCCAGACA
GAGGAAGGATTGACAGATTGATGGCTCTTTCTTGATTCTTTGGGTGGTGGTGCATGGCCGTTCTTAGT
TGGTGGAGTGATTTGTCTGGTTAATTCCGTTAACGAACGAGACCTTAACCTGCTAAATAGGGTGTGAG
ACTTGGTTTCATTTCCGCTTCTTAGAGGGACTTTGCGGTCATAAATCGCAAGGAAGTTTAAGGCAATA
ACAGGTCTGTGATGCCCTTAGATGTCCTGGGCTGCACGCGCGCTACACTGATGCATTCACTGAGTG
TATCCTTGGCTGAGAGGCTTGGGTAATCTTGAGTATGCATCGTGATGGGGATTGATTATTGTAATTCTT
AATCATGAACGAGGAATGCCTAGTATGCGCAAGTCATCAGCTTGTGCAGACTACGTCCCTGCCCTT
TGTACACACCGCCCGTCGCTCCTACCGATCGAGTGATCCG

>KX227624_Israel

ATGGCTCATTACAACAGTTATAGTTTATTTGATGTTAGTTTCTACATGGATAACCGTGCTAATTGTAGGG
CTAATACATGCTTGCTGTCAGTTGCGTTTATTAGACCTAAAACCTCCCCGCTTTTGCGGTGTTTCGGT
GATTCATAATAAATTAGCGAATCGCATGGCTTTGCTGGCGATGTATCATTCAAGTTTCTGACCTATCAG
CTTTGGACGGTAGGGTATTGGCCTACCGGGGCAACGACGGGTAACGGGGAATTAGGGTTCGATTC
CGGAGAGGGAGCCTGAGAAACGGCTACCACATCTAAGGAAGGCAGCAGGCGCGCAAATTACCCA
ATCCTGACACAGGGAGGTAGTGACAAGAAATAACAATACGGGGCTTTAAGTCTTGTAATTGGAATGA
TGGGAATTTAAACCTCTTCCAGAGTATCAATTGGAGGGCAAGTCTGGTGCCAGCAGCCGCGGTAAT
TCCAGCTCCAATAGCGTATATTAAACTTGTTGCAGTTAAAAAGCTCGTAGTTGAATTTCTGCTGTATCG
TTTTCCTCTGC-
TTGACAGTTGGATTTCGTTACGGCTTAGTTGGGTTACAGACTTGTTACCCAACGTTTACTTTGAGAAAA
TTAGAGTGCTTCAAGCAGGCTTTTGCCTTGAATACTTTAGCATGGAATAATGGAGTAGGACTTTGGTTC
TATTTTGTTGGTTTTAGGAGCCAGAGTAATGGTTAATAGGAACAGTTGGGGGCATTCGTATTTGACTGT
CAGAGGTGAAATTCTTAGATTTGTCAAAGACGAACTACTGCGAAAGCATTTGCCAAGGATGTTTTCAT
TAATCAAGAACGAAAGTTAGGGGATCGAAGACGATCAGATACCGTCGTAGTCCTAACTATAAACGAT
GCCGACTAGAGATTGGAGGTCGTCAGTTTGAACGACTCCTTCAGCACCTTGAGAGAAATCAAAGTC
TTTGGGTTCTGGGGGGAGTATGGTCGCAAGGCTGAAACTTAAAGGAATTGACGGAAGGGCACCAC
CAGGCGTGGAGCCTGCGGCTTAATTTGACTCAACACGGGGAAACTCACCAGGTCCAGACAGAGG
AAGGATTGACAGATTGATAGCTCTTTCTTGATTCTTTGGGTGGTGGTGCATGGCCGTTCTTAGTTGGTG
GAGTGATTTGTCTGGTTAATTCCGTTAACGAACGAGACCTTAACCTGCTAAATAGGGTGTTGGATTCT
AGTTCTACACTGCTTCTTAGAGGGACTTTGCGGTCATAAATCGCAAGGAAGTTTAAGGCAATAACAG
GTCTGTGATGCCCTTAGATGTCCTGGGCTGCACGCGCGCTACACTGATGCATTCACTGAGTGTATC
CTTGGCTGAGAGGCTTGGGTAATCTTGAGTATGCATCGTGATGGGGATTGATTATTGTAATTCTTAATC
ATGAACGAGGAATGCCTAGTATGCGCAAGTCATCAGCTTGTGCAGACTACGTCCCTGCCCTTTGTA
CACACCGCCCGTCGCTCCTACCGATCGAGTGATCCG

>KX227627_Israel

AGTTATAGTTTATTTGATGTTAGTTTCTACATGGATAACCGTGCTAATTGTAGGGCTAATACATGCTTGC
TGTCAGTTGCGTTTATTAAACCTAAAACCTCCCCGCTTTTGCGGGGGTTCGGGGATTCATAATAAATT
AGCGAATCCCATGGCTTTGCTGGCGATGTATCATTCAAGTTTCTGACCTATCAGCTTTGGACGGTAG
GGTATTGGCCTACCGGGGCAACGACGGGTAACGGGGAATTAGGGTTCGATTCCGGAGAGGGAGC
CTGAGAAACGGCTACCACATCTAAGGAAGGCAGCAGGCGCGCAAATTACCCAATCCTGACACAG
GGAGGTAGTGACAAGAAATAACAATACGGGGCTTTAAGTCTTGTAATTGGAATGATGGGAATTTAAAC
CTCTTCCAGAGTATCAATTGGAGGGCAAGTCTGGTGCCAGCAGCCGCGGTAATTCCAGCTCCAATA
GCGTATATTAAACTTGTTGCAGTTAAAAAGCTCGTAGTTGAATTTCTGCTGTATCGTTTTCCTCTGC-
TTGACAGTTGGATTTCGTTATGGCTTATGTGGGTTACAGCCTTGTTACCCAACGTTTACTTTGAAAAAAT
TAAAGTGCTTCAAGCAGGCTTTTGCCTTGAATACTTTAGCATGGAATAATGGAGTAGGACTTTGGTTCT
ATTTTGTTGGTTTTAGGAGCCAGAGTAATGGGTAAAGGAAACAGTTGGGGGCATTCGTATTTGACTGT
CAGAGGTGAATTTCTTAGATTTGTCAAAGACCAACTACTGCGAAAGCATTTGCCAAGGATGTTTTCATT
AATCAAGAACGAAAGTTAGGGGATCGAAGACGATCAGATACCGTCGTAGTCCTAACTATAAACGATG
CCGACTAGAGATTGGAGGTCGTCAGTTTGAACGACTCCTTCAGCACCTTGAGAGAAATCAAAGTCTT
TGGGTTCTGGGGGGAGTATGGTCGCAAGGCTGAAACTTAAAGGAATTGACGGAAGGGCACCACCA
GGCGTGGAGCCTGCGGCTTAATTTGACTCAACACGGGGAAACTCACCAGGTCCAGACAGAGGAA
GGATTGACAGATTGATAGCTCTTTCTTGATTCTTTGGGTGGTGGTGCATGGCCGTTCTTAGTTGGTGG
AGTGATTTGTCTGGTTAATTCCGTTAACGAACGAGACCTTAACCTGCTAAATAGGGTGTTGGATTCTA
GTTCTACACTGCTTCTTAGAGGGACTTTGCGGTCATAAATCGCAAGGAAGTTTAAGGCAATAACAGGT
CTGTGATGCCCTTAGATGTCCTGGGCTGCACGCGCGCTACACTGATGCATTCACTGAGTGTATCCT
TGGCTGAGAGGCTTGGGTAATCTTGAGTATGCATCGTGATGGGGATTGATTATTGTAATTCTTAATCAT

GAACGAGGAATGCCTAGTATGCGCAAGTCATCAGCTTGTGCAGACTACGTCCCTGCCCTTTGTACA
CACCGCCCGTCGCTCCTACCGATCGAGTGATCCG

>KX227636_Israel

--------------TATTCCTGGTTCAAGGGGTGAGGAGTGGTCCA-----
GGCCGAGTGATCCGGTGAATTATTCCATGCTTGCTGTCAGTTGCGTTTATTAGACCTAAAACCTCCC
CGCTTTTGCGGTGTTTCGGTGATTCATAATAAATTAGCGAATCGCATGGCTTTGCTGGCGATGTATCA
TTCAAGTTTCTGACCTATCAGCTTTGGACGGTAGGGTATTGGCCTACCGGGGCAACGACGGGTAAC
GGGGAATTAGGGTTCGATTCCGGAGAGGGAGCCTGAGAAACGGCTACCACATCTAAGGAAGGCA
GCAGGCGCGCAAATTACCCAATCCTGACACAGGGAGGTAGTGACAAGAAATAACAATACGGGGCT
TTAAGTCTTGTAATTGGAATGATGGGAATTTAAACCTCTTCCAGAGTATCAATTGGAGGGCAAGTCTG
GTGCCAGCAGCCGCGGTAATTCCAGCTCCAATAGCGTATATTAAACTTGTTGCAGTTAAAAAGCTCG
TAGTTGAATTTCTGCTGTATCGTTTTCCTCTGC-
TTGACAGTTGGATTTCGTTACGGCTTAGTTGGGTTACAGACTTGTTACCCAACGTTTACTTTGAGAAAA
TTAGAGTGCTTCAAGCAGGCTTTTGCCTTGAATACTTTAGCATGGAATAATGGAGTAGGACTTTGGTTC
TATTTTGTTGGTTTTAGGAGCCAGAGTAATGGTTAATAGGAACAGTTGGGGGCATTCGTATTTGACTGT
CAGAGGTGAAATTCTTAGATTTGTCAAAGACGAACTACTGCGAAAGCATTTGCCAAGGATGTTTTCAT
TAATCAAGAACGAAAGTTAGGGGATCGAAGACGATCAGATACCGTCGTAGTCCTAACTATAAACGAT
GCCGACTAGAGATTGGAGGTCGTCAGTTTGAACGACTCCTTCAGCACCTTGAGAGAAATCAAAGTC
TTTGGGTTCTGGGGGGAGTATGGTCGCAAGGCTGAAACTTAAAGGAATTGACGGAAGGGCACCAC
CAGGCGTGGAGCCTGCGGCTTAATTTGACTCACACGGGGAAAACTCACCAGGTCCAGACAGAGG
AAGGATTGACAGATTGATAGCTCTTTCTTGATTCTTTGGGTGGTGGTGCATGGCCGTTCTTAGTTGGTG
GAGTGATTTGTCTGGTTAATTCCGTTAACGAACGAGACCTTAACCTGCTAAATAGGGTGTTGGATTCT
AGTTCTACACTGCTTCTTAGAGGGACTTTGCGGTCATAAATCGCAAGGAAGTTTAAGGCAATAACAG
GTCTGTGATGCCCTTAGATGTCCTGGGCTGCACGCGCGCTACACTGATGCATTCACTGAGTGTATC
CTTGGCTGAGAGGCTTGGGTAATCTTGAGTATGCATCGTGATGGGGATTGATTATTGTAATTCTTAATC
ATGAACGAGGAATGCCTAGTATGCGCAAGTCATCAGCTTGTGCAGACTACGTCCCTGCCCTTTGTA
CACACCGCCCGTCGCTCCTACCGATCGAGTGATCCG

>KX227638_Israel

TTATAGTTTATTTGATGTTTGTTTCTACATGGATAACCGTGCTAATTGTAGGGCTAATACATGCTCGGGC
ACAGTTGCGTTTATTAGACCTAAAACCTCCCCGCTTTTGCGGTGTTTCGGTGATTCATAATAAATTAGC
GAATCGCATGGCTTTGCCGGCGATGTATCATTCAAGTTTCTGACCTATCAGCTTTGGACGGTAGGGT
ATTGGCCTACCGGGGCAACGACGGGTAACGGGGAATTAGGGTTCGATTCCGGAGAGGGAGCCTG
AGAAACGGCTACCACATCTAAGGAAGGCAGCAGGCGCGCAAATTACCCAATCCTGACACAGGGA
GGTAGTGACAAGAAATAACAATACGGGGCTTGAAGTCTTGTAATTGGAATGATGGGAATTTAAACCC
CTTCCAGAGTATCAATTGGAGGGCAAGTCTGGTGCCAGCAGCCGCGGTAATTCCAGCTCCAATAG
CGTATATTAAACTTGTTGCAGTTAAAAAGCTCGTAGTTGAATTTCTGCTGTTTCGTTGACT---
GCGTTTGGCGTTTGTCATCGTTGCGGCTTGGTTGGGTTTCGATTTCGTTTCCCGGCGTTTACTTTGAG
AAAATTAGAGTGCTTGAAGCAGGCTTTTGCCTTGAATACTTTAGCATGGAATAACGGAGTAGGACTTT
GGTTCTATTTTGTTGGTTTTAGGAGCCAGAGTAATGGTTAATAGGAACAGTTGGGGGCATTCGTATTTG
ACTGTCAGAGGTGAAATTCTTAGATTTGTCAAAGACGAACTACTGCGAAAGCATTTGCCAAGGATGTT
TTCATTAATCAAGAACGAAAGTTAGGGGATCGAAGACGATCAGATACCGTCGTAGTCCTAACCATAA
ACGATGCCGACTAGAGATTGGAGGTCGTCAGTTTGAACGACTCCTTCAGCACCTTGAGAGAAATCA
AAGTCTTTGGGTTCTGGGGGGAGTATGGTCGCAAGGCTGAAACTTAAAGGAATTGACGGAAGGGCA
CCACCAGGCGTGGAGCCTGCGGCTTAATTTGACTCAACACGGGGAAACTCACCAGGTCCAGACA
GAGGAAGGATTGACAGATTGATGGCTCTTTCTTGATTCTTTGGGTGGTGGTGCATGGCCGTTCTTAGT

TGGTGGAGTGATTTGTCTGGTTAATTCCGTTAACGAACGAGACCTTAACCTGCTAAATAGGGTGTGAG
ACTTGGTTTCATTTCCGCTTCTTAGAGGGACTTTGCGGTCATAAATCGCAAGGAAGTTTAAGGCAATA
ACAGGTCTGTGATGCCCTTAGATGTCCTGGGCTGCACGCGCGCTACACTGATGCATTCACTGAGTG
TATCCTTGGCTGAGAGGCTTGGGTAATCTTGAGTATGCATCGTGATGGGGATTGATTATTGTAATTCTT
AATCATGAACGAGGAATGCCTAGTATGCGCAAGTCATCAGCTTGTGCAGACTACGTCCCTGCCCTT
TGTACACACCGCCCGTCGCTCCTACCGATCGAGTGATCCG

>KX722513_Brazil

ATGGCTCATTACAACAGTTATAGTTTATTTGATGTTAGTTTCTACATGGATAACCGTGCTAATTGTAGGG
CTAATACATGTTTGCTGTCAGTTGCGTTTATTAGACCTAAAACCTCCCCGCTTTTGCGGTGTATCGGT
GATTCATAATAAATTAGCGAATCGCATGGCTTTGCTGGCGATGTATCATTCAAGTTTCTGACCTATCAG
CTTTGGACGGTAGGGTATTGGCCTACCGGGGCAACGACGGGTAACGGGGAATTAGGGTTCGATTC
CGGAGAGGGAGCCTGAGAAACGGCTACCACATCTAAGGAAGGCAGCAGGCGCGCAAATTACCCA
ATCCTGACACAGGGAGGTAGTGACAAGAAATAACAATACGGGGCTTTAAGTCTTGTAATTGGAATGA
TGGGAATTTAAACCTCTTCCAGAGTATCAATTGGAGGGCAAGTCTGGTGCCAGCAGCCGCGGTAAT
TCCAGCTCCAATAGCGTATATTAAACTTGTTGCAGTTAAAAGGCTCGTAGTTGAATTTCTGCTGTATCG
TTATCTTCTGC-TTGACAGTTTGGTATCGTTATGGCTT-GTTGGGTCAC---
TTTGTGTCCCGGCGTTTACTTTGAGAAAATTAGAGTGCTTCAAGCAGGCTTTTGCCTTGAATACTTTAG
CATGGAATAATGGAGTAGGACTTTGGTTCTATTTTGTTGGTTTTAGGAGCCAGAGTAATGGTTAATAGG
AACAGTTGGGGGCATTCGTATTTGACTGTCAGAGGTGAAATTCTTAGATTTGTCAAAGACGAACTACT
GCGAAAGCATTTGCCAAGGATGTTTTCATTAATCAAGAACGAAAGTTAGGGGATCGAAGACGATCAG
ATACCGTCGTAGTCCTAACTATAAACGATGCCGACTAGAGATTGGAGGTCGTCAGTTTGAACGACTC
CTTCAGCACCTTGAGAGAAATCAAAGTCTTTGGGTTCTGGGGGGAGTATGGTCGCAAGGCTGAAAC
TTAAAGGAATTGACGGAAGGGCACCACCAGGCGTGGAGCCTGCGGCTTAATTTGACTCAACACGG
GGAAACACACCAGGTCCAGACAGAGGAAGGATTGACAGATTGATGGCTCTTTCTTGATTCTTTGGGT
GGTGGTGCATGGCCGTTCTTAGTTGGTGGAGTGATTTGTCTGGTTAATTCCGTTAACGAACGAGACC
TTAACCTGCTAAATAGGGTGTTGGAGTTATGTTCTACACTGCTTCTTAGAGGGACTTTGCGGTCATAAA
TCGCAAGGAAGTTTAAGGCAATAACAGGTCTGTGATGCCCTTAGATGTCCTGGGCTGCACGCGCG
CTACACTGATGCATTCACTGAGTGTATCCTTGGCTGAGAGGCTTGGGTAATCTTGAGTATGCATCGT
GATGGGGATTGATTATTGTAATTCTTAATCATGAACGAGGAATGCCTAGTATGCGCGAGTCATCAGCT
CGTGCAGACTACGTCCCTGCCCTTTGTACACACCGCCCGTCGCTCCTACCGATCGAGTGATCCG

>KY111760_Cuba

ATGGCTCATTACAACAGTTATAGTTTATTTGATGTTAGTTTCTACATGGATAACCGTGCTAATTGTAGGG
CTAATACATGTTTGCTGTCAGTTGCGTTTATTAGACCTAAAACCTCCCCGCTTTTGCGGTGTATCGGT
GATTCATAATAAATTAGCGAATCGCATGGCTTTGCTGGCGATGTATCATTCAAGTTTCTGACCTATCAG
CTTTGGACGGTAGGGTATTGGCCTACCGGGGCAACGACGGGTAACGGGGAATTAGGGTTCGATTC
CGGAGAGGGAGCCTGAGAAACGGCTACCACATCTAAGGAAGGCAGCAGGCGCGCAAATTACCCA
ATCCTGACACAGGGAGGTAGTGACAAGAAATAACAATACGGGGCTTTAAGTCTTGTAATTGGAATGA
TGGGAATTTAAACCTCTTCCAGAGTATCAATTGGAGGGCAAGTCTGGTGCCAGCAGCCGCGGTAAT
TCCAGCTCCAATAGCGTATATTAAACTTGTTGCAGTTAAAAAGCTCGTAGTTGAATTTCTGCTGTATCG
TTATCTTCTGC-TTGACAGTTTGGTATCGTTATGGCTT-GTTGGGTCAC---
TTTGTGTCCCGGCGTTTACTTTGAGAAAATTAGAGTGCTTCAAGCAGGCTTTTGCCTCGAATACTTTAG
CATGGAATAATGGAGTAGGACTTTGGTTCTATTTTGTTGGTTTTAGGAGCCAGAGTAATGGTTAATAGG
AACAGTTGGGGGCATTCGTATTTGACTGTCAGAGGTGAAATTCTTAGATTTGTCAAAGACGAACTACT
GCGAAAGCATTTGCCAAGGATGTTTTCATTAATCAAGAACGAAAGTTAGGGGATCGAAGACGATCAG
ATACCGTCGTAGTCCTAACTATAAACGATGCCGACTAGAGATTGGAGGTCGTCAGTTTGAACGACTC
CTTCAGCACCTTGAGAGAAATCAAAGTCTTTGGGTTCTGGGGGGAGTATGGTCGCAAGGCTGAAAC

TTAAAGGAATTGACGGAAGGGCACCACCAGGCGTGGAGCCTGCGGCTTAATTTGACTCAACACGG
GGAAACTCACCAGGTCCAGACAGAGGAAGGATTGACAGATTGATGGCTCTTTCTTGATTCTTTGGGT
GGTGGTGCATGGCCGTTCTTAGTTGGTGGAGTGATTTGTCTGGTTAATTCCGTTAACGAACGAGACC
TTAACCTGCTAAATAGGGTGTTGGAGTTATGTTCTACACTGCTTCTTAGAGGGACTTTGCGGTCATAAA
TCGCAAGGAAGTTTAAGGCAATAACAGGTCTGTGATGCCCTTAGATGTCCTGGGCTGCACGCGCG
CTACACTGATGCATTCACTGAGTGTATCCTTGGCTGAGAGGCTTGGGTAATCTTGAGTATGCATCGT
GATGGGGATTGATTATTGTAATTCTTAATCATGAACGAGGAATGCCTAGTATGCGCGAGTCATCAGCT
CGTGCAGACTACGTCCCTGCCCTTTGTACACACCGCCCGTCGCTCCTACCGATCGAGTGATCCG

>KY111762_Cuba

ATGGCTCATTAAAACAGTTATAGTTTATTTGATGTTTGTTTCTACATGGATAACCGTGCTAATTGTAGGG
CTAATACATGCTCGGGCACAGTTGCGTTTATTAGACCTAAAACCTCCCCGCTTTTGCGGTGTTTCGG
TGATTCATAATAAATTAGCGGATCGCATGGCTTTGCCGGCGATGTATCATTCAAGTTTCTGACCTATCA
GCTTTGGACGGTAGGGTATTGGCCTACCGGGGCAACGACGGGTAACGGGGAATTAGGGTTCGATT
CCGGAGAGGGAGCCTGAGAAACGGCTACCACATCTAAGGAAGGCAGCAGGCGCGCAAATTACC
CAATCCTGACACAGGGAGGTAGTGACAAGAAATAACAATACGGGGCTTGAAGTCTTGTAATTGGAAT
GATGGGAATCTAAACCCCTTCCAGAGTATCAATTGGAGGGCAAGTCTGGTGCCAGCAGCCGCGGT
AATTCCAGCTCCAATAGCGTATATTAAACTTGTTGCAGTTAAAAAGCTCGTAGTTGAATTTCTGCTGTTT
CGTTGACT---
GCGTTTGGCGTTTGTCATCGTTGCGGCTTGGTTGGGTTTCGATTTCGTTTCCCGGCGTTTACTTTGAG
AAAATTAGAGTGCTTGAAGCAGGCTTTTGCCTTGAATACTTTAGCATGGAATAACGGAGTAGGACTTT
GGTTCTATTTTGTTGGTTTTAGGAGCCAGAGTAATGGTTAATAGGAACAGTTGGGGGCATTCGTATTTG
ACTGTCAGAGGTGAAATTCTTAGATTTGTCAAAGACGAACTACTGCGAAAGCATTTGCCAAGGATGTT
TTCATTAATCAAGAACGAAAGTTAGGGGATCGAAGACGATCAGATACCGTCGTAGTCCTAACCATAA
ACGATGCCGACTAGAGATTGGAGGTCGTCAGTTTGAACGACTCCTTCAGCACCTTGAGAGAAATCA
AAGTCTTTGGGTTCTGGGGGGAGTATGGTCGCAAGGCTGAAACTTAAAGGAATTGACGGAAGGGCA
CCACCAGGCGTGGAGCCTGCGGCTTAATTTGACTCAACACGGGGAAACTCACCAGGTCCAGACA
GAGGAAGGATTGACAGATTGATGGCTCTTTCTTGATTCTTTGGGTGGTGGTGCATGGCCGTTCTTAGT
TGGTGGAGTGATTTGTCTGGTTAATTCCGTTAACGAACGAGACCTTAACCTGCTAAATAGGGTGTGAG
ACTTGGTTTCATTTCCGCTTCTTAGAGGGACTTTGCGGTCATAAATCGCAAGGAAGTTTAAGGCAATA
ACAGGTCTGTGATGCCCTTAGATGTCCTGGGCTGCACGCGCGCTACACTGATGCATTCACTGAGTG
TATCCTTGGCTGAGAGGCTTGGGTAATCTTGAGTATGCATCGTGATGGGGATTGATTATTGTAATTCTT
AATCATGAACGAGGAATGCCTAGTATGCGCAAGTCATCAGCTTGTGCAGACTACGTCCCTGCCCTT
TGTACACACCGCCCGTCGCTCCTACCGATCGAGTGATCCG

>KY464032_Brazil

ATGGCTCATTAC-
ACAGTTATAGTTTATTTGATGTTAGTTTCTACATGGATAACCGTGCTAATTGTAGGGCTAATACATGTTT
GCTGTCAGTTGCGTTTATTAGACCTAAAACCTCCCCGCTTTTGCGGTGTATCGGTGATTCATAATAAAT
TAGCGAATCGCATGGCTTTGCTGGCGATGTATCATTCAAGTTTCTGACCTATCAGCTTTGGACGGTAG
GGTATTGGCCTACCGGGGCAACGACGGGTAACGGGGAATTAGGGTTCGATTCCGGAGAGGGAGC
CTGAGAAACGGCTACCACATCTAAGGAAGGCAGCAGGCGCGCAAATTACCCAATCCTGACACAG
GGAGGTAGTGACAAGAAATAACAATACGGGGCTTTAAGTCTTGTAATTGGAATGATGGGAATTTAAAC
CTCTTCCAGAGTATCAATTGGAGGGCAAGTCTGGTGCCAGCAGCCGCGGTAATTCCAGCTCCAATA
GCGTATATTAAACTTGTTGCAGTTAAAAAGCTCGTAGTTGAATTTCTGCTGTATCGTTATCTTCTGC-
TTGACAGTTTGGTATCGTTATGGCTT-GTTGGGTCAC---
TTTGTGTCCCGGCGTTTACTTTGAGAAAATTAGAGTGCTTCAAGCAGGCTTTTGCCTTGAATACTTTAG
CATGGAATAATGGAGTAGGACTTTGGTTCTATTTTGTTGGTTTTAGGAGCCAGAGTAATGGTTAATAGG

AACAGTTGGGGGCATTCGTATTTGACTGTCAGAGGTGAAATTCTTAGATTTGTCAAAGACGAACTACT
GCGAAAGCATTTGCCAAGGATGTTTTCATTAATCAAGAACGAAAGTTAGGGGATCGAAGACGATCAG
ATACCGTCGTAGTCCTAACTATAAACGATGCCGACTAGAGATTGGAGGTCGTCAGTTTGAACGACTC
CTTCAGCACCTTGAGAGAAATCAAAGTCTTTGGGTTCTGGGGGGAGTATGGTCGCAAGGCTGAAAC
TTAAAGGAATTGACGGAAGGGCACCACCAGGCGTGGAGCCTGCGGCTTAATTTGACTCAACACGG
GAAAACTCACCAGGTCCAGACAGAGGAAGGATTGACAGATTGATGGCTCTTTCTTGATTCTTTGGGT
GGTGGTGCATGGCCGTTCTTAGTTGGTGGAGTGATTTGTCTGGTTAATTCCGTTAACGAACGAGACC
TTAACCTGCTAAATAGGGTGTTGGAGTTATGTTCTACACTGCTTCTTAGAGGGACTTTGCGGTCATAAA
TCGCAAGGAAGTTTAAGGCAATAACAGGTCTGTGATGCCCTTAGATGTCCTGGGCTGCACGCGCG
CTACACTGATGCATTCACTGAGTGTATCCTTGGCTGAGAGGCTTGGGTAATCTTGAGTATGCATCGT
GATGGGGATTGATTATTGTAATTCTTAATCATGAACGAGGAATGCCTAGTATGCGCGAGTCATCAGCT
CGTGCAGACTACGTCCCTGCCCTTTGTACACACCGCCCGTCGCTCCTACCGATCGAGTGATCAG

>KY464035_Brazil

----CTCA-
TTAAACAGTTATAGTTTATTTGATGTTTGTTTCTACATGGATAACCGTGCTAATTGTAGGGCTAATACATG
CTCGGGCACAGTTGCGTTTATTAGACCTAAAACCTCCCCGCTTTTGCGGTGTTTCGGTGATTCATAAT
AAATTAGCGAATCGCATGGCTTTGCCGGCGATGTATCATTCAAGTTTCTGACCTATCAGCTTTGGACG
GTAGGGTATTGGCCTACCGGGGCAACGACGGGTAACGGGGAATTAGGGTTCGATTCCGGAGAGG
GAGCCTGAGAAACGGCTACCACATCTAAGGAAGGCAGCAGGCGCGCAAATTACCCAATCCTGAC
ACAGGGAGGTAGTGACAAGAAATAACAATACGGGGCTTGAAGTCTTGTAATTGGAATGATGGGAATT
TAAACCCCTTCCAGAGTATCAATTGGAGGGCAAGTCTGGTGCCAGCAGCCGCGGTAATTCCAGCT
CCAATAGCGTATATTAAACTTGTTGCAGTTAAAAAGCTCGTAGTTGAATTTCTGCTGTTTCGTTGACT---
GCGTTTGGCGTTTGTCATCGTTGCGGCTTGGTTGGGTTTCGATTTCGTTTCCCGGCGTTTACTTTGAG
AAAATTAGAGTGCTTGAAGCAGGCTTTTGCCTTGAATACTTTAGCATGGAATAACGGAGTAGGACTTT
GGTTCTATTTTGTTGGTTTTAGGAGCCAGAGTAATGGTTAATAGGAACAGTTGGGGGCATTCGTATTTG
ACTGTCAGAGGTGAAATTCTTAGATTTGTCAAAGACGAACTACTGCGAAAGCATTTGCCAAGGATGTT
TTCATTAATCAAGAACGAAAGTTAGGGGATCGAAGACGATCAGATACCGTCGTAGTCCTAACCATAA
ACGATGCCGACTAGAGATTGGAGGTCGTCAGTTTGAACGACTCCTTCAGCACCTTGAGAGAAATCA
AAGTCTTTGGGTTCTGGGGGGAGTATGGTCGCAAGGCTGAAACTTAAAGGAATTGACGGAAGGGCA
CCACCAGGCGTGGAGCCTGCGGCTTAATTTGACTCAACACGGGGAAACTCACCAGGTCCAGACA
GAGGAAGGATTGACAGATTGATGGCTCTTTCTTGATTCTTTGGGTGGTGGTGCATGGCCGTTCTTAGT
TGGTGGAGTGATTTGTCTGGTTAATTCCGTTAACGAACGAGACCTTAACCTGCTAAATAGGGTGTGG
GAGTTGTGTTCTATTCTGCTTCTTAGAGGGACTTTGCGGTCATAAATCGCAAGGAAGTTTAAGGCAAT
AACAGGTCTGTGATGCCCTTAGATGTCCTGGGCTGCACGCGCGCTACACTGATGCATTCACTGAGT
GTATCCTTGGCTGAGAGGCTTGGGTAATCTTGAGTATGCATCGTGATGGGGATTGATTATTGTAATTCT
TAATCATGAACGAGGAATGCCTAGTATGCGCGAGTCATCAGCTCGTGCAGACTACGTCCCTGCCCT
TTGTACACACCGCCCGTCGCTCCTACCGATCGAGTGATCAG

>KY952230_Brazil

ATGGCTCATTAAAACAGTTATAGTTTATTTGATGTTTGTTTCTACATGGATAACCGTGCTAATTGTAGGG
CTAATACATGCTCGGGCACAGTTGCGTTTATTAGACCTAAAACCTCCCCGCTTTTGCGGTGTTTCGG
TGATTCATAATAAATTAGCGAATCGCATGGCTTTGCCGGCGATGTATCATTCAAGTTTCTGACCTATCA
GCTTTGGACGGTAGGGTATTGGCCTACCGGGGCAACGACGGGTAACGGGGAATTAGGGTTCGATT
CCGGAGAGGGAGCCTGAGAAACGGCTACCACATCTAAGGAAGGCAGCAGGCGCGCAAATTACC
CAATCCTGACACAGGGAGGTAGTGACAAGAAATAACAATACGGGGCTTGAAGTCTTGTAATTGGAAT
GATGGGAATTTAAACCCCTTCCAGAGTATCAATTGGAGGGCAAGTCTGGTGCCAGCAGCCGCGGT
AATTCCAGCTCCAATAGCGTATATTAAACTTGTTGCAGTTAAAAAGCTCGTAGTTGAATTTCTGCTGTTT

CGTTGACT---
GCGTTTGGCGTTTGTCATCGTTGCGGCTTGGTTGGGTTTCGATTTCGTTTCCCGGCGTTTACTTTGAG
AAAATTAGAGTGCTTGAAGCAGGCTTTTGCCTTGAATACTTTAGCATGGAATAACGGAGTAGGACTTT
GGTTCTATTTTGTTGGTTTTAGGAGCCAGAGTAATGGTTAATAGGAACAGTTGGGGGCATTCGTATTTG
ACTGTCAGAGGTGAAATTCTTAGATTTGTCAAAGACGAACTACTGCGAAAGCATTTGCCAAGGATGTT
TTCATTAATCAAGAACGAAAGTTAGGGGATCGAAGACGATCAGATACCGTCGTAGTCCTAACCATAA
ACGATGCCGACTAGAGATTGGAGGTCGTCAGTTTGAACGACTCCTTCAGCACCTTGAGAGAAATCA
AAGTCTTTGGGTTCTGGGGGGAGTATGGTCGCAAGGCTGAAACTTAAAGGAATTGACGGAAGGGCA
CCACCAGGCGTGGAGCCTGCGGCTTAATTTGACTCAACACGGGGAAACTCACCAGGTCCAGACA
GAGGAAGGATTGACAGATTGATGGCTCTTTCTTGATTCTTTGGGTGGTGGTGCATGGCCGTTCTTAGT
TGGTGGAGTGATTTGTCTGGTTAATTCCGTTAACGAACGAGACCTTAACCTGCTAAATAGGGTGTGAG
ACTTGGTTTCATTTCCGCTTCTTAGAGGGACTTTGCGGTCATAAATCGCAAGGAAGTTTAAGGCAATA
ACAGGTCTGTGATGCCCTTAGATGTCCTGGGCTGCACGCGCGCTACACTGATGCATTCACTGAGTG
TATCCTTGGCTGAGAGGCTTGGGTAATCTTGAGTATGCATCGTGATGGGGATTGATTATTGTAATTCTT
AATCATGAACGAGGAATGCCTAGTATGCGCAAGTCATCAGCTTGTGCAGACTACGTCCCTGCCCTT
TGTACACACCGCCCGTCGCTCCTACCGATCGAGTGATCCG

>LC431546_Saudi Arabia

TACCGTGCTAATTGTAGGGCTAATACATGTTTGGGCACAGTTGCATTTATTAGACCTAAAACCTCCCC
GCTTTTGCGGTGTTTCGGTGATTCATAATAAATTAGCGAATCGCATGGCTTTGCTGGCGATGTATCATT
CAAGTTTCTGACCTATCAGCTTTGGACGGTAGGGTATTGGCCTACCGGGGCAACGACGGGTAACG
GGGAATTAGGGTTCGATTCCGGAGAGGGAGCCTGAGAAACGGCTACCACATCTAAGGAAGGCAG
CAGGCGCGCAAATTACCCAATCCTGACACAGGGAGGTAGTGACAAGAAATAACAATACGGGGCTT
GAAGTCTTGTAATTGGAATGATGGGAATTTAAACCCCTTCCAGAGTATCAATTGGAGGGCAAGTCTG
GTGCCAGCAGCCGCGGTAATTCCAGCTCCAATAGCGTATATTAAACTTGTTGCAGTTAAAAAGCTCG
TAGTTGAATTTCTGCTGCATCGTGGTTCTTCGCTATGTCGAGTGATCTTCGTTGTGGCTTAGTTGGGG
CATGTTTTCATGACTCGACGTTTACTTTGAGAAAATTAGAGTGCTTCAAGCAGGCTTTTGCCTTGAATA
CTTTAGCATGGAATAATGGAGTAGGACTTTGGTTCTATTTTGTTGGTTTTAGGAGCCGGAGTAATGGTT
AATAGGAACAGTTGGGGGCATTCGTATTTGACTGTCAGAGGTGAAATTCTTAGATTTGTCAAAGACGA
ACTACTGCGAAAGCATTTGCCAAGGATGTTTCCATTAATCAAGAACGAAAGTTAGGGGATCGAAGAC
GATCAGATACCGTCGTAGTCCTAACCATAAACGATGCCGACTAGAGATTGGAGGTCGTCAGTTTGAA
CGACTCCTTCAGCACCTTGAGAGAAATCAAAGTCTTTGGGTTCTGGGGGGAGTATGGTCGCAAGGC
TGAAACTTAAAGGAATTGACGGAAGGGCACCACCAGGCGTGGAGCCTGCGGCTTAATTTGACTCAA
CACGGGGAAACTCACCAGGTCCAGACAGAGGAAGGATTGACAGATTGATAGCTCTTTCTTGATTCTT
TGGGTGGTGGTGCATGGCCGTTCTTAGTTGGTGGAGTGATTTGTCTGGTTAATTCCGTTAACGAACGA
GACCTTAACCTGCTAAATAGGATGCGAGATTTGGTCTCGTTATCGCTTCTTAGAGGGACTTTGCGGTC
ATAAATCGCAAGGAAGTTTAAGGCAATAACAGGTCTGTGATGCCCTTAGATGTCCTGGGCTGCACG
CGCGCTACACTGATGCATTCACTGAGTGTATCCTTGGCTGAAAGGCTCGGGTAATCTTGAGTATGCA
TCGTGATGGGGATTGATTATTGTAATTCTTAATCATGAACGAGGAATGCCTAGTATGCGCAAGTCATC
AGCTTGTGCAGACTACGTCCCTGCCC-TTGTACACACCGCCCGTCGCTC-------------------

>LC781882_Saudi Arabia

ATGGCTCATTACAACAGTTATAGTTTATTTGATGTTGGTTTTTACATGGATAACCGTGCTAATTGTAGGG
CTAATACATGTTTGGGCACAGTTGCATTTATTAGACCTAAAACCTCCCCGCTTTTGCGGTGTTTCGGT
GATTCATAATAAATTAGCGAATCGCATGGCTTTGCTGGCGATGTATCATTCAAGTTTCTGACCTATCAG
CTTTGGACGGTAGGGTATTGGCCTACCGGGGCAACGACGGGTAACGGGGAATTAGGGTTCGATTC
CGGAGAGGGAGCCTGAGAAACGGCTACCACATCTAAGGAAGGCAGCAGGCGCGCAAATTACCCA

ATCCTGACACAGGGAGGTAGTGACAAGAAATAACAATACGGGGCTTGAAGTCTTGTAATTGGAATGA
TGGGAATTTAAACCCCTTCCAGAGTATCAATTGGAGGGCAAGTCTGGTGCCAGCAGCCGCGGTAAT
TCCAGCTCCAATAGCGTATATTAAACTTGTTGCAGTTAAAAAGCTCGTAGTTGAATTTCTGCTGCATCG
TGGTTCTTCGCTATGTCGAGTGATCTTCGTTGTGGCTTAGTTGGGGCATGTTTTCATGACTCGACGTTT
ACTTTGAGAAAATTAGAGTGCTTCAAGCAGGCTTTTGCCTTGAATACTTTAGCATGGAATAATGGAGTA
GGACTTTGGTTCTATTTTGTTGGTTTTAGGAGCCGGAGTAATGGTTAATAGGAACAGTTGGGGGCATT
CGTATTTGACTGTCAGAGGTGAAATTCTTAGATTTGTCAAAGACGAACTACTGCGAAAGCATTTGCCA
AGGATGTTTCCATTAATCAAGAACGAAAGTTAGGGGATCGAAGACGATCAGATACCGTCGTAGTCCT
AACCATAAACGATGCCGACTAGAGATTGGAGGTCGTCAGTTTGAACGACTCCTTCAGCACCTTGAG
AGAAATCAAAGTCTTTGGGTTCTGGGGGGAGTATGGTCGCAAGGCTGAAACTTAAAGGAATTGACG
GAAGGGCACCACCAGGCGTGGAGCCTGCGGCTTAATTTGACTCAACACGGGGAAACTCACCAGG
TCCAGACAGAGGAAGGATTGACAGATTGATAGCTCTTTCTTGATTCTTTGGGTGGTGGTGCATGGCC
GTTCTTAGTTGGTGGAGTGATTTGTCTGGTTAATTCCGTTAACGAACGAGACCTTAACCTGCTAAATAG
GATGCGAGATTTGGTCTCGTTATCGCTTCTTAGAGGGACTTTGCGGTCATAAATCGCAAGGAAGTTTA
AGGCAATAACAGGTCTGTGATGCCCTTAGATGTCCTGGGCTGCACGCGCGCTACACTGATGCATTC
ACTGAGTGTATCCTTGGCTGAAAGGCTCGGGTAATCTTGAGTATGCATCGTGATGGGGATTGATTATT
GTAATTCTTAATCATGAACGAGGAATGCCTAGTATGCGCAAGTCATCAGCTTGTGCAGACTACGTCC
CTGCCCTTTGTACACACCGCCCGTCGCTCCTACCGATCGAGTGATCCG

>MG052901_Brazil

ATGGCTCATTACAACAGTTATAGTTTATTTGATGTTAGTTTCTACATGGATAACCGTGCTAATTGTAGGG
CTAATACATGTTTGCTGTCAGTTGCGTTTATTAGACCTAAAACCTCCCCGCTTTTGCGGTGTATCGGT
GATTCATAATAAATTAGCGAATCGCATGGCTTTGCTGGCGATGTATCATTCAAGTTTCTGACCTATCAG
CTTTGGACGGTAGGGTATTGGCCTACCGGGGCAACGACGGGTAACGGGGAATTAGGGTTCGATTC
CGGAGAGGGAGCCTGAGAAACGGCTACCACATCTAAGGAAGGCAGCAGGCGCGCAAATTACCCA
ATCCTGACACAGGGAGGTAGTGACAAGAAATAACAATACGGGGCTTTAAGTCTTGTAATTGGAATGA
TGGGAATTTAAACCTCTTCCAGAGTATCAATTGGAGGGCAAGTCTGGTGCCAGCAGCCGCGGTAAT
TCCAGCTCCAATAGCGTATATTAAACTTGTTGCAGTTAAAAAGCTCGTAGTTGAATTTCTGCTGTATCG
TTATCTTCTGC-TTGACAGTTTGGTATCGTTATGGCTT-GTTGGGTCAC---
TTTGTGTCCCGGCGTTTACTTTGAGAAAATTAGAGTGCTTCAAGCAGGCTTTTGCCTTGAATACTTTAG
CATGGAATAATGGAGTAGGACTTTGGTTCTATTTTGTTGGTTTTAGGAGCCAGAGTAATGGTTAATAGG
AACAGTTGGGGGCATTCGTATTTGACTGTCAGAGGTGAAATTCTTAGATTTGTCAAAGACGAACTACT
GCGAAAGCATTTGCCAAGGATGTTTTCATTAATCAAGAACGAAAGTTAGGGGATCGAAGACGATCAG
ATACCGTCGTAGTCCTAACTATAAACGATGCCGACTAGAGATTGGAGGTCGTCAGTTTGAACGACTC
CTTCAGCACCTTGAGAGAAATCAAAGTCTTTGGGTTCTGGGGGGAGTATGGTCGCAAGGCTGAAAC
TTAAAGGAATTGACGGAAGGGCACCACCAGGCGTGGAGCCTGCGGCTTAATTTGACTCAACACGG
GGAAACTCACCAGGTCCAGACAGAGGAAGGATTGACAGATTGATGGCTCTTTCTTGATTCTTTGGGT
GGTGGTGCATGGCCGTTCTTAGTTGGTGGAGTGATTTGTCTGGTTAATTCCGTTAACGAACGAGACC
TTAACCTGCTAAATAGGGTGTTGGAGTTATGTTCTACACTGCTTCTTAGAGGGACTTTGCGGTCATAAA
TCGCAAGGAAGTTTAAGGCAATAACAGGTCTGTGATGCCCTTAGATGTCCTGGGCTGCACGCGCG
CTACACTGATGCATTCACTGAGTGTATCCTTGGCTGAGAGGCTTGGGTAATCTTGAGTATGCATCGT
GATGGGGATTGATTATTGTAATTCTTAATCATGAACGAGGAATGCCTAGTATGCGCGAGTCATCAGCT
CGTGCAGACTACGTCCCTGCCCTTTGTACACACCGCCCGTCGCTCCTACCGATCGAGTGATCC-

>MG569895_Turkey

ATGGCTCATTACAACAGTTATAGTTTATTTGATGTTAGTTTCTACATGGATAACCGTGCTAATTGTAGGG
CTAATACATGCTTGCTGTCAGTTGCGTTTATTAGACCTAAAACCTCCCCGCTTTTGCGGTGTTTCGGT
GATTCATAATAAATTAGCGAATCGCATGGCTTTGCTGGCGATGTATCATTCAAGTTTCTGACCTATCAG

CTTTGGACGGTAGGGTATTGGCCTACCGGGGCAACGACGGGTAACGGGGAATTAGGGTTCGATTC
CGGAGAGGGAGCCTGAGAAACGGCTACCACATCTAAGGAAGGCAGCAGGCGCGCAAATTACCCA
ATCCTGACACAGGGAGGTAGTGACAAGAAATAACAATACGGGGCTTTAAGTCTTGTAATTGGAATGA
TGGGAATTTAAACCTCTTCCAGAGTATCAATTGGAGGGCAAGTCTGGTGCCAGCAGCCGCGGTAAT
TCCAGCTCCAATAGCGTATATTAAACTTGTTGCAGTTAAAAAGCTCGTAGTTGAATTTCTGCTGTATCG
TTTTCCTCTGC-
TTGACAGTTGGATTTCGTTACGGCTTAGTTGGGTTACAGCCTTGTTACCCAACGTTTACTTTGAGAAAA
TTAGAGTGCTTCAAGCAGGCTTTTGCCTTGAATACTTTAGCATGGAATAATGGAGTAGGACTTTGGTTC
TATTTTGTTGGTTTTAGGAGCCAGAGTAATGGTTAATAGGAACAGTTGGGGGCATTCGTATTTGACTGT
CAGAGGTGAAATTCTTAGATTTGTCAAAGACGAACTACTGCGAAAGCATTTGCCAAGGATGTTTTCAT
TAATCAAGAACGAAAGTTAGGGGATCGAAGACGATCAGATACCGTCGTAGTCCTAACTATAAACGAT
GCCGACTAGAGATTGGAGGTCGTCAGTTTGAACGACTCCTTCAGCACCTTGAGAGAAATCAAAGTC
TTTGGGTTCTGGGGGGAGTATGGTCGCAAGGCTGAAACTTAAAGGAATTGACGGAAGGGCACCAC
CAGGCGTGGAGCCTGCGGCTTAATTTGACTCAACACGGGGAAACTCACCAGGTCCAGACAGAGG
AAGGATTGACAGATTGATAGCTCTTTCTTGATTCTTTGGGTGGTGGTGCATGGCCGTTCTTAGTTGGTG
GAGTGATTTGTCTGGTTAATTCCGTTAACGAACGAGACCTTAACCTGCTAAATAGGGTGTTGGATTCT
AGTTCTACACTGCTTCTTAGAGGGACTTTGCGGTCATAAATCGCAAGGAAGTTTAAGGCAATAACAG
GTCTGTGATGCCCTTAGATGTCCTGGGCTGCACGCGCGCTACACTGATGCATTCACTGAGTGTATC
CTTGGCTGAGAGGCTTGGGTAATCTTGAGTATGCATCGTGATGGGGATTGATTATTGTAATTCTTAATC
ATGAACGAGGAATGCCTAGTATGCGCAAGTCATCAGCTTGTGCAGACTACGTCCCTGCCCTTTGTA
CACACCGCCCGTCGCTCCTACCGATCGAGTGATCCG

>MG569900_Turkey

ATGGCTCATTACAACAGTTATAGTTTATTTGATGTTAGTTTCTACATGGATAACCGTGCTAATTGTAGGG
CTAATACATGCTTGCTGTCAGTTGCGTTTATTAGACCTAAAACCTCCCCGCTTTTGCGGTGTTTCGGT
GATTCATAATAAATTAGCGAATCGCATGGCTTTGCTGGCGATGTATCATTCAAGTTTCTGACCTATCAG
CTTTGGACGGTAGGGTATTGGCCTACCGGGGCAACGACGGGTAACGGGGAATTAGGGTTCGATTC
CGGAGAGGGAGCCTGAGAAACGGCTACCACATCTAAGGAAGGCAGCAGGCGCGCAAATTACCCA
ATCCTGACACAGGGAGGTAGTGACAAGAAATAACAATACGGGGCTTTAAGTCTTGTAATTGGAATGA
TGGGAATTTAAACCTCTTCCAGAGTATCAATTGGAGGGCAAGTCTGGTGCCAGCAGCCGCGGTAAT
TCCAGCTCCAATAGCGTATATTAAACTTGTTGCAGTTAAAAAGCTCGTAGTTGAATTTCTGCTGTATCG
TTTTCCTCTGC-
TTGACAGTTGGATTTCGTTACGGCTTAGTTGGGTTACAGCCTTGTTACCCAACGTTTACTTTGAGAAAA
TTAGAGTGCTTCAAGCAGGCTTTTGCCTTGAATACTTTAGCATGGAATAATGGAGTAGGACTTTGGTTC
TATTTTGTTGGTTTTAGGAGCCAGAGTAATGGTTAATAGGAACAGTTGGGGGCATTCGTATTTGACTGT
CAGAGGTGAAATTCTTAGATTTGTCAAAGACGAACTACTGCGAAAGCATTTGCCAAGGATGTTTTCAT
TAATCAAGAACGAAAGTTAGGGGATCGAAGACGATCAGATACCGTCGTAGTCCTAACTATAAACGAT
GCCGACTAGAGATTGGAGGTCGTCAGTTTGAACGACTCCTTCAGCACCTTGAGAGAAATCAAAGTC
TTTGGGTTCTGGGGGGAGTATGGTCGCAAGGCTGAAACTTAAAGGAATTGACGGAAGGGCACCAC
CAGGCGTGGAGCCTGCGGCTTAATTTGACTCAACACGGGGAAACTCACCAGGTCCAGACAGAGG
AAGGATTGACAGATTGATAGCTCTTTCTTGATTCTTTGGGTGGTGGTGCATGGCCGTTCTTAGTTGGTG
GAGTGATTTGTCTGGTTAATTCCGTTAACGAACGAGACCTTAACCTGCTAAATAGGGTGTTGGATTCT
AGTTCTACACTGCTTCTTAGAGGGACTTTGCGGTCATAAATCGCAAGGAAGTTTAAGGCAATAACAG
GTCTGTGATGCCCTTAGATGTCCTGGGCTGCACGCGCGCTACACTGATGCATTCACTGAGTGTATC
CTTGGCTGAGAGGCTTGGGTAATCTTGAGTATGCATCGTGATGGGGATTGATTATTGTAATTCTTAATC
ATGAACGAGGAATGCCTAGTATGCGCAAGTCATCAGCTTGTGCAGACTACGTCCCTGCCCTTTGTA
CACACCGCCCGTCGCTCCTACCGATCGAGTGATCCG

>MG569904_Turkey

ATGGCTCATTAAAACAGTTATAGTTTATTTGATGTTTGTTTCTACATGGATAACCGTGCTAATTGTAGGG
CTAATACATGCTCGGGCACAGTTGCGTTTATTAGACCTAAAACCTCCCCGCTTTTGCGGTGTTTCGG
TGATTCATAATAAATTAGCGAATCGCATGGCTTTGCCGGCGATGTATCATTCAAGTTTCTGACCTATCA
GCTTTGGACGGTAGGGTATTGGCCTACCGGGGCAACGACGGGTAACGGGGAATTAGGGTTCGATT
CCGGAGAGGGAGCCTGAGAAACGGCTACCACATCTAAGGAAGGCAGCAGGCGCGCAAATTACC
CAATCCTGACACAGGGAGGTAGTGACAAGAAATAACAATACGGGGCTTGAAGTCTTGTAATTGGAAT
GATGGGAATTTAAACCCCTTCCAGAGTATCAATTGGAGGGCAAGTCTGGTGCCAGCAGCCGCGGT
AATTCCAGCTCCAATAGCGTATATTAAACTTGTTGCAGTTAAAAAGCTCGTAGTTGAATTTCTGCTGTTT
CGTTGACT---
GCGTTTGGCGTTTGTCATCGTTGCGGCTTGGTTGGGTTTCGATTTCGTTTCCCGGCGTTTACTTTGAG
AAAATTAGAGTGCTTGAAGCAGGCTTTTGCCTTGAATACTTTAGCATGGAATAACGGAGTAGGACTTT
GGTTCTATTTTGTTGGTTTTAGGAGCCAGAGTAATGGTTAATAGGAACAGTTGGGGGCATTCGTATTTG
ACTGTCAGAGGTGAAATTCTTAGATTTGTCAAAGACGAACTACTGCGAAAGCATTTGCCAAGGATGTT
TTCATTAATCAAGAACGAAAGTTAGGGGATCGAAGACGATCAGATACCGTCGTAGTCCTAACCATAA
ACGATGCCGACTAGAGATTGGAGGTCGTCAGTTTGAACGACTCCTTCAGCACCTTGAGAGAAATCA
AAGTCTTTGGGTTCTGGGGGGAGTATGGTCGCAAGGCTGAAACTTAAAGGAATTGACGGAAGGGCA
CCACCAGGCGTGGAGCCTGCGGCTTAATTTGACTCAACACGGGGAAACTCACCAGGTCCAGACA
GAGGAAGGATTGACAGATTGATGGCTCTTTCTTGATTCTTTGGGTGGTGGTGCATGGCCGTTCTTAGT
TGGTGGAGTGATTTGTCTGGTTAATTCCGTTAACGAACGAGACCTTAACCTGCTAAATAGGGTGTGAG
ACTTGGTTTCATTTCCGCTTCTTAGAGGGACTTTGCGGTCATAAATCGCAAGGAAGTTTAAGGCAATA
ACAGGTCTGTGATGCCCTTAGATGTCCTGGGCTGCACGCGCGCTACACTGATGCATTCACTGAGTG
TATCCTTGGCTGAGAGGCTTGGGTAATCTTGAGTATGCATCGTGATGGGGATTGATTATTGTAATTCTT
AATCATGAACGAGGAATGCCTAGTATGCGCAAGTCATCAGCTTGTGCAGACTACGTCCCTGCCCTT
TGTACACACCGCCCGTCGCTCCTACCGATCGAGTGATCCG

>MK392050_Israel

ATGGCTCATTAAAACAGTTATAGTTTATTTGATGTTTGTTTCTACATGGATAACCGTGCTAATTGTAGGG
CTAATACATGCTCGGGCACAGTTGCGTTTATTAGACCTAAAACCTCCCCGCTTTTGCGGTGTTTCGG
TGATTCATAATAAATTAGCGAATCGCATGGCTTTGCCGGCGATGTATCATTCAAGTTTCTGACCTATCA
GCTTTGGACGGTAGGGTATTGGCCTACCGGGGCAACGACGGGTAACGGGGAATTAGGGTTCGATT
CCGGAGAGGGAGCCTGAGAAACGGCTACCACATCTAAGGAAGGCAGCAGGCGCGCAAATTACC
CAATCCTGACACAGGGAGGTAGTGACAAGAAATAACAATACGGGGCTTGAAGTCTTGTAATTGGAAT
GATGGGAATCTAAACCCCTTCCAGAGTATCAATTGGAGGGCAAGTCTGGTGCCAGCAGCCGCGGT
AATTCCAGCTCCAATAGCGTATATTAAACTTGTTGCAGTTAAAAAGCTCGTAGTTGAATTTCTGCTGTTT
CGTTGACT---
GCGTTTGGCGTTTGTCATCGTTGCGGCTTGGTTGGGTTTCGATTTCGTTTCCCGGCGTTTACTTTGAG
AAAATTAGAGTGCTTGAAGCAGGCTTTTGCCTTGAATACTTTAGCATGGAATAACGGAGTAGGACTTT
GGTTCTATTTTGTTGGTTTTAGGAGCCAGAGTAATGGTTAATAGGAACAGTTGGGGGCATTCGTATTTG
ACTGTCAGAGGTGAAATTCTTAGATTTGTCAAAGACGAACTACTGCGAAAGCATTTGCCAAGGATGTT
TTCATTAATCAAGAACGAAAGTTAGGGGATCGAAGACGATCAGATACCGTCGTAGTCCTAACCATAA
ACGATGCCGACTAGAGATTGGAGGTCGTCAGTTTGAACGACTCCTTCAGCACCTTGAGAGAAATCA
AAGTCTTTGGGTTCTGGGGGGAGTATGGTCGCAAGGCTGAAACTTAAAGGAATTGACGGAAGGGCA
CCACCAGGCGTGGAGCCTGCGGCTTAATTTGACTCAACACGGGGAAACTCACCAGGTCCAGACA
GAGGAAGGATTGACAGATTGATGGCTCTTTCTTGATTCTTTGGGTGGTGGTGCATGGCCGTTCTTAGT
TGGTGGAGTGATTTGTCTGGTTAATTCCGTTAACGAACGAGACCTTAACCTGCTAAATAGGGTGTGAG
ACTTGGTTTCATTTCCGCTTCTTAGAGGGACTTTGCGGTCATAAATCGCAAGGAAGTTTAAGGCAATA

ACAGGTCTGTGATGCCCTTAGATGTCCTGGGCTGCACGCGCGCTACACTGATGCATTCACTGAGTG
TATCCTTGGCTGAGAGGCTTGGGTAATCTTGAGTATGCATCGTGATGGGGATTGATTATTGTAATTCTT
AATCATGAACGAGGAATGCCTAGTATGCGCAAGTCATCAGCTTGTGCA--------------------------------
----

>PV688145_VIETNAM

GGGCAGTCTGGTGCCAGCAGCCGCGGTAATTCCAGCTCCAATAGCGTATATTAAACTTGTTGCAGT
TAAAAAGCTCGTAGTTGAATTTCTGCTGCATCGTGGTTCTTCGCTATGTCGAGTGATCTTCGTTGTGG
CTTAGTTGGGGCATGTTTTCATGACTCGACGTTTACTTTGAGAAAATTAGAGTGCTTCAAGCAGGCTTT
TGCCTTGAATACTTTAGCATGGAATAATGGAGTAGGACTTTGGTTCTATTTTGTTGGTTTTAGGAGCCG
GAGTAATGGTTAATAGGAACAGTTGGGGGCATTCGTATTTGACTGTCAGAGGTGAAATTCTTAGATTTG
TCAAAGACGAACTACTGCGAAAGCATTTGCCAAGGATGTTTCCATTAATCAAGAACGAAAGTTAGGG
GATCGAAGACGATCAGATACCGTCGTAGTCCTAACCATAAAC--

>PV688146_VIETNAM

GCAGTCTGGTGCCAGCAGCCGCGGTAATTCCAGCTCCAATAGCGTATATTAAACTTGTTGCAGTTAA
AAAGCTCGTAGTTGAATTTCTGCTGCATCGTGGTTCTTCGCTATGTCGAGTGATCTTCGTTGTGGCTTA
GTTGGGGCATGTTTTCATGACTCGACGTTTACTTTGAGAAAATTAGAGTGCTTCAAGCAGGCTTTTGC
CTTGAATACTTTAGCATGGAATAATGGAGTAGGACTTTGGTTCTATTTTGTTGGTTTTAGGAGCCGGAG
TAATGGTTAATAGGAACAGTTGGGGGCATTCGTATTTGACTGTCAGAGGTGAAATTCTTAGATTTGTCA
AAGACGAACTACTGCGAAAGCATTTGCCAAGGATGTTTCCATTAATCAAGAACGAAAGTTAGGGGAT
CGAAGACGATCAGATACCGTCGTAGTCCTAACCATAAACT---

>PV688147_VIETNAM

GGAGGGCAGTCTGGTGCCAGCAGCCGCGGTAATTCCAGCTCCAATAGCGTATATTAAACTTGTTGC
AGTTAAAAAGCTCGTAGTTGAATTTCTGCTGCATCGTGGTTCTTCGCTATGTCGAGTGATCTTCGTTGT

GGCTTAGTTGGGGCATGTTTTCATGACTCGACGTTTACTTTGAGAAAATTAGAGTGCTTCAAGCAGGC
TTTTGCCTTGAATACTTTAGCATGGAATAATGGAGTAGGACTTTGGTTCTATTTTGTTGGTTTTAGGAGC
CGGAGTAATGGTTAATAGGAACAGTTGGGGGCATTCGTATTTGACTGTCAGAGGTGAAATTCTTAGAT
TTGTCAAAGACGAACTACTGCGAAAGCATTTGCCAAGGATGTTTCCATTAATCAAGAACGAAAGTTAG
GGGATCGAAGACGATCAGATACCGTCGTAGTCCTAACCATAAACT---

>PV688148_VIETNAM

CAGTCTGGTGCCAGCAGCCGCGGTAATTCCAGCTCCAATAGCGTATATNAAACTTGTTGCAGTTAAA
AAGCTCGTAGTTGAATTTCTGCTGCATCGTGGTTCTTCGCTATGTCGAGTGATCTTCGTTGTGGCTTA
GTTGGGGCATGTTTTCATGACTCGACGTTTACTTTGAGAAAATTAGAGTGCTTCAAGCAGGCTTTTGC
CTTGAATACTTTAGCATGGAATAATGGAGTAGGACTTTGGTTCTATTTTGTTGGTTTTAGGAGCCGGAG
TAATGGTTAATAGGAACAGTTGGGGGCATTCGTATTTGACTGTCAGAGGTGAAATTCTTAGATTTGTCA
AAGACGAACTACTGCGAAAGCATTTGCCAAGGATGTTTCCATTAATCAAGAACGAAAGTTAGGGGAT
CGAAGACGATCAGATACCGTCGTAGTCCTAACCATAAACT---

>PV688149_VIETNAM

---TGGTG-CCGCAGCCGCGGT-
ATTCCAGCTCCAATAGCGTATATTAAACTTGTTGCAGTTAAAAAGCTCGTAGTTGAATTTCTGCTGTTTC
GTTGACT---
GCGTTTGGCGTTTGTCATCGTTGCGGCTTGGTTGGGTTTCGATTTCGTTTCCCGGCGTTTACTTTGAG
AAAATTAGAGTGCTTGAAGCAGGCTTTTGCCTTGAATACTTTAGCATGGAATAACGGAGTAGGACTTT
GGTTCTATTTTGTTGGTTTTAGGAGCCAGAGTAATGGTTAATAGGAACAGTTGGGGGCATTCGTATTTG
ACTGTCAGAGGTGAAATTCTTAGATTTGTCAAAGACGAACTACTGCGAAAGCATTTGCCAAGGATGTT
TTCATTAATCAAGAACGAAAGTTAGGGGATCGAAGACGATCAGATACCGTCGTAGTCCTAACCATAA
ACT--

>PV688150_VIETNAM

AGGGCAAGTCTGGTGCCAGCAGCCGCGGTAATTCCAGCTCCAATAGCGTATATTAAACTTGTTGCA
GTTAAAAAGCTCGTAGTTGAATTTCTGCTGTTTCGTTGACT---
GCGTTTGGCGTTTGTCATCGTTGCGGCTTGGTTGGGTTTCGATTTCGTTTCCCGGCGTTTACTTTGAG
AAAATTAGAGTGCTTGAAGCAGGCTTTTGCCTTGAATACTTTAGCATGGAATAACGGAGTAGGACTTT
GGTTCTATTTTGTTGGTTTTAGGAGCCAGAGTAATGGTTAATAGGAACAGTTGGGGGCATTCGTATTTG
ACTGTCAGAGGTGAAATTCTTAGATTTGTCAAAGACGAACTACTGCGAAAGCATTTGCCAAGGATGTT
TTCATTAATCAAGAACGAAAGTTAGGGGATCGAAGACGATCAGATACCGTCGTAGTCCTAACCATAA
ACT--

>PV688151_VIETNAM

GCAAGTCTGGTGCCAGCAGCCGCGGTAATTCCAGCTCCAATAGCGTATATTAAACTTGTTGCAGTTA
AAAAGCTCGTAGTTGAATTTCTGCTGTTTCGTTGACT---
GCGTTTGGCGTTTGTCATCGTTGCGGCTTGGTTGGGTTTCGATTTCGTTTCCCGGCGTTTACTTTGAG
AAAATTAGAGTGCTTGAAGCAGGCTTTTGCCTTGAATACTTTAGCATGGAATAACGGAGTAGGACTTT
GGTTCTATTTTGTTGGTTTTAGGAGCCAGAGTAATGGTTAATAGGAACAGTTGGGGGCATTCGTATTTG
ACTGTCAGAGGTGAAATTCTTAGATTTGTCAAAGACGAACTACTGCGAAAGCATTTGCCAAGGATGTT
TTCATTAATCAAGAACGAAAGTTAGGGGATCGAAGACGATCAGATACCGTCGTAGTCCTAACCATAA
ACTAA---

>PV688152_VIETNAM

GGCAAGTCTGGTGCCAGCAGCCGCGGTAATTCCAGCTCCAATAGCGTATATTAAACTTGTTGCAGTT
AAAAAGCTCGTAGTTGAATTTCTGCTGCATCGTGGTTCTTCGCTATGTCGAGTGATCTTCGTTGTGGC
TTAGTTGGGGCATGTTTTCATGACTCGACGTTTACTTTGAGAAAATTAGAGTGCTTCAAGCAGGCTTTT
GCCTTGAATACTTTAGCATGGAATAATGGAGTAGGACTTTGGTTCTATTTTGTTGGTTTTAGGAGCCGG

AGTAATGGTTAATAGGAACAGTTGGGGGCATTCGTATTTGACTGTCAGAGGTGAAATTCTTAGATTTGT
CAAAGACGAACTACTGCGAAAGCATTTGCCAAGGATGTTTCCATTAATCAAGAACGAAAGTTAGGGG
ATCGAAGACGATCAGATACCGTCGTAGTCCTAACCATAAACTA---

>PV688153_VIETNAM

GGCAAGTCTGGTGCCAGCAGCCGCGGTAATTCCAGCTCCAATAGCGTATATTAAACTTGTTGCAGTT
AAAAAGCTCGTAGTTGAATTTCTGCTGTTTCGTTGACT---
GCGTTTGGCGTTTGTCATCGTTGCGGCTTGGTTGGGTTTCGATTTCGTTTCCCGGCGTTTACTTTGAG
AAAATTAGAGTGCTTGAAGCAGGCTTTTGCCTTGAATACTTTAGCATGGAATAACGGAGTAGGACTTT
GGTTCTATTTTGTTGGTTTTAGGAGCCAGAGTAATGGTTAATAGGAACAGTTGGGGGCATTCGTATTTG
ACTGTCAGAGGTGAAATTCTTAGATTTGTCAAAGACGAACTACTGCGAAAGCATTTGCCAAGGATGTT
TTCATTAATCAAGAACGAAAGTTAGGGGATCGAAGACGATCAGATACCGTCGTAGTCCTAA-----------
----

>PV688154_VIETNAM

GTCTGGTGCCAGCAGCCGCGGTAATTCCAGCTCCAATAGCGTATATNAAACTTGTTGCAGTTAAAAA
GCTCGTAGTTGAATTTCTGCTGCATCGTGGTTCTTCGCTATGTCGAGTGATCTTCGTTGTGGCTTAGTT
GGGGCATGTTTTCATGACTCGACGTTTACTTTGAGAAAATTAGAGTGCTTCAAGCAGGCTTTTGCCTT
GAATACTTTAGCATGGAATAATGGAGTAGGACTTTGGTTCTATTTTGTTGGTTTTAGGAGCCGGAGTAA
TGGTTAATAGGAACAGTTGGGGGCATTCGTATTTGACTGTCAGAGGTGAAATTCTTAGATTTGTCAAAG
ACGAACTACTGNGAAAGCATTTGCCAAGGATGTTTCCATTAATCAAGAACGAAAGTTAGGGGATCGA
AGACGATCAGATACCGTCGTAGTCCTAACCATAAACTA---

>PX369340_VIETNAM

CTGGTGCCAGCAGCCGCGGTAATTCCAGCTCCAATAGCGTATATTAAACTTGTTGCAGTTAAAAAGC
TCGTAGTTGAATTTCTGCTGTATCGTTATCTTCTGC-TTGACAGTTTGGTATCGTTATGGCTT-
GTTGGGTCAC---
TTTGTGTCCCGGCGTTTACTTTGAGAAAATTAGAGTGCTTCAAGCAGGCTTTTGCCTTGAATACTTTAG
CATGGAATAATGGAGTAGGACTTTGGTTCTATTTTGTTGGTTTTAGGAGCCAGAGTAATGGTTAATAGG
AACAGTTGGGGGCATTCGTATTTGACTGTCAGAGGTGAAATTCTTAGATTTGTCAAAGACGAACTACT
GCGAAAGCATTTGCCAAGGATGTTTTCATTAATCAAGAACGAAAGTTAGGGGATCGAAGACGATCAG
ATACCGTCGTAGTCCTAACCATAAACTAA---

>PX369341_VIETNAM

CTGGTGCCAGCAGCCGCGGTAATTCCAGCTCCAATAGCGTATATTAAACTTGTTGCAGTTAAAAAGC
TCGTAGTTGAATTTCTGCTGTATCGTTATCTTCTGC-TTGACAGTTTGGTATCGTTATGGCTT-
GTTGGGTCAC---
TTTGTGTCCCGGCGTTTACTTTGAGAAAATTAAAGTGCTTCAAGCAGGCTTTTGCCTTGAATACTTTAG
CATGGAATAATGGAGTAGGACTTTGGTTCTATTTTGTTGGTTTTAGGAGCCAGAGTAATGGTTAATAGG
AACAGTTGGGGGCATTCGTATTTGACTGTCAGAGGTGAAATTCTTAGATTTGTCAAAGACGAACTACT
GCGAAAGCATTTGCCAAGGATGTTTTCATTAATCAAGAACGAAAGTTAGGGGATCGAAGACGATCAG
ATACCGTCGTAGTCCTAACCATAAACTAA---

>PX369342_VIETNAM

AGCGTATATTAAACTTGTTGCAGTTAAAAAGCTCGTAGTTGAATTTCTGCTGTTTCGTTGACT---
GCGTTTGGCGTTTGTCATTGTTGCGGCTTGGTTGGGTTTCGATTTCGTTTCCCGGCGTTTACTTTGAGA
AAATTAGAGTGCTTGAAGCAGGCTTTTGCCTTGAATACTTTAGCATGGAATAACGGAGTAGGACTTTG
GTTCTATTTTGTTGGTTTTAGGAGCCAGAGTAATGGTTAATAGGAACAGTTGGGGGCATTCGTATTTGA
CTGTCAGAGGTGAAATTCTTAGATTTGTCAAAGACGAACTACTGCGAAAGCATTTGCCAAGGATGTTT
TCATTAATCAAGAACGAAAGTTAGGGGATCGAAGACGATCAGATACCGTCGTAGTCCTAACCATAAA

CTAANNNCN---

>PX369343_VIETNAM

GTGCCAGCAGCCGCGGTAATTCCAGATCCAATAGCGTATATTAAACTTGTTGCAGTTAAAAAGCTCG
TAGTTGAATTTCTGCTGTTTCGTTGACT---
GCGTTTGGCGTTTGTCATCGTTGCGGCTTGGTTGGGTTTCGATTTCGTTTCCCGGCGTTTACTTTGAG
AAAATTAGAGTGCTTGAAGCAGGCTTTTGCCTTGAATACTTTAGCATGGAATAACGGAGTAGGACTTT
GGTTCTATTTTGTTGGTTTTAGGAGCCAGAGTAATGGTTAATAGGAACAGTTGGGGGCATTCGTATTTG
ACTGTCAGAGGTGAAATTCTTATATTTGTCAAAAACGAACTACTGCGAAAGCATTTGCCAAGGATGTTT
TCATTAATCAAGAACGAAAGTTAAGGGATCAAAAACGATCAGATACCGTCGTAGTCCTAACCATAAA
CTAA---

>PX369344_VIETNAM

AGTCTGGTGCCAGCAGCCGCGGTAATTCCAGCTCCAATAGCGTATATTAAACTTGTTGCAGTTAAAA
AGCTCGTAGTTGAATTTCTGCTGTATCGTTATCTTCTGC-TTGACAGTTTGGTATCGTTATGGCTT-
GTTGGGTCAC---
TTTGTGTCCCGGCGTTTACTTTGAGAAAATTAGAGTGCTTCAAGCAGGCTTTTGCCTTGAATACTTTAG
CATGGAATAATGGAGTAGGACTTTGGTTCTATTTTGTTGGTTTTAGGAGCCAGAGTAATGGTTAATAGG
AACAGTTGGGGGCATTCGTATTTGACTGTCAGAGGTGAAATTCTTAGATTTGTCAAAGACGAACTACT
GCGAAAGCATTTGCCAAGGATGTTTTCATTAATCAAGAACGAAAGTTAGGGGATCGAAGACGATCAG
ATACCGTCGTAGTCCTAACCATAAACTA---

>PX369345_VIETNAM

GGGCAGTCTGGTGCCAGCAGCCGCGGTAATTCCAGCTCCAATAGCGTATATTAAACTTGTTGCAGT
TAAAAAGCTCGTAGTTGAATTTCTGCTGTTTCGTTGACT---
GCGTTTGGCGTTTGTCATCGTTGCGGCTTGGTTGGGTTTCGATTTCGTTTCCCGGCGTTTACTTTGAG
AAAATTAGAGTGCTTGAAGCAGGCTTTTGCCTTGAATACTTTAGCATGGAATAACGGAGTAGGACTTT
GGTTCTATTTTGTTGGTTTTAGGAGCCAGAGTAATGGTTAATAGGAACAGTTGGGGGCATTCGTATTTG
ACTGTCAGAGGTGAAATTCTTAGATTTGTCAAAGACGAACTACTGCGAAAGCATTTGCCAAGGATGTT
TTCATTAATCAAGAACGAAAGTTAGGGGATCGAAGACGATCAGATACCGTCGTAGTCCTAACCATAA
ACTAA---

>PX369346_VIETNAM

TGGTGCCAGCAGCCGCGGTAATTCCAGCTCCAATAGCGTATATTAAACTTGTTGCAGTTAAAAAGCT
CGTAGTTGAATTTCTGCTGCATCGTGGTTCTTCGCCATGTCGAGTGATCTTCGTTGTGGCTTAGTTGG
GGCATGTTTTCATGACTCGACGTTTACTTTGAGAAAATTAGAGTGCTTCAAGCAGGCTTTTGCCTTGAA
TACTTTAGCATGGAATAATGGAGTAGGACTTTGGTTCTATTTTGTTGGTTTTAGGAGCCGGAGTAATGG
TTAATAGGAACAGTTGGGGGCATTCGTATTTGACTGTCAGAGGTGAAATTCTTAGATTTGTCAAAGAC
GAACTACTGCGAAAGCATTTGCCAAGGATGTTTCCATTAATCAAGAACGAAAGTTAGGGGATCGAAG
ACGATCAGATACCGTCGTAGTCCTAACCATAAACTAA---

>PX369347_VIETNAM

---CCGCGGT-
ATTCCAGCTCC-
ATAGCGTATATTAAACTTGTTGCAGTTAAAAAGCTCGTAGTTGAATTTCTGCTGTTTCGTTGACT---
GCGTTTGGCGTTTGTCATCGTTGCGGCTTGGTTGGGTTTCGATTTCGTTTCCCGGCGTTTACTTTGAG
AAAATTAGAGTGCTTGAAGCAGGCTTTTGCCTTGAATACTTTAGCATGGAATAACGGAGTAGGACTTT
GGTTCTATTTTGTTGGTTTTAGGAGCCAGAGTAATGGTTAATAGGAACAGTTGGGGGCATTCGTATTTG
ACTGTCAGAGGTGAAATTCTTAGATTTGTCAAAGACGAACTACTGCGAAAGCATTTGCCAAGGATGTT
TTCATTAATCAAGAACGAAAGTTAGGGGATNAAGAACGATCAGATACCGTCGTAGCCCTAACCAATA
A--ACCTAAA-

>PX369348_VIETNAM

GCAGCCGCGGTAATTCCAGCTCCAATAGCGTATATNAAACTTGTTGCAGTTAAAAAGCTCGTAGTTG
AATTTCTGCTGTTTCGTTGACT---
GCGTTTGGCGTTTGTCATCGTTGCGGCTTGGTTGGGTTTCGATTTCGTTTCCCGGCGTTTACTTTGAG
AAAATTAGAGTGCTTGAAGCAGGCTTTTGCCTTGAATACTTTAGCATGGAATAACGGAGTAGGACTTT
GGTTCTATTTTGTTGGTTTTAGGAGCCAGAGTAATGGTTAATAGGAACAGTTGGGGGCATTCGTATTTG
ACTGTCAGAGGTGAAATTCTTAGATTTGTCAAAAACGAACTACTGCGAAAGCATTTGCCAAGGATGTT
TTCATTAATCAAGAACGAAAGTTAGGGGATCGAAAACGATCAGATACCGTCGTAGTCCTAACCATAA
ACTA--

>PX369349_VIETNAM

GGTGCCAGCAGCCGCGGTAATTCCAGCTCCAATAGCGTATATNAAACTTGTTGCAGTTAAAAAGCT
CGTAGTTGAATTTCTGCTGTTTCGTTGACT---
GCGTTTGGCGTTTGTCATCGTTGCGGCTTGGTTGGGTTTCGATTTCGTTTCCCGGCGTTTACTTTGAG
AAAATTAGAGTGCTTGAAGCAGGCTTTTGCCTTGAATACTTTAGCATGGAATAACGGAGTAGGACTTT
GGTTCTATTTTGTTGGTTTTAGGAGCCAGAGTAATGGTTAATAGGAACAGTTGGGGGCATTCGTATTTG
ACTGTCAGAGGTGAAATTCTTAGATTTGTCAAAGACGAACTACTGCGAAAGCATTTGCCAAGGATGTT
TTCATTAATCAAGAACGAAAGTTAGGGGATCGAAGACGATCAGATACCGTCGTAGTCCTAACCATAA
ACTAA---

>PX369350_VIETNAM

TCTGGTGCCAGCAGCCGCGGTAATTCCAGCTCCAATAGCGTATATTAAACTTGTTGCAGTTAAAAAG
CTCGTAGTTGAATTTCTGCTGTTTCGTTGACT---
GCGTTTGGCGTTTGTCATCGTTGCGGCTTGGTTGGGTTTCGATTTCGTTTCCCGGCGTTTACTTTGAG
AAAATTAGAGTGCTTGAAGCAGGCTTTTGCCTTGAATACTTTAGCATGGAATAACGGAGTAGGACTTT
GGTTCTATTTTGTTGGTTTTAGGAGCCAGAGTAATGGTTAATAGGAACAGTTGGGGGCATTCGTATTTG
ACTGTCAGAGGTGAAATTCTTAGATTTGTCAAAGACGAACTACTGCGAAAGCATTTGCCAAGGATGTT
TTCATTAATCAAGAACGAAAGTTAGGGGATCGAAGACGATCAGATACCGTCGTAGTCCTAACCATAA
ACTAA--

>PX369351_VIETNAM

TGGTGCCAGCAGCCGCGGTAATTCCAGCTCCAATAGCGTATATTAAACTTGTTGCAGTTAAAAAGCT
CGTAGTTGAATTTCTGCTGTTTCGTTGACT---
GCGTTTGGCGTTTGTCATCGTTGCGGCTTGGTTGGGTTTCGATTTCGTTTCCCGGCGTTTACTTTGAG
AAAATTAGAGTGCTTGAAGCAGGCTTTTGCCTTGAATACTTTAGCATGGAATAACGGAGTAGGACTTT
GGTTCTATTTTGTTGGTTTTAGGAGCCAGAGTAATGGTTAATAGGAACAGTTGGGGGCATTCGTATTTG
ACTGTCAGAGGTGAAATTCTTAGATTTGTCAAAGACGAACTACTGCGAAAGCATTTGCCAAGGATGTT
TTCATTAATCAAGAACGAAAGTTAGGGGATCGAAGACGATCAGATACCGTCGTAGTCCTAACCATAA
ACTAA--

>PX369352_VIETNAM

GGTGCCAGCAGCCGCGGTAATTCCAGCTCCAATAGCGTATATTAAACTTGTTGCAGTTAAAAAGCTC
GTAGTTGAATTTCTGCTGCATCGTGGTTCTTCGCTATGTCGAGTGATCTTCGTTGTGGCTTAGTTGGG
GCATGTTTTCATGACTCGACGTTTACTTTGAGAAAATTAGAGTGCTTCAAGCAGGCTTTTGCCTTGAAT
ACTTTAGCATGGAATAATGGAGTAGGACTTTGGTTCTATTTTGTTGGTTTTAGGAGCCGGAGTAATGGT
TAATAGGAACAGTTGGGGGCATTCGTATTTGACTGTCAGAGGTGAAATTCTTAGATTTGTCAAAGACG
AACTACTGCGAAAGCATTTGCCAAGGATGTTTCCATTAATCAAGAACGAAAGTTAGGGGATCGAAGA
CGATCAGATACCGTCGTAGTCCTAACCATAAACTAA---

>PX369353_VIETNAM

NGTCTGGTGCCAGCAGCCGCGGTAATTCCAGCTCCAATAGCGTATATTAAACTTGTTGCAGTTAAAA
AGCTCGTAGTTGAATTTCTGCTGCATCGTGGTTCTTCGCTATGTCGAGTGATCTTCGTTGTGGCTTAGT
TGGGGCATGTTTTCATGACTCGACGTTTACTTTGAGAAAATTAGAGTGCTTCAAGCAGGCTTTTGCCTT
GAATACTTTAGCATGGAATAATGGAGTAGGACTTTGGTTCTATTTTGTTGGTTTTAGGAGCCGGAGTAA
TGGTTAATAGGAACAGTTGGGGGCATTCGTATTTGACTGTCAGAGGTGAAATTCTTAGATTTGTCAAAG
ACGAACTACTGCGAAAGCATTTGCCAAGGATGTTTCCATTAATCAAGAACGAAAGTTAGGGGATCGA
AGACGATCAGATACCGTCGTAGTCCTAACCATAAACTA--

SUPPORTING INFORMATION

USED SEQUENCES CLADE ASSORTMENTS 2
FINAL ALIGNMENT 4
MODEL SELECTION 45
ML ANALYSIS 47
ML TREE 50
QUESTIONNAIRE FOR HORSES 53

MODEL SELECTION

```
Starting SMS v1.8.1
~~~~~~~~~~~~~~~~~~~~~~~~~~~~~~~~~~~~~~~~~~~~~~~~~~~~~~~~~~~~~~~~
Input alignment    : align_rename.phy
Data type          : DNA
Number of taxa     : 80
Number of sites    : 1528
Number of branches : 157
Criterion          : AIC
~~~~~~~~~~~~~~~~~~~~~~~~~~~~~~~~~~~~~~~~~~~~~~~~~~~~~~~~~~~~~~~~
Step 1 : Set a fixed topology
        AIC=9750.87332
~~~~~~~~~~~~~~~~~~~~~~~~~~~~~~~~~~~~~~~~~~~~~~~~~~~~~~~~~~~~~~~~
Step 2 : Select the best decoration
        AIC=9750.87332      decoration : '+G+I'
~~~~~~~~~~~~~~~~~~~~~~~~~~~~~~~~~~~~~~~~~~~~~~~~~~~~~~~~~~~~~~~~
Step 3 : Select the best matrix
        AIC=0   matrix : 'TN93'
~~~~~~~~~~~~~~~~~~~~~~~~~~~~~~~~~~~~~~~~~~~~~~~~~~~~~~~~~~~~~~~~
Step 4 : Select the best final decoration
        AIC=9750.23992      decoration : '+G+I'
~~~~~~~~~~~~~~~~~~~~~~~~~~~~~~~~~~~~~~~~~~~~~~~~~~~~~~~~~~~~~~~~
Selected model                              : TN93 +G+I
~~~~~~~~~~~~~~~~~~~~~~~~~~~~~~~~~~~~~~~~~~~~~~~~~~~~~~~~~~~~~~~~
Substitution model                   : TN93
Equilibrium frequencies                     : ML optimized
Proportion of invariable sites       : estimated (0.456)
Number of substitution rate categories      : 4
Gamma shape parameter                       : estimated (0.773)
```

SUPPORTING INFORMATION

USED SEQUENCES CLADE ASSORTMENTS 2
FINAL ALIGNMENT 4
MODEL SELECTION 45
ML ANALYSIS 47
ML TREE 50
QUESTIONNAIRE FOR HORSES 53

ML ANALYSIS

--- PhyML 20120412 ---

http://www.atgc-montpellier.fr/phyml

Copyright CNRS - Universite Montpellier II

oooooooooooooooooooooooooooooooooooooooooooooooooooooooooooooooooooooooooooooooooooooooooooooooooo

. Sequence filename: align_rename.phy

. Data set: #1

. Tree topology search : SPRs

. Initial tree: BioNJ

. Model of nucleotides substitution: TN93

. Number of taxa: 80

. Log-likelihood: -4691.88261

. Unconstrained likelihood: -5440.13936

. Parsimony: 412

. Tree size: 0.33784

. Discrete gamma model: Yes

- Number of categories: 4

- Gamma shape parameter: 0.773

. Proportion of invariant: 0.456

. Transition/transversion ratio for purines: 2.196

. Transition/transversion ratio for pyrimidines: 3.155

. Nucleotides frequencies:

- f(A)= 0.25822

- f(C)= 0.18905

- f(G)= 0.26810

- f(T)= 0.28463

. Run ID:	none
. Random seed:	1767967051
. Subtree patterns aliasing:	no
. Version:	20120412
. Time used:	0h1m4s (64 seconds)

SUPPORTING INFORMATION

USED SEQUENCES CLADE ASSORTMENTS 2

FINAL ALIGNMENT 4

MODEL SELECTION 45

ML ANALYSIS 47

ML TREE 50

QUESTIONNAIRE FOR HORSES 53

ML TREE

(((HQ895974-1_Theileria_parva:0.00194735,HQ895984-
1_Theileria_parva:0.00038138):0.0000002[0],(HQ895985-
1_Theileria_parva:0.00067112,(HQ895973-1_Theileria_parva:0.00000003,HQ895975-
1_Theileria_parva:0.00000001):0.00000004[0]):0.00036175[0]):0.03600821,((((EU888903_South
-Africa:0.00127969,((KY464032_Brazil:0.00129582,(PX369341-
1_VIETNAM:0.00216815,(PX369340-1_VIETNAM:0.00000014,PX369344-
1_VIETNAM:0.00000001):0.00000001[0]):0.00657073[0.966]):0.000043[0],(EU888905-
1_Theileria_equi:0.00067509,(KU647706-
1_Theileria_haneyi:0.00069188,(MG052901_Brazil:0.00000001,((KU647708-
1_Theileria_haneyi:0.00000005,KU647707-
1_Theileria_haneyi:0.00000001):0.00000003[0],(KU240070_Brazil:0.00000001,((KY111760_Cub
a:0.00066667,KU647705-
1_Theileria_haneyi:0.00000001):0.00000004[0],(JQ390047_Mexico:0.00000001,(EU642511_Sou
th-Africa:0.00134116,(KU240071_Brazil:0.00000001,(KX722513_Brazil:0.00133647,KU647704-
1_Theileria_haneyi:0.00138408):0.00000003[0]):0.00000004[0]):0.00000004[0]):0.00000001[0]):
0.00000004[0]):0.00000004[0]):0.00000001[0]):0.00000005[0]):0.00000006[0]):0.00000006[0]):
0.00211646[0.905]):0.00651714[0.922],((AB515311_Sudan:0.00104569,EU642509_South-
Africa:0.00379306):0.00136231[0.352],((AB515312_Sudan:0.00066057,(KX227627_Israel:0.009
54869,(AB515314_Sudan:0.00000003,AB515313_Sudan:0.003337):0.00198662[0.942]):0.00000
004[0]):0.00066037[0.768],((MG569895_Turkey:0.00000007,MG569900_Turkey:0.00000001):0.
00000001[0],(KX227636_Israel:0.02705097,KX227624_Israel:0.00000005):0.00065956[0.798]):0
.00065856[0.783]):0.00170179[0.851]):0.00449056[0.827]):0.01659836[0.996],(EU888902_Sout
h-
Africa:0.00441883,(((MG569904_Turkey:0.00000001,(KX227638_Israel:0.00000003,(KU672386_
USA:0.00065978,KY464035_Brazil:0.00743792):0.00000004[0]):0.00000002[0]):0.00000003[0],(
KY111762_Cuba:0.00065947,(JX177670_USA:0.00000003,(PV688153_VIETNAM:0.00000015,MK
392050_Israel:0.00000001):0.00000001[0]):0.00000004[0]):0.00065959[0.854]):0.00000001[0],(
KY952230_Brazil:0.00000004,((PX369351-
1_VIETNAM:0.00000011,PV688151_VIETNAM:0.00000001):0.0000001[0],(PX369350-
1_VIETNAM:0.00000001,((PX369349-
1_VIETNAM:0.00000012,PV688150_VIETNAM:0.00000001):0.00000011[0],((PV688149_VIETNA
M:0.00000079,PX369342-1_VIETNAM:0.00257867):0.00245818[0.63],(PX369345-
1_VIETNAM:0.00000001,(PX369347-1_VIETNAM:0.01229791,(PX369343-
1_VIETNAM:0.00863258,PX369348-
1_VIETNAM:0.0000001):0.00212057[0.293]):0.00230252[0.771]):0.00000001[0]):0.00525061[0.
746]):0.00000017[0]):0.00000014[0]):0.00517816[0.942]):0.00000004[0]):0.00096274[0.75]):0.0
1701729[0.998]):0.00815293[0.103],((EU642507_South-
Africa:0.00116414,((KF597077_Kenya:0.00197331,AB515310_Sudan:0.0033183):0.00000007[0]
,(KF597073_Kenya:0.00197809,(KF597081_Kenya:0.00331206,KF597078_Kenya:0.00197205):0.
00000007[0]):0.00000007[0]):0.0008139[0.766]):0.00279292[0.531],(KM046920_Switzerland:0.
00596924,((PV688152_VIETNAM:0.00000001,((PX369353-
1_VIETNAM:0.00000001,PV688154_VIETNAM:0.00000001):0.0000001[0],((PV688146_VIETNAM:
0.0000001,PX369352-
1_VIETNAM:0.00000001):0.0000001[0],(PV688148_VIETNAM:0.00000001,(PV688145_VIETNAM:

0.00000001,(PX369346-
1_VIETNAM:0.00207953,PV688147_VIETNAM:0.00000021):0.00000021[0]):0.00000022[0]):0.00
000022[0]):0.00688257[0]):0.00124407[0]):0.00405443[0.832],((KF559357_China:0.00000002,L
C781882_Saudi-
Arabia:0.00000001):0.00000006[0],(KM046919_Switzerland:0.00263784,((KM046922_Hungary:
0.01716106,AY534882_Spain:0.00065806):0.00065786[0.775],((KM046918_Switzerland:0.0026
3883,HM229407_South-Korea:0.00000001):0.00000006[0],(HM229408_South-
Korea:0.00000001,(LC431546_Saudi-
Arabia:0.00070618,DQ287951_Spain:0.00531739):0.00000003[0]):0.00000003[0]):0.00000006[
0]):0.00000007[0]):0.00000007[0]):0.00000042[0]):0.00000021[0]):0.00535716[0.931]):0.00934
356[0.886]):0.03600821[1]);

SUPPORTING INFORMATION

USED SEQUENCES CLADE ASSORTMENTS 2
FINAL ALIGNMENT 4
MODEL SELECTION 45
ML ANALYSIS 47
ML TREE 50
QUESTIONNAIRE FOR HORSES 53

QUESTIONNAIRE FOR HORSES

Date: **Code number:**

Season: ☐ **spring** ☐ **summer** ☐ **autumn** ☐ **winter**

Province: ☐ **Hanoi** ☐ **Thai Nguyen** ☐ **Son La**

Name of district:

Breed:

Age: ☐ **month** ☐ **year**

Sex: ☐ **female** ☐ **male** ☐ **neutered**

Number of horses kept together:

Clinical examination: ☐ **healthy** ☐ **clinical signs:**

Tick infestation: ☐ **yes** **number of collected ticks:**

☐ **no**

Control of ticks/other ectoparasites: ☐ **no** ☐ **yes**

Date of last treatment:

Product name:

Keeping system: ☐ **alone** ☐ **with other animals**

☐ **indoor** ☐ **outdoor/pasture**

with ☐ **horses** ☐ **cattle** ☐ **water buffalo** ☐ **sheep** ☐ **goat**

Remarks:
